# Parkinson’s disease-associated PLA2G6 protects IP3R1 protein to control ER-mitochondria tethering and Ca^2+^ transfer

**DOI:** 10.1038/s41467-026-70752-1

**Published:** 2026-03-19

**Authors:** Zhi-Hao Lin, Nai-Jia Xue, Yi Liu, Feng Zhang, Xiao-Li Si, Ran Zheng, Lu-Yan Gu, Yao-Lin Li, Yi Fan, Jun Tian, Wolfgang H. Oertel, Hyemyung Seo, Jia-Li Pu, Bao-Rong Zhang

**Affiliations:** 1 Department of Neurology, Second Affiliated Hospital, School of Medicine, Zhejiang University, Hangzhou, China; 2 Department of Neurology, Philipps University Marburg, Marburg, Germany; 3 Department of Molecular & Life Sciences, Center for Bionano Intelligence Education and Research, Hanyang University, Ansan, South Korea

**Keywords:** Mechanisms of disease, Mitochondria, Parkinson's disease

## Abstract

Mutations in the phospholipase A2 group VI (*PLA2G6*) gene have been linked to autosomal recessive Parkinson’s disease (PD), yet the molecular mechanisms remain poorly understood. This study provides the in vitro and in vivo evidence, specifically in dopaminergic neurons derived from patients with PD, that PLA2G6 loss-of-function disrupts the mitochondria-associated endoplasmic reticulum (ER) membrane (MAM), a critical regulator of Ca^2+^ transfer and energy homeostasis. This study demonstrates that the PLA2G6 protein localizes to the MAM and physically associates with the IP3R1-GRP75-VDAC1 complex. PLA2G6 deficiency destabilizes this complex, accelerating IP3R1 degradation, which in turn reduces ER-mitochondria contacts and impairs Ca^2+^ transfer. Notably, introducing a MAM linker restores the phenotypes caused by PLA2G6 loss. In iPSCs-derived dopaminergic neurons from patients with PD harboring *PLA2G6* mutations, the structural and functional disruption of the MAM is further confirmed, underscoring its role in PD pathogenesis. These findings uncover the pivotal function of PLA2G6 within the MAM and suggest that modulating inter-organelle contacts could be a therapeutic strategy for correcting PD’s ion channel dysfunction and energy imbalances.

## Abstract (web summary)

Here, the authors show that the PLA2G6 protein is a component of the IP3R1–GRP75–VDAC1 complex at the mitochondria-associated endoplasmic reticulum (ER) membrane, maintaining the integrity and function of ER–mitochondria contacts.

## Introduction

Parkinson’s disease (PD), one of the most prevalent neurodegenerative disorders, is marked by the progressive loss of dopaminergic neurons in the substantia nigra pars compacta and remains without a curative treatment^1^. While most PD cases are sporadic, 5–10% are familial, linked to mutations in the *PARK* loci. However, both sporadic and familial forms exhibit similar phenotypes, suggesting potential shared underlying mechanisms^2^. A critical cellular feature in PD is the simultaneous dysfunction of the endoplasmic reticulum (ER) and mitochondria^3–6^, with mutations in several PD-related genes contributing to these organelle disturbances^7,8^. Existing therapies targeting either the ER or mitochondria have shown limited success, prompting the hypothesis that ER-mitochondria contact sites may play a pivotal role in PD pathogenesis^9^.

The mitochondria-associated ER membrane (MAM) is a specialized subdomain of the ER membrane that is closely juxtaposed (within 30 nm) to the mitochondrial outer membrane^10^. Several molecular complexes, such as IP3R-GRP75-VDAC1, ERMIT2-MFN2, VAPB-PTPIP51, and BAP31-Fis1, are responsible for tethering the MAM^10–14^. MAM regulates key cellular processes, including phospholipid synthesis, Ca^2+^ transfer, ER stress, mitochondrial quality control, autophagy, and ferroptosis^10,15,16^.

Genetic studies have identified at least 54 mutations in the phospholipase A2 group VI gene (*PLA2G6*, also referred to as *PARK14*), which encodes calcium-independent phospholipase A2β (iPLA2β, also known as iPLA2-VIA or PLA2G6), as contributors to *PLA2G6*-related dystonia-parkinsonism^17–19^. Previous studies have shown that PLA2G6 localizes to both the ER and mitochondria, playing a pivotal role in maintaining neuronal ER and mitochondrial homeostasis^20,21^. While MAM dysfunction has been implicated in neurodegenerative diseases involving α-Synuclein, DJ-1, and Parkin-linked PD^22–24^, whether PLA2G6 specifically localizes to the MAM and facilitates ER-mitochondria communication has yet to be determined.

This study identified MAM dysfunction in *Pla2g6* knockout (KO) N2a cells, mouse brains, and dopaminergic neurons derived from patients with PD harboring *PLA2G6* mutations, leading to impaired mitochondrial Ca^2+^ uptake and ATP production. This dysfunction arises from the disruption of the IP3R1-GRP75-VDAC1 complex and accelerated IP3R1 degradation due to PLA2G6 deficiency. These results reveal a molecular mechanism contributing to PLA2G6-linked PD pathology.

## Results

### PLA2G6 protein is localized in the MAM

**Fig. 1:**
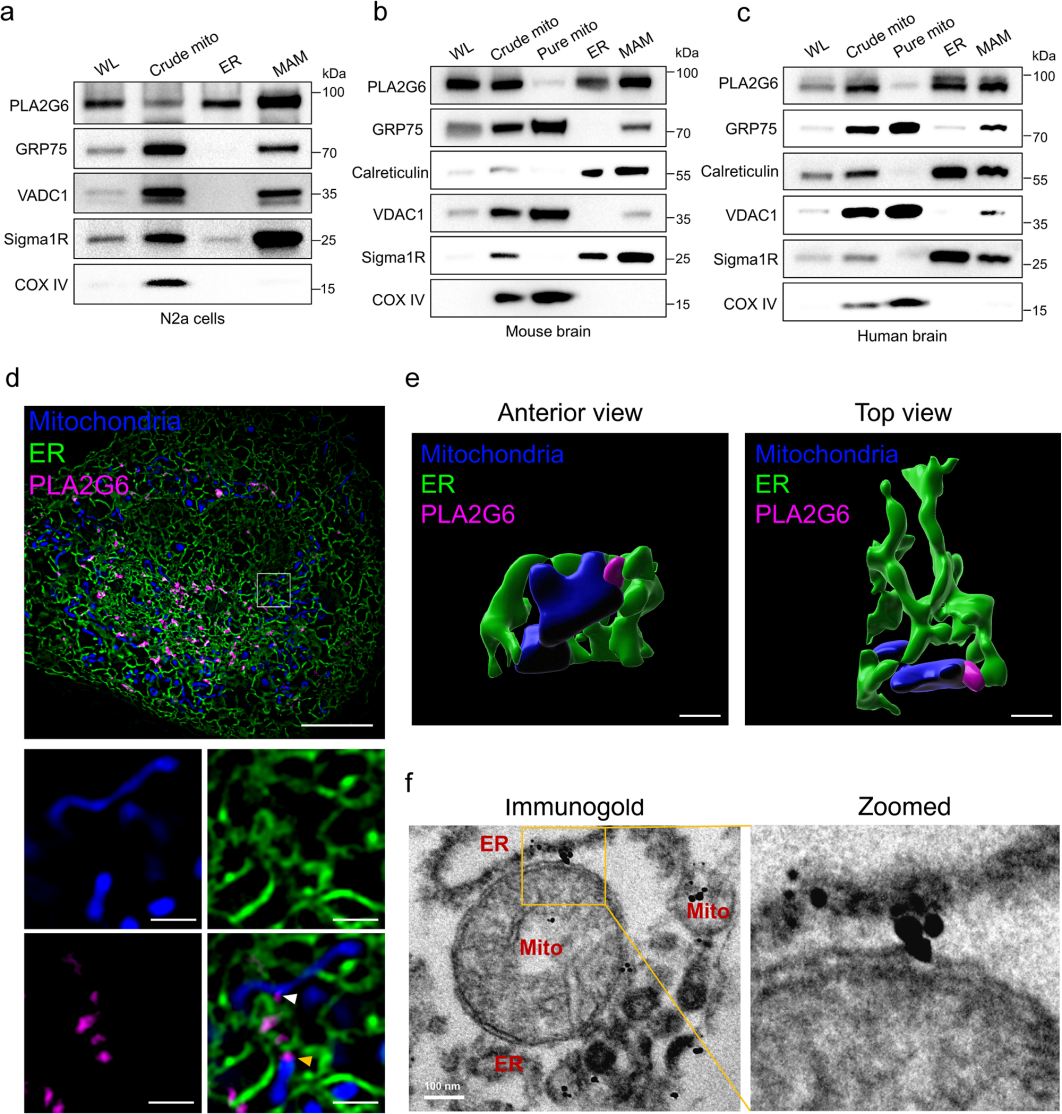
Enrichment of PLA2G6 in MAM. **a** Immunoblotting analysis of subcellular fractions from normal N2a cells (*n* = 3 biologically independent experiments). **b** Immunoblotting analysis of subcellular fractions from the ventral midbrains of male WT C57BL/6 mice (*n* = 3 biologically independent experiments). **c** Immunoblotting analysis of subcellular fractions from human brains (*n* = 2 biologically independent experiments). **d** High intelligent and sensitive structured illumination microscopy (HIS-SIM) demonstrating the localization of PLA2G6 (magenta) in ER (green)-mitochondria (blue) contacts indicated by the arrowheads using U2OS cells transiently transfected with pTagBFP-Mito, pEGFP-ER, and pDsRed-PLA2G6. (scale bar, 10 μm, *n* = 3 independent experiments). White boxes in the image are magnified below. **e** 3D reconstructions of PLA2G6 (magenta), ER (green), and mitochondria (blue) indicated by the yellow arrowhead in this figure (**d**) using Imaris software (scale bar, 0.5 μm; see Supplementary Movie 1 for the original data). **f** Representative immune electron microscopy images showing N2a cells immunolabeled with anti-PLA2G6 antibody. The highlighted yellow box is magnified on the right (*n* = 2 independent experiments) (scale bar, 100 nm). WL whole lysates; crude mito, crude mitochondria; pure mito, purified mitochondria, ER endoplasmic reticulum, MAM mitochondria-associated ER membrane.

To explore PLA2G6’s potential role in ER-mitochondria contacts, we first determined whether PLA2G6 is present in the MAM. Subcellular organelles, including mitochondria, ER, and MAM, were isolated from N2a cells, mouse ventral midbrains, and human brain tissues using Percoll-based ultracentrifugation. The identity and purity of these fractions were confirmed through immunoblotting with organelle-specific markers: Sigma1R and calreticulin for ER/MAM, VDAC1 and GRP75 for mitochondria/MAM, and COX IV for mitochondria. Endogenous PLA2G6 is localized in the MAM fraction (Fig. 1a–c). In addition, as previously reported, the PLA2G6 protein was also partially detected in the mitochondria and ER fractions^20^. Further validation of PLA2G6 localization was achieved through immunofluorescence using highly intelligent and sensitive structured illumination microscopy (HIS-SIM), revealing that some puncta-like staining of PLA2G6 (magenta) protein was specifically at ER (green)-mitochondria (blue) contact points (Fig. 1d, e and Supplementary Movie 1). Additionally, immuno-electron microscopy in N2a cells confirmed PLA2G6’s presence at ER-mitochondria junctions (Fig. 1f). Collectively, these complementary experiments provide robust evidence of PLA2G6’s localization within the MAM.

### The ER-mitochondria contacts were decreased after PLA2G6 absence

**Fig. 2:**
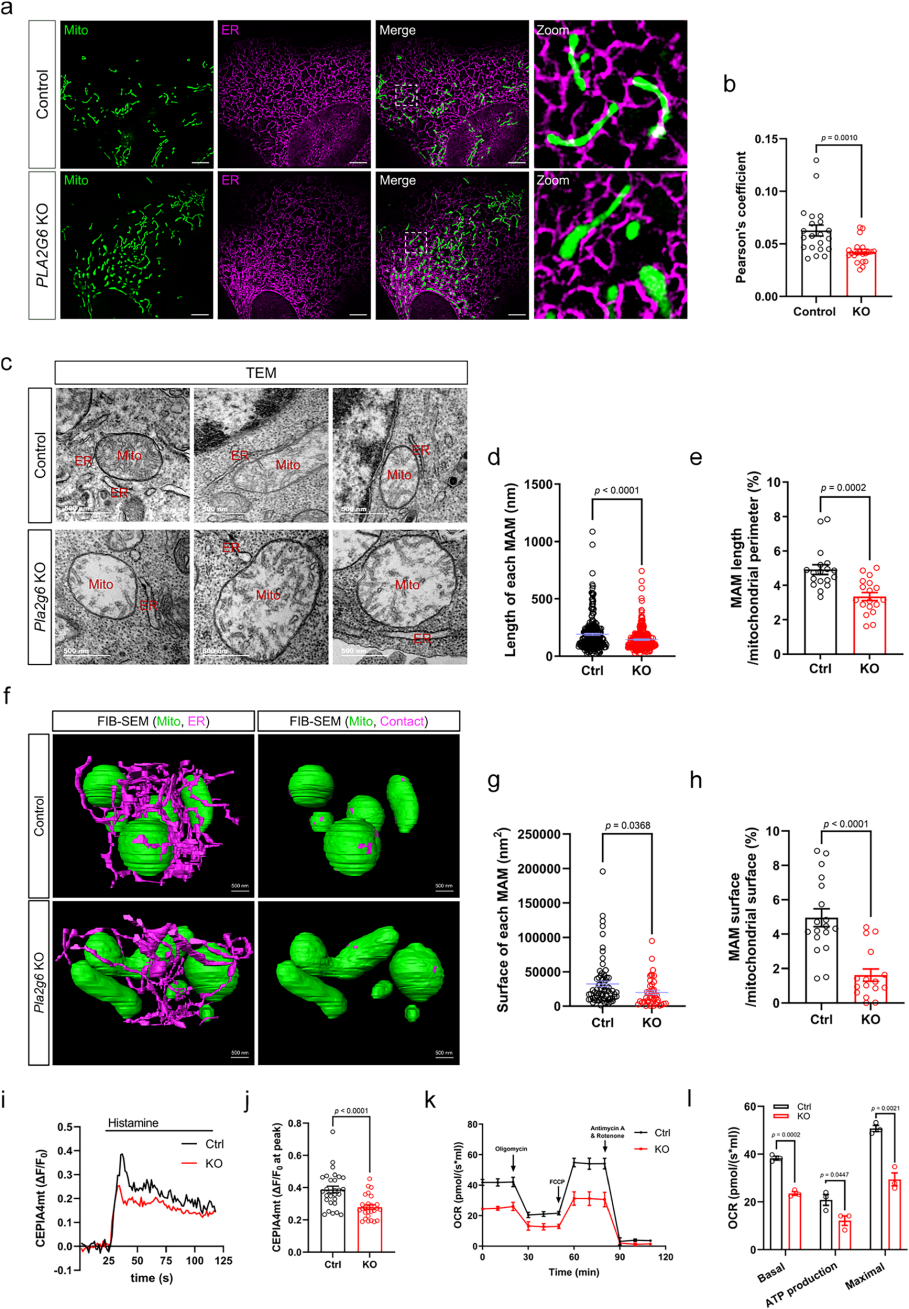
PLA2G6 deficiency reduces the ER-mitochondria contacts, mitochondrial Ca^2+^ import, and ATP production. **a** HIS-SIM analysis of ER (magenta)-mitochondria (green) interactions. Control and *PLA2G6* KO U2OS cells were transfected with pEGFP-Mito and pDsRed-ER. White dotted boxes in the images are magnified on the right (scale bar, 5 μm). **b** Quantification of ER-mitochondria colocalization using Pearson’s coefficient (control, *n* = 21 cells; KO, *n* = 21 cells from three independent experiments). **c** Representative TEM micrographs of control and *Pla2g6* KO N2a cells (scale bar, 500 nm). **d**, **e** Measurement of MAM length (nm) and MAM length/mitochondrial perimeter (%) in control and *Pla2g6* KO N2a cells (control, *n* = 18 cells and 492 mitochondria; KO, *n* = 18 cells and 418 mitochondria from three independent experiments). **f** 3D ultrastructural analysis of ER-mitochondria contacts in control and *Pla2g6* KO N2a cells using FIB-SEM. Mitochondria are labeled green, and ER and MAM are labeled magenta (scale bar, 500 nm; see Supplementary Movie 2 and 3 for original data). **g**, **h** Measurement of the surface of each MAM and MAM surface/mitochondrial surface (%) in control and *Pla2g6* KO N2a cells. Total MAM count identified from serial electron microscope reconstructions: 72 in control and 41 in *Pla2g6* KO, from 18 control and 16 *Pla2g6* KO mitochondria fully reconstructed in three independent experiments. **i** Representative mitochondrial Ca^2+^ traces following histamine (200 μM) stimulation in control and *Pla2g6* KO N2a cells, measured using the CEPIA4mt plasmid. **j** Quantification of histamine-stimulated Ca^2+^ peak values (control, *n* = 29 cells; *Pla2g6* KO, *n* = 27 cells from three independent experiments). **k** OCR measured in control and *Pla2g6* KO N2a cells (*n* = 3 biologically independent experiments). **l** Quantification of basal OCR, ATP production, and maximal OCR for two million cells per group (*n* = 3 biologically independent experiments). Data are presented as means ± SEM. Two-tailed unpaired *t*-tests were used for statistical analysis (**b**, **d**, **e**, **g**, **h**, **j**, **l**). Mito mitochondria, ER endoplasmic reticulum, TEM transmission electron microscopy, FIB-SEM focus ion beam-scanning electron microscopy, OCR oxygen consumption rate.

Given PLA2G6’s localization in the MAM, it was hypothesized that the protein may contribute to preserving MAM integrity and function. HIS-SIM analysis revealed that colocalization of the mitochondria (green) and ER (magenta) was reduced in *PLA2G6* KO U2OS cells compared to control cells (Fig. 2a, b and Supplementary Fig. 1a). To further corroborate this finding, quantitative transmission electron microscopy (TEM) was employed to assess the MAM length of individual MAM (defined as the proximity between ER and mitochondrial membranes within 30 nm) and the ratio of MAM length to mitochondrial perimeter. In control N2a cells, the average MAM length was 189.9 ± 8.9 nm, which decreased to 145.2 ± 6.6 nm in *Pla2g6* KO cells (Fig. 2c, d and Supplementary Fig. 1b). Additionally, the ratio of MAM length to mitochondrial perimeter was reduced from 4.92% ± 0.28% in control cells to 3.35% ± 0.24% in *Pla2g6* KO cells (Fig. 2c, e).

To further examine ER-mitochondria contacts at the three-dimensional (3D) level, focus ion beam-scanning electron microscopy (FIB-SEM) was employed. This approach provided a 3D view of the interactions between mitochondria (green) and the ER (magenta) (Fig. 2f and Supplementary Movies 2 and 3). In *Pla2g6* KO N2a cells, the surface of ER-mitochondria contacts decreased by 38.5% compared to control cells (Fig. 2g). The MAM surface-to-mitochondrial surface ratio in control cells was 4.96% ± 0.52%, while in *Pla2g6* KO cells, this ratio dropped to 1.62% ± 0.37% (Fig. 2h). Since mitochondrial membrane proteins may influence ER-mitochondria contacts, further investigation into the effects of PLA2G6 deficiency on mitochondrial membrane protein expression was conducted. Western blot (WB) analysis revealed no significant changes in the levels of MFN2, TOM40, VDAC1, TIM23, or TOM20 between control and *Pla2g6* KO N2a cells (Supplementary Fig. 1c, d). Notably, ER-lysosome and mitochondria-lysosome contacts remained unaffected in *PLA2G6* KO cells (Supplementary Fig. 1e–h). These results demonstrate that PLA2G6 deficiency specifically disrupts ER-mitochondria contacts.

In vivo analysis further corroborated these results (Supplementary Fig. 2a). HIS-SIM assays showed a reduction in the colocalization of mitochondria and ER in the dopaminergic neurons of *Pla2g6* KO mice compared to wild-type (WT) mice (Supplementary Fig. 2b, c). To specifically examine the role of PLA2G6 in regulating ER-mitochondria contacts within dopaminergic neurons, we performed tyrosine hydroxylase (TH) immunolabeling to identify dopaminergic neurons in the substantia nigra, followed by comparative analysis of ER-mitochondria interaction between WT and KO mice. We revealed that MAM length in the substantia nigra dopaminergic neurons of WT mice averaged 247.1 ± 9.3 nm, while this was reduced to 130.7 ± 3.9 nm in *Pla2g6* KO mice (Supplementary Fig. 2d, e). Additionally, the MAM length-to-mitochondrial perimeter ratio decreased from 7.69% ± 0.38% in WT mice to 4.77% ± 0.21% in *Pla2g6* KO mice (Supplementary Fig. 2d, f). Collectively, these results indicate that PLA2G6 is essential for stabilizing ER-mitochondria contacts both in vitro and in vivo.

The PLA2G6 protein is composed of ankyrin repeat (ANK) domains and patatin-like phospholipase (PNPLA) domain (refer to the UniProt). To investigate which domain is involved in maintaining the stability of MAM, we generated ANK and PNPLA truncated mutants. Overexpression of WT or PNPLA truncated mutant (ΔPNPLA) in *PLA2G6* KO cells restores the ER-mitochondria contacts, but this interaction was not rescued by ANK truncated mutant (Supplementary Fig. 3a–e). These results indicate that the ANK domain of the PLA2G6 protein is essential for MAM formation.

### Ca^2+^ transfer from the ER to mitochondria and mitochondrial ATP production were repressed after PLA2G6 silencing

MAM is recognized as playing a key role in Ca^2+^ transfer between the ER and mitochondria^25–27^. Given that PLA2G6 deficiency impairs ER-mitochondria contacts, its impact on Ca^2+^ transfer from the ER to mitochondria was further explored. N2a cells were transfected with Ca^2+^-specific fluorescent indicators targeting the mitochondria (CEPIA4mt) and ER (R-CEPIA1er)^28^. Stimulation with extracellular histamine (200 μM), known to generate inositol 1,4,5-trisphosphate (IP3) and activate the IP3 receptor 1 (IP3R1), facilitated Ca^2+^ transfer from the ER to mitochondria^29^. Notably, mitochondrial Ca^2+^ import was significantly reduced in *Pla2g6* KO N2a cells compared to controls following histamine stimulation (Fig. 2i, j). Additionally, Ca^2+^ release from the ER was significantly reduced in *Pla2g6* KO N2a cells (Supplementary Fig. 4a, b). To determine whether the transfer of Ca^2+^ mediated by polycystic kidney disease 2 (PKD2) channels or ryanodine receptors within the MAM is influenced by PLA2G6, the PKD2-specific agonist triptolide^30^ and the ryanodine receptor agonist caffeine^31^ were utilized to stimulate Ca^2+^ transfer from the ER to mitochondria. Notably, no significant changes in mitochondrial Ca^2+^ import were detected following triptolide or caffeine stimulation in both *Pla2g6* KO N2a cells and control cells (Supplementary Fig. 4c–f). These results indicate that PLA2G6 predominantly regulates Ca^2+^ transfer from the ER to mitochondria through the IP3R1 channel. Previous studies have indicated that mitochondrial respiration and ATP production are dependent on mitochondrial Ca^2+^ levels^32–34^. Consequently, the impact of PLA2G6 deficiency on cellular bioenergetics was further examined by measuring the oxygen consumption rate (OCR). The results revealed that PLA2G6 deficiency led to decreased basal respiration, ATP production, and maximal respiration in comparison to control cells (Fig. 2k, l).

Since PLA2G6 is also involved in the regulation of α-synuclein (α-Syn) aggregation and lipid peroxidation^19^, it is highly questionable whether these phenotypes can be explained solely by the reduction in Ca^2+^ import into mitochondria. To address this issue, we used HEK293 cells stably transfected with α-Syn (α-Syn-HEK293 cells) as reporter cells. Transduction of α-Syn-HEK293 cells with α-Syn preformed fibrils (PFFs) induced α-Syn aggregation and phosphorylation (pS129). This effect was potentiated by mitochondrial calcium uniporter (MCU) inhibitors such as ruthenium red (RR) or MCU-i4, which reduced mitochondrial calcium import and consequently enhanced both α-Syn aggregation and phosphorylation (Supplementary Fig. 5a, b). A similar phenomenon was found in cultured primary neurons (Supplementary Fig. 5c–f). To determine the effect of reduced mitochondrial Ca^2+^ import on lipid peroxidation, we stained cells using the BODIPY 581/591 C11 probe. The result indicated that reduced mitochondrial Ca^2+^ import did not affect the lipid peroxidation (Supplementary Fig. 5g, h).

### ER-mitochondria Linker expression rescues MAM and mitochondrial dysfunction in PLA2G6-deficient cells

To determine whether the reduced mitochondrial Ca^2+^ import and ATP production in PLA2G6-deficient cells were indeed caused by disrupted ER-mitochondria associations, the ER-mitochondria Linker was overexpressed to promote MAM formation^35–37^. The specificity of the Linker was validated by confirming its localization at ER-mitochondria contact sites (Supplementary Fig. 6a, b). Overexpression of the Linker successfully restored ER-mitochondria contacts in *Pla2g6* KO N2a cells to levels observed in control cells (Supplementary Fig. 6c–e). Previous studies have implicated PLA2G6 in the regulation of mitochondrial morphology and membrane potential^21,38^, and consistent with these results, mitochondria in *Pla2g6* KO N2a cells appeared enlarged, with reduced membrane potential compared to controls (Supplementary Fig. 6c, f–h). Notably, the ER-mitochondria Linker partially reversed mitochondrial enlargement and improved membrane potential in *Pla2g6* KO cells by enhancing ER-mitochondria interactions (Supplementary Fig. 6c, f–h). Moreover, the Linker restored mitochondrial Ca^2+^ import in *Pla2g6* KO N2a cells (Supplementary Fig. 6i, j). Overexpression of the Linker plasmid also significantly improved basal respiration, ATP production, and maximal respiration in *Pla2g6* KO N2a cells (Supplementary Fig. 6k, l). These results confirm that the disruption of ER-mitochondria integrity due to PLA2G6 deficiency leads to impaired mitochondrial morphology and function.

### PLA2G6 interacts with the IP3R1-GRP75-VDAC1 complex

**Fig. 3:**
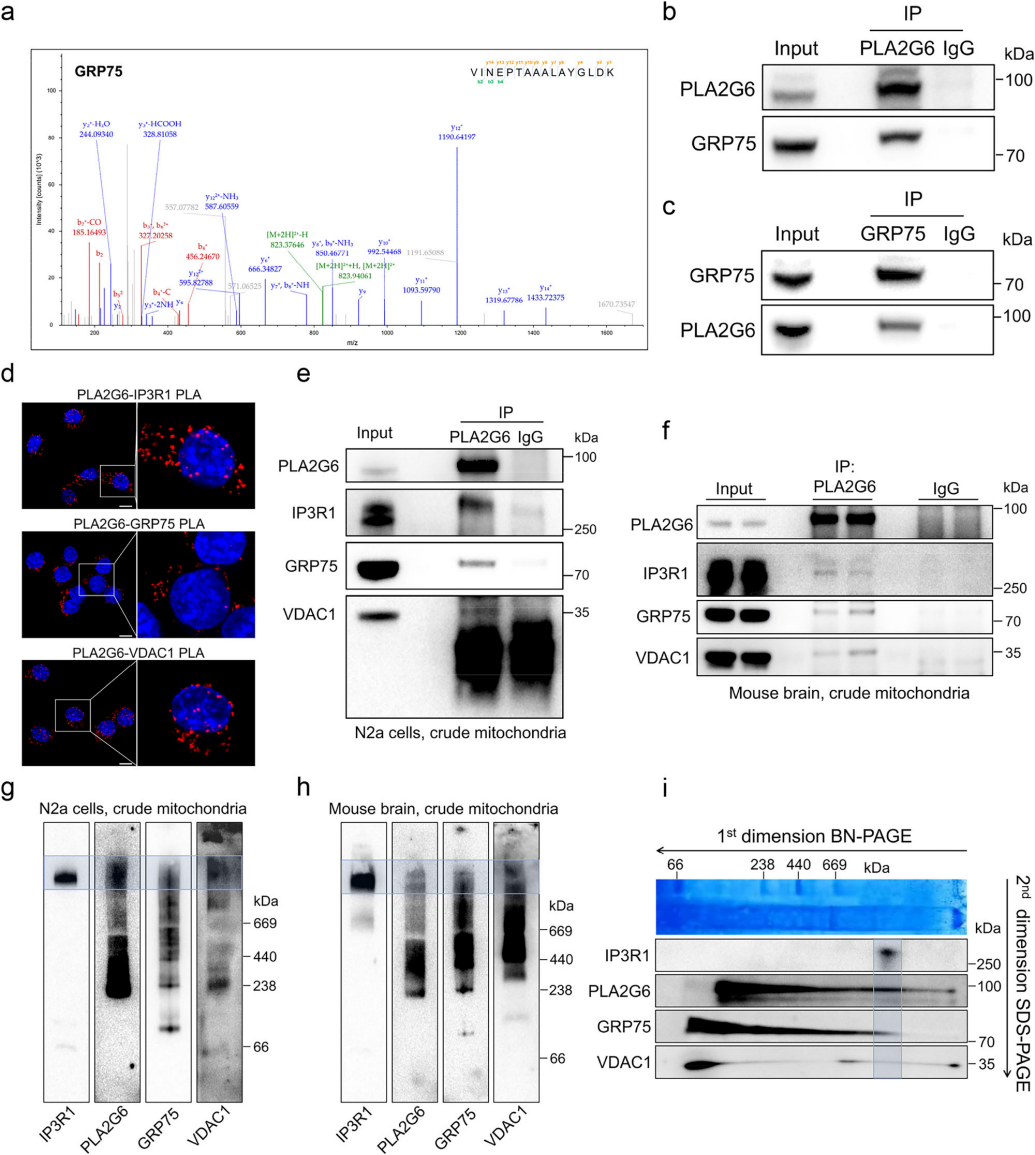
PLA2G6 is a component of the IP3R1-GRP75-VDAC1 complex at the MAM. **a** Mass spectrogram showing GRP75 identification. **b**, **c** Co-IP of GRP75 (**b**) and PLA2G6 (**c**) using anti-PLA2G6 or anti-GRP75 antibodies in whole lysates from normal N2a cells (*n* = 3 biologically independent experiments). **d** Representative confocal microscopy images of PLA (red signals) illustrating the close in situ association between PLA2G6 and IP3R1 (top), PLA2G6 and GRP75 (middle), and PLA2G6 and VDAC1 (bottom) in normal N2a cells (scale bars, 10 μm). White boxes indicate magnified regions on the right (*n* = 3 independent experiments). **e**, **f** Immunoblotting analysis of IP3R1, GRP75, and VDAC1 in PLA2G6 immunoprecipitates from crude mitochondrial fractions of normal N2a cells (**e**) (*n* = 3 independent experiments) and ventral midbrains of WT mice (**f**) (*n* = 4 mice). **g**, **h** Representative BN-PAGE and immunoblotting analysis of crude mitochondria isolated from normal N2a cells and ventral midbrains of WT mice (*n* = 3 biologically independent experiments). **i** Representative 2D BN-PAGE and immunoblotting analysis of crude mitochondria isolated from N2a cells (*n* = 3 biologically independent experiments). IP immunoprecipitation, PLA proximity ligation assay, BN-PAGE blue native-polyacrylamide gel electrophoresis.

To further explore the mechanism by which PLA2G6 regulates ER-mitochondria contacts, mass spectrometric (MS) analysis was conducted to determine whether PLA2G6 directly binds to ER-mitochondria tethering proteins. The analysis identified GRP75 as a potential PLA2G6-interacting protein within the MAM (Fig. 3a). This interaction was confirmed through co-immunoprecipitation (co-IP) experiments, where endogenous PLA2G6 and GRP75 were reciprocally pulled down, demonstrating their binding (Fig. 3b, c). Since our results of Fig. 1a–c showed that GRP75 coexists with PLA2G6 protein in the MAM and mitochondria, we isolated mitochondria and analyzed the submitochondrial localization of PLA2G6 and GRP75 to determine their precise location within mitochondria. When mitochondria remain intact, only proteins localized to the mitochondrial outer membrane (MOM), such as TOM20, are accessible to Protease K digestion. However, disrupting the MOM with a hypotonic swelling buffer exposes proteins of the mitochondrial inner membrane (MIM), like TIM23, allowing their subsequent degradation by the Protease. Only after complete membrane lysis with Triton X-100, which led to the digestion of all the mitochondrial proteins by Protease K, including matrix proteins (NDUFA9). Our results showed that most of the endogenous PLA2G6 is digested by Protease K in mitochondria treated with hypotonic swelling buffer, but all the endogenous GRP75 is digested by Protease K in mitochondria treated with Triton X-100 (Supplementary Fig. 7a). Furthermore, we found endogenous PLA2G6 co-immunoprecipitated with IP3R1 in the MAM fraction (Supplementary Fig. 7b). These results suggest that most of the PLA2G6 proteins coexist and interact with GRP75 in the MAM, but not in the submitochondrial compartments.

Given previous findings that GRP75 forms a complex with IP3R1 and VDAC1 in the MAM to maintain its integrity and function^39^, this study assessed whether PLA2G6 interacts with the IP3R1-GRP75-VDAC1 complex. An in situ proximity ligation assay (PLA), which detects protein interactions within a 40 nm range, was used to evaluate the proximity of PLA2G6 to the components of this tripartite complex. The assay confirmed colocalization between PLA2G6 and GRP75 (Fig. 3d, middle), as well as PLA2G6 with both IP3R1 and VDAC1 (Fig. 3d, top and bottom). These interactions were also observed in human brain samples (Supplementary Fig. 8a). To ensure specificity, control experiments using a single antibody or *Pla2g6* KO models yielded no PLA signals, ruling out non-specific binding (Supplementary Fig. 8b–h). These results indicate close spatial proximity of PLA2G6 with the IP3R1-GRP75-VDAC1 complex. To further confirm the physical interaction between PLA2G6 and the IP3R1-GRP75-VDAC1 complex, co-IP assays were performed on crude mitochondrial fractions (MAM + mitochondria) both in vitro and in vivo. Endogenous PLA2G6 co-immunoprecipitated with IP3R1, GRP75, and VDAC1 from the mitochondrial fractions of N2a cells (Fig. 3e). Similarly, co-IP analysis of crude mitochondrial fractions obtained from mouse ventral midbrains revealed that endogenous PLA2G6 can co-immunoprecipitate with IP3R1, GRP75, and VDAC1 proteins (Fig. 3f). Previous studies have reported that DJ-1 and GSK3β also interacted with IP3R1-GRP75-VDAC1 complex^23,31^, thus we performed co-IP experiment to determine whether PLA2G6 physically associated with DJ-1 and GSK3β. We found that endogenous PLA2G6 also co-immunoprecipitates with DJ-1 and GSK3β, but not with lysosomal protein LAMP1 (Supplementary Fig. 7c).

To evaluate whether PLA2G6 forms a macromolecular complex with IP3R1, GRP75, and VDAC1, blue native polyacrylamide gel electrophoresis (BN-PAGE) was conducted using crude mitochondrial fractions in both in vitro and in vivo conditions. Consistent with previous reports^31,39,40^, IP3R1, GRP75, and VDAC1 were detected as part of a high molecular weight complex (Fig. 3g, h). Notably, despite PLA2G6 existing at multiple molecular weights, a portion of it was also found in the same high molecular weight complex as IP3R1, GRP75, and VDAC1 (Fig. 3g, h). Furthermore, a two-dimensional (2D) BN-PAGE analysis of crude mitochondrial fractions from N2a cells confirmed the co-presence of PLA2G6, IP3R1, GRP75, and VDAC1 in the same macromolecular complex (Fig. 3i). Collectively, these results demonstrate that PLA2G6, along with IP3R1, GRP75, and VDAC1, forms a functional macromolecular complex within the MAM under physiological conditions.

### PLA2G6 deficiency disrupts the integrity of the IP3R1-GRP75-VDAC1 complex

**Fig. 4:**
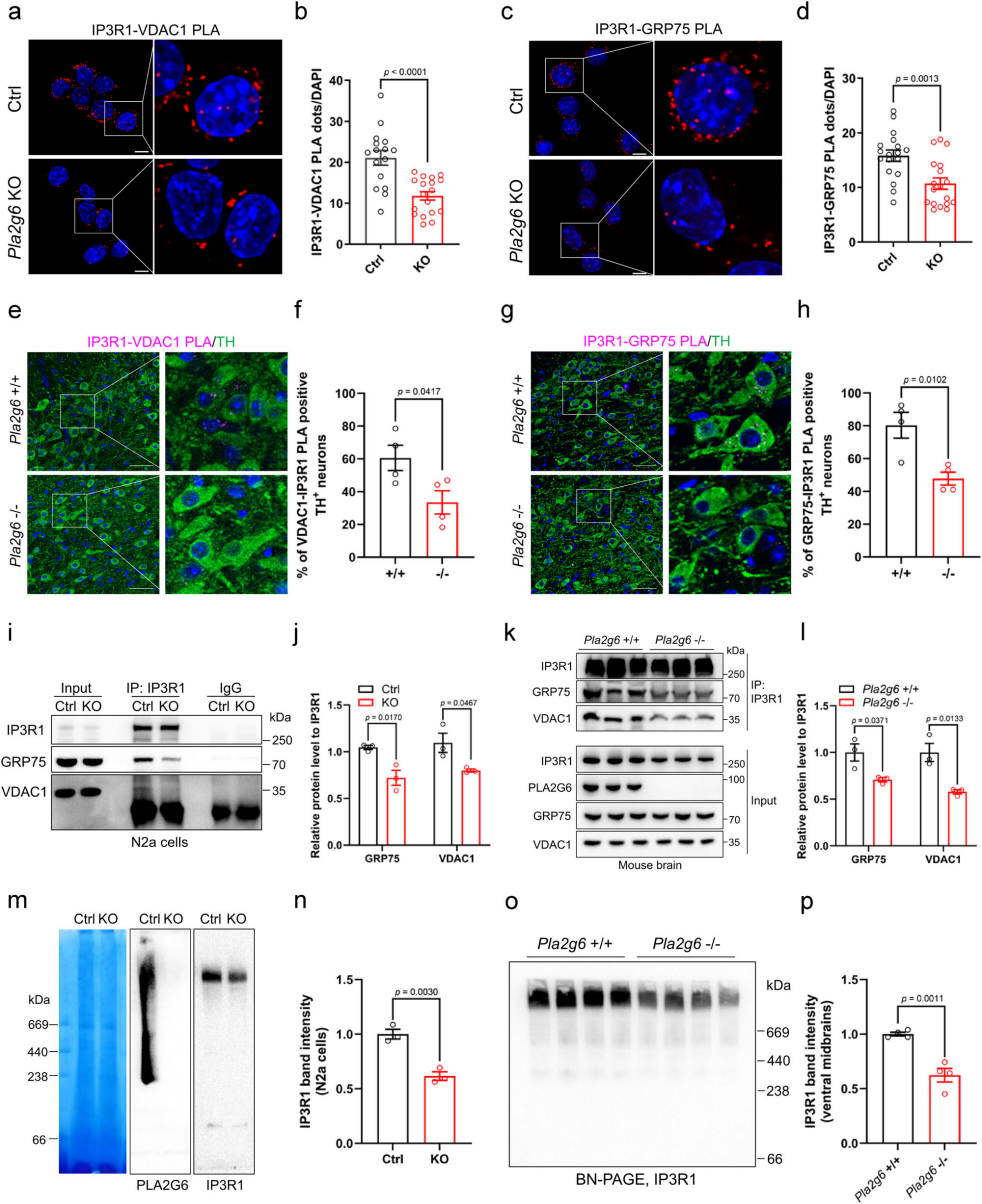
PLA2G6 deficiency disrupts the IP3R1-GRP75-VDAC1 complex. **a**, **b** The PLA analysis (red signals) and quantification of the IP3R1-VDAC1 interactions in control (*n* = 193 cells in 16 fields from three independent experiments) and *Pla2g6* KO N2a cells (*n* = 156 cells in 18 fields from three independent experiments). White boxes in the images are magnified on the right (scale bars, 10 μm). **c**, **d** The PLA analysis (red signals) and quantification of IP3R1-GRP75 interactions in control (*n* = 147 cells in 18 fields from three independent experiments) and *Pla2g6* KO N2a cells (*n* = 124 cells in 18 fields from three independent experiments). White boxes in the images are magnified on the right (scale bars, 10 μm). **e**–**h** The PLA analysis (magenta signals) and quantification of IP3R1-VDAC1 and IP3R1-GRP75 interactions in TH (green)-positive neurons from the substantia nigra of WT and *Pla2g6* KO mice (*n* = 4 mice/group). White boxes in the images are magnified on the right (scale bar, 50 μm). **i** Immunoprecipitation of IP3R1 from control and *Pla2g6* KO N2a cell lysates. **j** Quantitative analysis of IP3R1, GRP75, and VDAC1 in immunoprecipitates (*n* = 3 biologically independent experiments). **k** Immunoprecipitation of IP3R1 from lysates of ventral midbrains from WT and *Pla2g6* KO mice, followed by WB using IP3R1, GRP75, and VDAC1 antibodies. **l** Quantitative analysis of IP3R1, GRP75, and VDAC1 in immunoprecipitates (*n* = 3 mice/group). **m** BN-PAGE image showing the IP3R1 complex in crude mitochondria from control and *Pla2g6* KO N2a cells. **n** Quantitative analysis of the IP3R1 complex in crude mitochondria from control and *Pla2g6* KO N2a cells (*n* = 3 biologically independent experiments). **o** Representative BN-PAGE image showing the IP3R1 complex in crude mitochondria from WT and *Pla2g6* KO mice. **p** Quantitative analysis of the IP3R1 complex in crude mitochondria from WT and *Pla2g6* KO mice (*n* = 4 mice/group). Data are presented as means ± SEM. Two-tailed unpaired *t*-tests were used for statistical analysis (**b**, **d**, **f**, **h**, **j**, **l**, **n**, **p**). PLA proximity ligation assay, TH tyrosine hydroxylase, IP immunoprecipitation, BN-PAGE blue native-polyacrylamide gel electrophoresis.

Given the established association of PLA2G6 with the IP3R1-GRP75-VDAC1 complex in the MAM and the observed disruption of ER-mitochondria contacts in PLA2G6-deficient cells, further investigation was conducted to determine if PLA2G6 deficiency destabilizes this complex, thereby impairing ER-mitochondria interactions. PLA was initially performed in both control and *Pla2g6* KO N2a cells to assess interactions between IP3R1 and VDAC1, as well as IP3R1 and GRP75. Results indicated a significant reduction in PLA signal dots, corresponding to these interactions, in *Pla2g6* KO cells compared to controls (Fig. 4a–d). Similarly, a decrease in IP3R1-GRP75 and IP3R1-VDAC1 PLA signals was observed in TH-positive neurons from *Pla2g6* KO mice relative to WT mice (Fig. 4e–h). To ensure specificity, control experiments using *IP3R1* KO models yielded no PLA signals, ruling out non-specific binding (Supplementary Fig. 8i–k). Moreover, co-IP assays demonstrated a diminished co-precipitation of VDAC1 and GRP75 by IP3R1 in *Pla2g6* KO N2a cells compared to control cells (Fig. 4i, j). These results were mirrored in the ventral midbrains of WT and *Pla2g6* KO mice (Fig. 4k, l). Consistent with co-IP results, BN-PAGE of crude mitochondrial fractions from both N2a cells and ventral midbrain tissues revealed a reduction in the macromolecular complex recognized by IP3R1 in *Pla2g6* KO cells and mice (Fig. 4m–p). Collectively, these results confirm that *Pla2g6* KO disrupts the IP3R1-GRP75-VDAC1 complex, leading to impaired ER-mitochondria contacts.

### Expression of WT but not PD-associated PLA2G6 D331Y mutant rescues *Pla2g6* KO-induced MAM deficits

The homozygous c.991 G > T (p.D331Y) missense mutation in the *PLA2G6* gene has been implicated in early-onset autosomal recessive PD^41–43^. To assess whether the PLA2G6 D331Y mutant similarly disrupts MAM structure and function by impairing the IP3R1-GRP75-VDAC1 complex, CRISPR-resistant human WT PLA2G6 and the PLA2G6 D331Y mutant were re-expressed in *Pla2g6* KO N2a cells (Supplementary Fig. 9a). Initial co-IP analysis revealed that, despite comparable expression levels of WT PLA2G6 and the PLA2G6 D331Y mutant in *Pla2g6* KO N2a cells, re-expression of human WT PLA2G6 significantly enhanced its interaction with IP3R1 compared to the D331Y mutant (Supplementary Fig. 9b, c). Furthermore, WT PLA2G6 promoted the physical association of IP3R1 with GRP75 and VDAC1, whereas the PLA2G6 D331Y mutant failed to do so (Supplementary Fig. 9b, c).

Mitochondrial Ca^2+^ import and OCR were subsequently evaluated following the re-expression of WT and D331Y mutant PLA2G6 in *Pla2g6* KO N2a cells. Re-expression of WT PLA2G6 successfully restored mitochondrial Ca^2+^ import in *Pla2g6* KO N2a cells upon histamine stimulation (Supplementary Fig. 9d, e). Additionally, the reductions in basal respiration, ATP production, and maximal respiration observed in *Pla2g6* KO N2a cells were alleviated by WT PLA2G6 re-expression (Supplementary Fig. 9f, g), confirming its functional activity in mouse N2a cells. In contrast, the PLA2G6 D331Y mutant was unable to rescue these deficiencies (Supplementary Fig. 9f, g). Overall, these results demonstrate that the re-expression of WT PLA2G6, but not the D331Y mutant, mitigates the impairments in mitochondrial Ca^2+^ import and ATP production caused by *Pla2g6* KO.

### PLA2G6 ablation promotes the excessive degradation of IP3R1

**Fig. 5:**
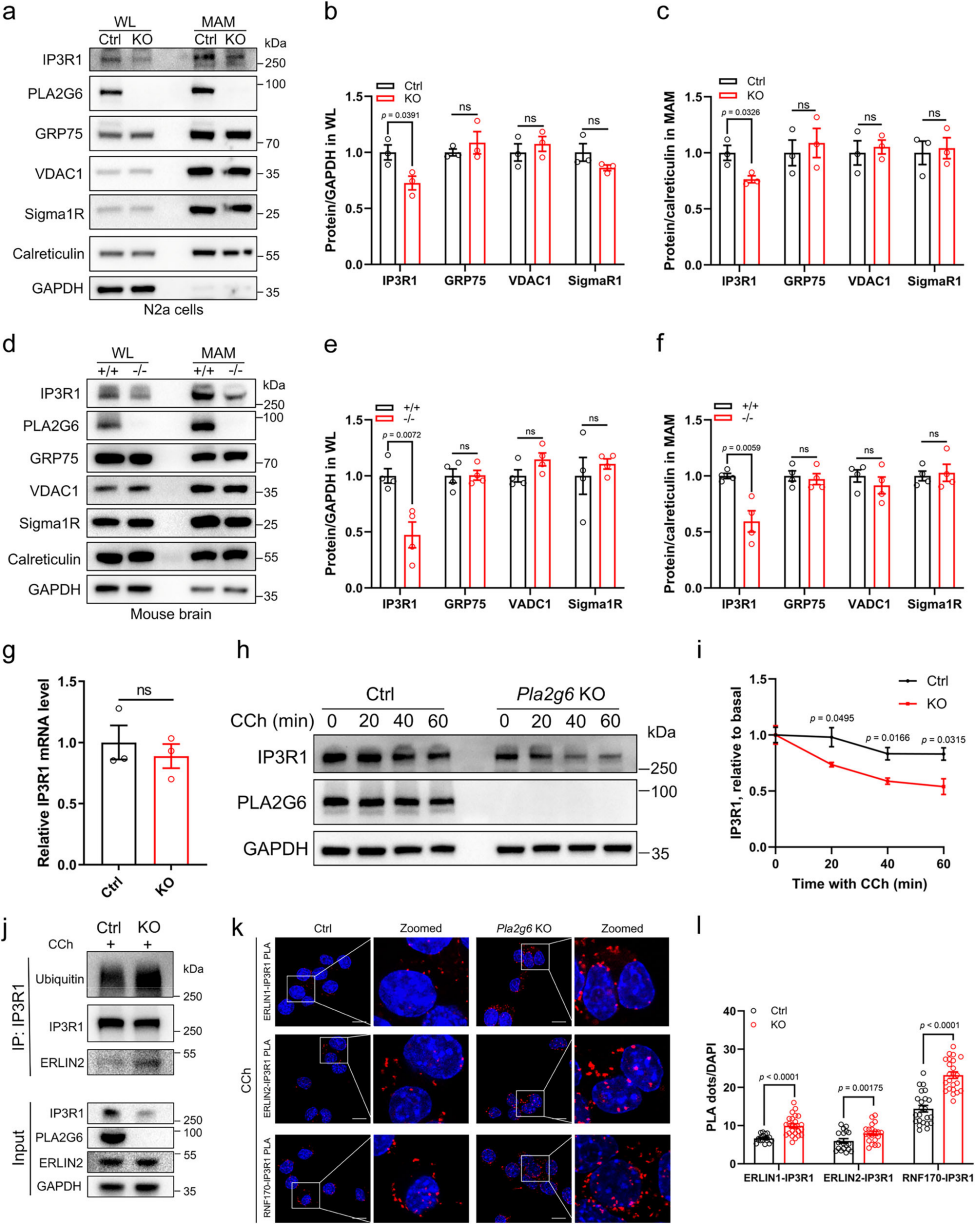
PLA2G6 deficiency promotes IP3R1 degradation. **a**–**c** Immunoblotting and quantitative analysis of MAM proteins IP3R1, GRP75, VDAC1, and Sigma1R in whole-cell lysates and MAM fractions from control and *Pla2g6* KO N2a cells (*n* = 3 biologically independent experiments). **d**–**f** Immunoblotting and quantitative analysis of MAM proteins IP3R1, GRP75, VDAC1, and Sigma1R in whole lysates and MAM fractions from ventral midbrains of WT and *Pla2g6* KO mice (*n* = 4 mice/group). **g** Quantitative analysis of IP3R1 mRNA expression in control and *Pla2g6* KO N2a cells (*n* = 3 biologically independent experiments). **h** Immunoblotting analysis of time-dependent degradation of IP3R1 following 10 μM CCh stimulation in control and *Pla2g6* KO N2a cells. **i** Quantitative analysis of changes in IP3R1 protein expression after CCh stimulation (*n* = 3 biologically independent experiments). **j** Immunoprecipitation of IP3R1 from control and *Pla2g6* KO N2a cells treated with 10 μM CCh for 20 min, followed by WB probing for IP3R1, ubiquitin, and ERLIN2 (*n* = 3 biologically independent experiments). **k** In situ PLA analysis (red signals) of ERLIN1-IP3R1, ERLIN2-IP3R1, and RNF170-IP3R1 interactions in control and *Pla2g6* KO N2a cells following CCh treatment. White boxes in the images are magnified on the right (scale bars, 10 μm). **l** Quantification of PLA dots in control (ERLIN1-IP3R1, *n* = 174 cells in 18 fields; ERLIN2-IP3R1, *n* = 131 cells in 18 fields; RNF170-IP3R1, *n* = 198 cells in 23 fields from three independent experiments) and *Pla2g6* KO N2a cells (ERLIN1-IP3R1, *n* = 143 cells in 24 fields; ERLIN2-IP3R1, *n* = 182 cells in 20 fields; RNF170-IP3R1, *n* = 176 cells in 23 fields from three independent experiments). Data are presented as means ± SEM. Two-tailed unpaired *t*-tests were used for statistical analysis (**b**, **c**, **e**–**g**, **i**, **l**). WL whole lysates, MAM mitochondria-associated ER membrane, ns not significant, CCh carbachol, PLA proximity ligation assay.

To further investigate whether PLA2G6 influences the expression of IP3R1-VDAC1 channels, WB analysis was performed on whole-cell lysates and MAM fractions from N2a cells and mouse ventral midbrains. A notable reduction in IP3R1 levels was observed in both whole-cell lysates and MAM fractions of *Pla2g6* KO N2a cells and mouse brains (Fig. 5a–f). However, no significant changes were detected in the levels of GRP75, VDAC1, and Sigma1R in either the whole-cell lysates or MAM fractions of *Pla2g6* KO cells and mice (Fig. 5a–f). Furthermore, to determine whether the phospholipase activity of the PLA2G6 protein is essential for IP3R1 stabilization, we re-expressed the WT PLA2G6 and catalytically impaired PLA2G6 (S519A) in *Pla2g6* KO N2a cells and showed no significant difference in IP3R1 protein expression levels between the WT group and S519A group (Supplementary Fig. 10a, b). This data suggests that the phospholipase activity of PLA2G6 is not essential for IP3R1 stabilization. Since previous studies indicated that the catalytic function of PLA2G6 is impaired by mutations associated with infantile neuroaxonal dystrophy (INAD), but not PD^44^, we assessed whether PD-associated mutants (D331Y, R741Q) and INAD-associated mutants (G517C, G638R) impact Ca²⁺ transfer and IP3R1 levels. Results demonstrated that re-expressing the D331Y mutant in KO cells showed no significant difference in Ca²⁺ transfer or IP3R1 levels compared to empty vector-transfected KO controls, with both being lower than KO cells expressing WT PLA2G6. In contrast, re-expression of INAD-associated mutants (G517C, G638R) or the PD-associated mutant (R741Q) upregulated Ca²⁺ transfer and IP3R1 levels to levels comparable to those observed in KO cells expressing WT PLA2G6 (Supplementary Fig. 10c–f). These findings are potentially attributable to the fact that mutations of G517C, G638R, or R741Q do not affect the ANK domain. This indicates that mutations at different sites within the PLA2G6 gene may lead to distinct pathogenic mechanisms.

In addition, to explore whether the regulation between PLA2G6 and IP3R1 proteins is unidirectional or reciprocal, we constructed stable *IP3R1* KO N2a cells and transiently overexpressed IP3R1-GFP in control cells. Our finding demonstrates that IP3R1 does not regulate PLA2G6 expression (Supplementary Fig. 10g–j). To elucidate the mechanisms underlying the downregulation of IP3R1 protein, mRNA levels of IP3R1 were assessed in control and *Pla2g6* KO N2a cells using quantitative real-time polymerase chain reaction (qRT-PCR). No significant differences in IP3R1 mRNA levels were detected (Fig. 5g), suggesting that the observed reduction in IP3R1 protein is likely due to post-translation regulation.

To investigate whether PLA2G6 deficiency leads to the downregulation of IP3R1 through an accelerated protein degradation pathway, carbachol (CCh), a widely used activator of IP3R1 that induces its activation and subsequent degradation, was administered at different time points to both control and *Pla2g6* KO N2a cells to assess the effect on IP3R1 homeostasis^45^. Immunoblotting analysis showed a more rapid degradation of IP3R1 in *Pla2g6* KO N2a cells compared to controls (Fig. 5h, i), indicating that PLA2G6 deficiency promotes accelerated IP3R1 degradation. Furthermore, basal IP3R1 protein levels were lower in *Pla2g6* KO cells under normal conditions (Fig. 5a, d, h), highlighting PLA2G6’s role in maintaining IP3R1 stability in resting cells. Therefore, we treated both control and *Pla2g6* KO N2a cells with cycloheximide (CHX), a protein synthesis inhibitor, at different time points to assess the degradation efficiency of IP3R1 protein under normal conditions. The result demonstrated that *Pla2g6* KO led to accelerated degradation of IP3R1 under normal conditions (Supplementary Fig. 10k, l). In addition, we used MG-132 and 3-MA to block proteasomal and lysosomal degradation pathways, respectively. The result showed that MG-132, but not 3-MA, restored the degradation of IP3R1 in *Pla2g6* KO cells under normal conditions, suggesting that the degradation of IP3R1 due to *Pla2g6* KO is through the proteasomal pathway (Supplementary Fig. 10m, n).

Previous studies have also indicated that activated IP3R1 undergoes degradation via the ubiquitin-proteasome pathway, facilitated by ER lipid raft-associated protein 1/2 (ERLIN1/2) and the E3 ubiquitin ligase Ring Finger Protein 170 (RNF170)^46–48^. To further elucidate the mechanism by which PLA2G6 deficiency enhances IP3R1 degradation, both control and *Pla2g6* KO N2a cells were stimulated with CCh for 20 min. A reduction in IP3R1 expression and increased ubiquitination of IP3R1 were observed in *Pla2g6* KO cells (Fig. 5j). Additionally, an enhanced interaction between IP3R1 and ERLIN2 was detected following PLA2G6 depletion (Fig. 5j). PLA assay was conducted to assess the physical association between IP3R1 and the ERLIN1/2-RNF170 complex in CCh-treated cells. Increased number of PLA signals for IP3R1-ERLIN1, IP3R1-ERLIN2, and IP3R1-RNF170 was observed in *Pla2g6* KO cells compared to controls (Fig. 5k, l). Similarly, the number of PLA signals for IP3R1-ERLIN1, IP3R1-ERLIN2, and IP3R1-RNF170 also increased in *Pla2g6* KO cells compared to controls under basal conditions (Supplementary Fig. 10o, p), suggesting that PLA2G6 deficiency promotes the binding of the ERLIN1/2-RNF170 complex to IP3R1 protein, thereby accelerating its degradation.

### Mutant *PLA2G6* leads to MAM dysfunction in the dopaminergic neurons derived from patients with PD

**Fig. 6:**
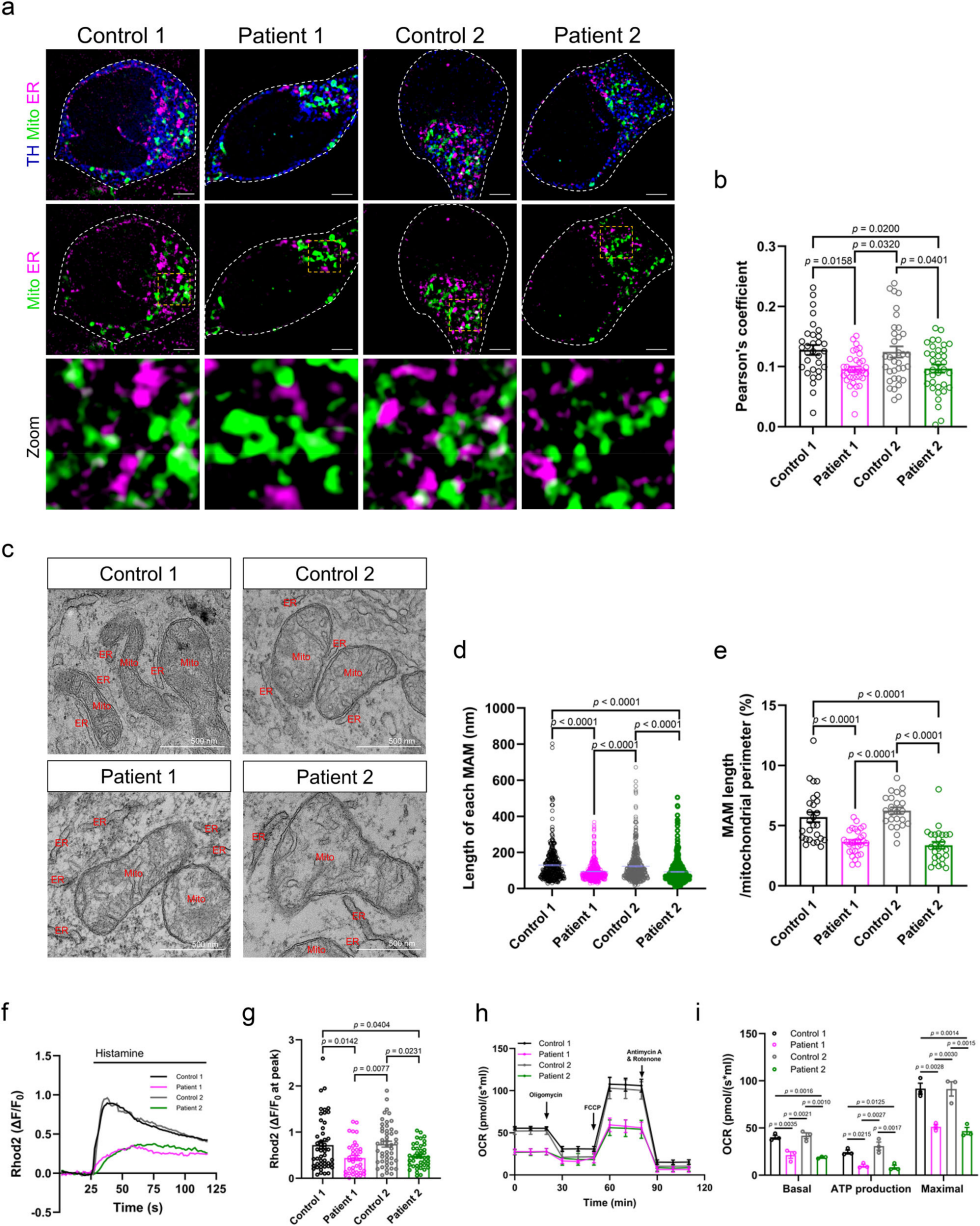
Dysregulated ER-mitochondria contacts in dopaminergic neurons derived from patients with PD carrying mutant PLA2G6. **a** HIS-SIM analysis of ER-mitochondria interactions. Cells were immunostained with TH (blue), TOM20 (green), and PDI (magenta) to mark dopaminergic neurons, mitochondria, and ER, respectively. The area within white dashed lines represents the dopaminergic neurons. Yellow dotted boxes indicate magnified regions below (scale bar, 10 μm). **b** Quantification of TOM20-PDI colocalization using Pearson’s coefficient (control 1, *n* = 31 neurons; patient 1, *n* = 34 neurons; control 2, *n* = 34 neurons; patient 2, *n* = 35 neurons from three independent experiments). **c** Representative TEM micrographs of dopaminergic neurons from healthy controls and patients (scale bar, 500 nm). **d**, **e** Quantification of MAM length (nm) and MAM length/mitochondrial perimeter (%) in dopaminergic neurons from controls and patients (control 1, *n* = 27 neurons and 573 mitochondria; patient 1, *n* = 29 neurons and 601 mitochondria; control 2, *n* = 24 neurons and 562 mitochondria; patient 2, *n* = 25 neurons and 575 mitochondria from three independent experiments). **f** Representative traces of mitochondrial Ca^2+^ after histamine (200 μM) stimulation in dopaminergic neurons from controls and patients, measured using Rhod2 dye. **g** Quantification of histamine-stimulated Ca^2+^ peak values (control 1, *n* = 50 neurons; patient 1, *n* = 38 neurons; control 2, *n* = 49 neurons; patient 2, *n* = 42 neurons from three independent experiments). **h** OCR measurements in dopaminergic neurons from controls and patients (*n* = 3 biologically independent experiments). **i** Quantification of basal OCR, ATP production, and maximal OCR in two million cells from each group (*n* = 3 biologically independent experiments). Data are presented as means ± SEM. One-way ANOVA with Tukey’s multiple comparison tests (**b**, **d**, **e**, **g**, **i**). TH tyrosine hydroxylase, Mito mitochondria, ER endoplasmic reticulum, MAM mitochondria-associated ER membrane, OCR oxygen consumption rate.

Given that loss-of-function mutations in the *PLA2G6* gene are linked to autosomal recessive PD^41,42^ and that PLA2G6 plays a pivotal role in regulating ER-mitochondria contacts, further investigation was conducted to assess these contacts, as well as mitochondrial Ca^2+^ import and ATP production, in dopaminergic neurons derived from induced pluripotent stem cells (iPSCs) of patients with PD. iPSCs from two patients with PD harboring *PLA2G6* mutations and two healthy controls were differentiated into dopaminergic neurons using established protocols (Supplementary Fig. 11a–c)^49,50^. Immunostaining of these neurons with TH, TOM20, and PDI as markers for dopaminergic neurons, mitochondria, and ER, respectively, followed by colocalization analysis using HIS-SIM, revealed a reduction in ER-mitochondria contacts in patient-derived neurons compared to controls (Fig. 6a, b). This observation was corroborated by TEM, which showed a decrease in both the length of individual MAMs and the MAM length-to-mitochondrial perimeter ratio in neurons from *PLA2G6*-mutant patients (Fig. 6c–e). Mitochondrial Ca^2+^ uptake was assessed using the indicator dye Rhod2, followed by histamine treatment (200 μM), revealing a significant reduction in Ca^2+^ import in *PLA2G6*-mutant neurons compared to controls (Fig. 6f, g). OCR measurements also indicated lower basal respiration, ATP production, and maximal respiration in patient-derived neurons relative to healthy controls (Fig. 6h, i). Notably, the application of the ER-mitochondria Linker successfully restored mitochondrial Ca^2+^ import, basal respiration, ATP production, and maximal respiration in *PLA2G6*-mutant neurons (Supplementary Fig. 12a–d). In addition, we also demonstrated a diminished co-precipitation of PLA2G6, VDAC1, and GRP75 by IP3R1 in *PLA2G6*-mutant neurons compared to controls (Supplementary Fig. 13a, b). BN-PAGE of crude mitochondrial fractions from dopaminergic neurons revealed a reduction in the macromolecular complex recognized by IP3R1 in *PLA2G6*-mutant neurons compared to controls (Supplementary Fig. 13c, d). These results closely parallel those observed in *Pla2g6* KO N2a cells and mouse brains, suggesting that loss-of-function mutations in *PLA2G6* contribute to PD pathogenesis by disrupting ER-mitochondria contacts, leading to impaired mitochondrial Ca^2+^ import and reduced ATP production.

## Discussion

**Fig. 7:**
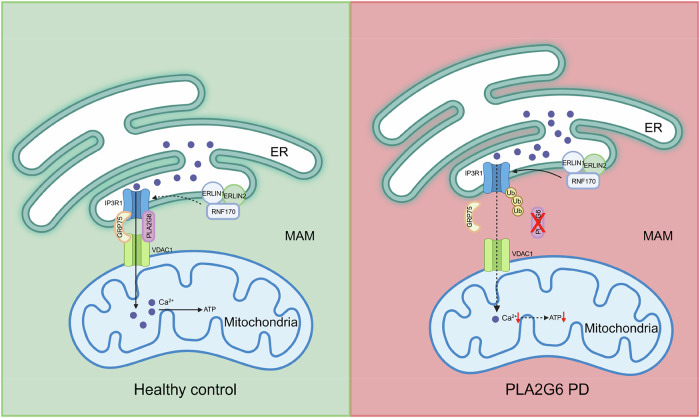
Loss of PLA2G6 disrupts ER-mitochondria contacts and Ca^2+^ transfer. Under normal conditions (left), PLA2G6 is enriched in the MAM and physically interacts with the IP3R1-GRP75-VDAC1 complex, thereby maintaining normal ER-mitochondria contacts and facilitating Ca^2+^ transfer. In contrast, in patients with Parkinson’s disease carrying *PLA2G6* mutations (right), the integrity of the IP3R1-GRP75-VDAC1 complex is compromised, leading to increased degradation of the IP3R1 protein by the E3 ubiquitin ligase RNF170. This disruption ultimately results in diminished ER-mitochondria contacts, reduced Ca^2+^ transfer, and decreased mitochondrial ATP production. (Created in BioRender. Lin, Z. (2026) https://BioRender.com/vd4xclu).

Collectively, our findings reveal a role for PLA2G6 in regulating MAM function. PLA2G6 forms a macromolecular complex with IP3R1, GRP75, and VDAC1, which is essential for maintaining the integrity and functionality of the MAM. PLA2G6 deficiency disrupts this complex, leading to impaired mitochondrial Ca^2+^ import and reduced ATP production (Fig. 7). These results provide critical insights into the dysfunction of ER-mitochondria communication in the pathogenesis of PLA2G6-related PD.

Our study extends the previously recognized physiological roles of PLA2G6. As a member of the phospholipase A2 family, PLA2G6 is involved in phospholipid hydrolysis, iron and ceramide metabolism, and the maintenance of ER and mitochondrial homeostasis^20,21,51,52^. Notably, a previous study has demonstrated that neuronal overexpression of MAM protein C19orf12 effectively rescued the heat sensitivity, motor disability, and bang sensitivity phenotypes observed in *PLA2G6*^−/−^ flies. Furthermore, enhancement of MAM integrity ameliorates the bang-sensitive seizure phenotype of *PLA2G6*^−/−^ flies^51^, while they did not recognize the important role of PLA2G6 in the MAM. These data suggest that there may be a connection between the PLA2G6 protein and MAM. In addition, cytoplasmic Ca^2+^ levels are diminished in dopaminergic neurons carrying the PLA2G6 D331Y mutation, or in cells where PLA2G6 activity is suppressed^53,54^. However, the role of PLA2G6 in regulating Ca^2+^ transfer from the ER to mitochondria has remained unclear. Moreover, mutations in *PLA2G6* are implicated in a spectrum of disorders collectively termed PLA2G6-associated neurodegeneration (PLAN), including infantile neuroaxonal dystrophy (INAD), atypical neuroaxonal dystrophy (ANAD), and PD. Previous studies have shown that neural progenitor cells and dopaminergic neurons derived from patients with infantile neuroaxonal dystrophy (INAD) harboring the PLA2G6 R70X mutation exhibit mitochondrial defects^38^, while neurons from patients with PD harboring PLA2G6 D331Y mutation display mitochondrial dysfunction and ER stress^54^. However, the specific role of ER-mitochondria contacts in these conditions remains unexplored. Our data suggest that PLA2G6 plays a direct role in mitochondrial ATP production by regulating Ca^2+^ transfer from the ER to mitochondria *via* MAM contact sites. These contact sites provide a platform for Ca^2+^ influx into mitochondria, facilitating Ca^2+^-dependent mitochondrial functions such as reactive oxygen species generation, motility, and ATP production^32–34,55,56^. Notably, PKD2 channels and ryanodine receptors are also ER Ca^2+^ efflux channels, but unlike the IP3R1 agonist histamine, both PKD2 agonist triptolide and ryanodine receptors agonist caffeine do not cause differences in mitochondrial Ca^2+^ uptake between the control and *Pla2g6* KO groups, suggesting that PLA2G6 regulation of ER-mitochondria Ca^2+^ transport is specifically dependent on IP3R1 channels. Furthermore, the use of a MAM Linker rescued the deficits in mitochondrial ATP production and membrane potential in *Pla2g6* KO cells, indicating that MAM dysfunction is upstream of the observed mitochondrial impairments. Notably, the mitochondrial fusion protein MFN2 and mitochondrial fission protein Fis1 can regulate the ER-mitochondria contacts^9,57^, and some studies indicate that mitochondrial elongation reduced the MAM formation^58,59^. Therefore, abnormal mitochondrial morphology may further affect its contacts with the ER.

Abnormal ER-mitochondria contacts have been implicated in various neurodegenerative diseases, including PD, Alzheimer’s disease (AD), and amyotrophic lateral sclerosis (ALS). For example, both wild-type and PD-associated mutant α-synuclein have been shown to disrupt the ER-mitochondria association and reduce mitochondrial Ca^2+^ import by interfering with the vesicle-associated membrane protein-associated protein B (VAPB)-protein tyrosine phosphatase interacting protein 51 (PTPIP51) complex^22^. Similarly, DJ-1 deficiency, much like PLA2G6 deletion, disrupts the IP3R-GRP75-VDAC1 complex and impairs ER-mitochondria Ca^2+^ transfer, suggesting a potential functional or physical link between DJ-1 and PLA2G6 within the MAM^23^. Parkin, another key protein, has also been localized to the MAM, where it regulates MAM integrity and function through the ubiquitination of Mitofusin 2 (MFN2)^24,60^. Furthermore, increased ER-mitochondria communication has been observed in AD^61,62^, while ALS neurons with pathogenic C9orf72 expansions exhibit reduced ER-mitochondria crosstalk^63^. These findings, along with our results, emphasize that proper ER-mitochondria contacts are vital for neuronal function, and MAM dysfunction may represent a common pathological mechanism in neurodegeneration. Notably, our study demonstrated that restoring disrupted MAM can rescue mitochondrial Ca^2+^ import and ATP production deficits, highlighting the therapeutic potential of targeting ER-mitochondria associations in neurodegenerative diseases.

The PLA2G6 protein has been reported to exhibit diverse subcellular localization, including the mitochondria, ER, Golgi, nucleus, and plasma membrane, depending on cell type and stimuli^64–67^. Consistent with prior research^20^, a portion of PLA2G6 was detected in both mitochondria and the ER in our results. Notably, PLA2G6 was also localized at ER-mitochondria contacts in N2a cells, mouse ventral midbrain, and human brains under physiological conditions. Thus, our study identifies a previously unrecognized subcellular localization of PLA2G6. ER-mitochondria tethering primarily relies on four complexes: IP3R1-GRP75-VDAC1, MFN2-MFN1/2, BAP31-Fis1/Tom40, and VAPB-PTPIP51^12,13,39,68–70^. Our findings demonstrated that PLA2G6 forms a macromolecular complex with IP3R1, GRP75, and VDAC1 in the MAM, a complex that likely has both structural and functional significance. The study revealed that PLA2G6 deficiency disrupts the physical interaction of the IP3R1-GRP75-VDAC1 complex both in vitro and in vivo. Given that this complex plays a critical role in ER-mitochondria tethering and Ca^2+^ transfer^23,39^, the observed disruption helps explain why loss-of-function mutations in *PLA2G6* result in decreased ER-mitochondria contacts and impaired mitochondrial Ca^2+^ import. Additionally, D331Y mutations in PLA2G6 diminished interactions between IP3R1 and both PLA2G6 and GRP75, failing to rescue the Ca^2+^ import and ATP production deficits induced by PLA2G6 deficiency. Dopaminergic neurons derived from patients with PD carrying the D331Y mutation also exhibited reduced ER-mitochondria contacts, mitochondrial Ca^2+^ import, and ATP production. This mutation, prevalent in the Chinese population, has been shown to reduce phospholipase activity by approximately 70% in vitro^42^. Our findings provide insights into the pathogenic effects of the D331Y mutation in PLA2G6. Moreover, PLA2G6 appears to specifically affect IP3R1 homeostasis in the MAM, without altering the levels of VDAC1, GRP75, or Sigma1R. Both IP3R1 and Sigma1R are critical for maintaining the dynamic stability of ER-mitochondria contacts, and their proper expression is essential for cellular function^71–73^. IP3R1 is located on the ER membrane and serves as the primary Ca^2+^ release channel from the ER to mitochondria. The Ca^2+^ released by IP3R1 enters the mitochondrial matrix through VDAC1 on the mitochondrial outer membrane and mitochondrial calcium uniporter on the mitochondrial inner membrane, thereby mediating mitochondrial Ca^2+^ signaling and function. Thus, the degradation of IP3R1 directly affects the mitochondrial Ca^2+^ import. The activity of key dehydrogenases in the tricarboxylic acid cycle is Ca^2+^-dependent, and reduced mitochondrial Ca^2+^ uptake consequently diminishes ATP production, thereby impairing both action potential propagation and neurotransmitter release of neurons^74^.

Notably, the D331Y mutant of PLA2G6 decreased the IP3R1 level and mitochondrial Ca^2+^ transfer, but the R741Q mutant did not, which may be attributed to the fact that the R741Q mutant does not affect the ANK domain. Interestingly, prior studies demonstrated that the R741Q is common in the Indian and Middle East cohorts, whereas the homozygous or compound heterozygous D331Y is the most common mutation in the Asian cohort. Moreover, milder clinical and neuroimaging phenotypes were noted with the D331Y mutation and a relatively severe phenotype in R741Q^75^. Furthermore, the pathogenic mechanisms underlying PD-associated PLA2G6 mutations exhibit significant differences across distinct loci. For example, the D331Y mutation primarily affects endoplasmic reticulum and mitochondrial morphology and function^54^, whereas the R747W mutation increases cellular susceptibility to ferroptosis^76^.

Previous studies have shown that the binding of IP3 to activated IP3R1 recruits the ERLIN1/2-E3 ubiquitin ligase RNF170 complex, promoting the ubiquitination and degradation of IP3R1^45–48^. Consistent with this, PLA2G6 deficiency accelerated the degradation of activated IP3R1 induced by CCh, an IP3 inducer^45^, and enhanced the interaction of the ERLIN1/2-RNF170 complex with IP3R1, further increasing its ubiquitination. These results suggest that PLA2G6 plays a protective role in preventing the excessive degradation of IP3R1 by limiting the binding of the ERLIN1/2-RNF170 complex.

In conclusion, our study demonstrates that the PD-associated protein PLA2G6 forms a macromolecular complex with IP3R1, GRP75, and VDAC1, maintaining the integrity and function of ER-mitochondria contacts. This work provides insights into the pathogenic mechanisms underlying PLA2G6-related Parkinson’s disease.

## Methods

### Plasmids and reagents

The plasmid pCMV CEPIA4mt was obtained from MiaoLingBio (P18478), and pCMV R-CEPIA1er was sourced from Addgene (#58216). The OMM-mRFP-ER and OMM-GFP-ER plasmids, generously provided by Xing-Guo Liu, served as MAM Linkers^36,77^. The pTagBFP-Mito, pEGFP-Mito, pEGFP-ER, pDsRed-ER, pDsRed-PLA2G6, LAMP1-pDsRed, and IP3R1-pEGFP plasmids were purchased from Youbio Biological Technology. pcDNA-PLA2G6 (human)-3 × Flag, pcDNA-D331Y PLA2G6 (human)-3 × Flag, pcDNA-G517C PLA2G6 (human)-3 × Flag, pcDNA-G638R PLA2G6 (human)-3 × Flag, pcDNA-R741Q PLA2G6 (human)-3 × Flag, pcDNA-S519A PLA2G6 (human)-3 × Flag, pcDNA-ΔPNPLA, and pcDNA-ΔANK plasmids were acquired from WZ Biosciences. Additional reagents used in the study include histamine (Selleck, S3968), Oligomycin (Sigma, O4876), FCCP (Sigma, C2920), rotenone (Sigma, R8875), Antimycin A (Sigma, A8674), carbachol (Selleck, S4359), triptolide (Selleck, S3604), caffeine (Chengdu Must Bio-Technology, A0470), RR (Selleck, E2993), MCU-i4 (Selleck, S9842), CHX (Selleck, S7418), MG-132 (Selleck, S2619), and 3-MA (Selleck, S2767). The α-Syn PFFs were kindly gifted by Zhen-Tao Zhang from Renmin Hospital of Wuhan University.

### Cell cultures, transfection, and treatments

Mouse neuroblastoma N2a cells were obtained from the Cell Bank of the Chinese Academy of Sciences (Beijing, China, catalog number SCSP-5035). U2OS cells were obtained from Procell Life Science & Technology (Wuhan, China, catalog number CL-0236). The α-Syn-HEK293 cells were donated by Zhen-Tao Zhang from Renmin Hospital of Wuhan University. These cells were cultured in DMEM (Gibco, 11960044), supplemented with 10% fetal bovine serum (Gibco, 10099141C) and 1% penicillin/streptomycin (Gibco, 15140122), and maintained at 37 °C in a 5% CO₂ environment.

Transfection of N2a, U2OS cells with plasmids was performed using Lipofectamine 3000 (Invitrogen, L3000075) following the manufacturer’s protocol. N2a cells were stimulated with 10 μM carbachol or 100 μg/mL CHX for various time intervals.

The α-Syn-HEK293 cells were transduced with PFFs according to previous methods^78^. Briefly, cells were seeded in 24-well plates. First, 5 μg of PFFs was diluted in Opti-MEM (Gibco, 11058021) to achieve a final volume of 100 μL. In a separate tube, 96 μL of Opti-MEM was combined with 4 μL of Lipofectamine 2000 (Invitrogen, 11668030). The two solutions were then mixed and incubated at room temperature for 20 min. Finally, the resulting mixture was added to the culture medium. α-Syn-HEK293 cells were treated with 5 μM RR or 2 μM MCU-i4 to inhibit the MCU.

Primary cortical neurons were prepared from E18 embryonic mice and cultured in Neurobasal media (Gibco, 21103049) supplemented with B-27 (Gibco, 17504044), glutamax (Gibco, 35050061), and 1% penicillin/streptomycin (Gibco, 15140122), and maintained at 37 °C in a 5% CO₂ environment. Primary neurons were subjected to 1 μg of PFFs, 5 μM RR, or 2 μM MCU-i4 on days in vitro (DIV) 7 and used for immunofluorescence or WB experiments on DIV 14.

### Dopaminergic neuron differentiation and virus infection

Primary skin fibroblasts were obtained from one 30-year-old male and one 41-year-old female patients with *PLA2G6-*mutant PD, as well as two male healthy controls (aged 32 and 35 years). This was done with written informed consent (approval No. 2015-048). The fibroblasts were cultured in DMEM (Gibco, 11960044) supplemented with 10% fetal bovine serum (Gibco, 10099141 C) and 1% penicillin/streptomycin (Gibco, 15140122) at 37 °C in 5% CO_2_. Patient 1 carried compound heterozygous variants: c.991 G > T (p.D331Y) and c.1631 T > C (p.M544T). Patient 2 had compound heterozygous variants: c.991 G > T (p.D331Y) and c.1915delG (p.A639Qfs*27). Both patients were previously reported^79^. Healthy control fibroblasts were kindly provided by Professor Zhi-Ying Wu from the Second Affiliated Hospital of Zhejiang University School of Medicine. Genomic DNA was extracted from fibroblasts, and mutations were validated through Sanger sequencing, with primers listed in Supplementary Table 2. Informed consent was obtained from all participants.

Fibroblasts were reprogrammed into iPSCs using the CytoTune^TM^-iPS 2.0 Sendai Reprogramming Kit (Invitrogen, A16517). Briefly, fibroblasts were thawed and cultured for 4 days in 24-well plates using a fibroblast culture medium. Fibroblasts were then transduced with Sendai viral particles expressing SOX2, c-MYC, OCT4, and KLF4, according to the manufacturer’s instructions. By day 21, iPSC colonies were ready for transfer, and undifferentiated iPSCs were manually picked and expanded on fresh culture dishes. The selected iPSCs were passaged every 4 days until no residual Sendai virus remained. The pluripotency of the iPSCs was confirmed by staining for markers TRA-1-60 (Millipore, MAB4360, 1:100), SOX2 (Invitrogen, 14-9811-82, 1:100), SSEA4 (CST, 4755S, 1:100), and OCT3/4 (Santa Cruz, sc-5279, 1:50).

Dopaminergic neuron differentiation from iPSCs followed previously established protocols^49,50^. Briefly, healthy control and PLA2G6-PD iPSCs were dissociated using EDTA (Invitrogen, 15575-020) and seeded on Matrigel-coated (Corning, 354277) 10 cm dishes. The differentiation protocol included exposure to 10 μM β-mercaptoethanol (Invitrogen, 21985-023) from days 1–7, 200 nM LDN193189 (Selleck, S2618) from days 1–11, and 10 μM SB431542 (Tocris, 1614) from days 1–7. Cells were also exposed to 100 ng/mL human sonic hedgehog (Peprotech, 100-45) from days 2–9, 2 μM purmorphamine (TargetMol, T1810) from days 2–9, 100 ng/mL FGF8 (Peprotech, 100-25) from days 2–9, and 1 μM CHIR99021 (Sigma, SML1046) from days 3–14. On day 9, 120 μM quercetin (Sigma, Q4951) was added to eliminate undifferentiated iPSCs. On day 15, cells were dissociated using Accutase (Gibco, A1110501) and re-plated on 6 cm dishes coated with Poly-L-ornithine (Sigma, P4957), Fibronectin (Sigma, F0895), and Laminin (Sigma, L2020), using differentiation medium supplemented with 200 μM ascorbic acid (Sigma, A4403), 20 ng/mL BDNF (Peprotech, 450-02), 20 ng/mL GDNF (Peprotech, 450-10), 500 μM dbcAMP (Sigma, D0627), and 1 ng/mL TGF-β3 (Peprotech, 100-36E). For the characterization of human dopaminergic neurons, the differentiated cells were stained with the following specific markers: TH (dopaminergic neuronal marker, CST, 58844 or Sigma, MAB318, 1:500), MAP2 (neuronal marker, Abcam, ab32454, 1:500), TUJ1 (neuronal marker, Sigma, T8578, 1:500), FOXA2 (ventral midbrain neuronal marker, abnova, H00003170, 1:100), NURR1 (dopaminergic neuronal marker, Proteintech, 10975-2-AP, 1:100), Girk2 (dopaminergic neuronal marker, Proteintech, 21647-1-AP, 1:100). Dopaminergic neurons were infected with lentivirus expressing OMM-GFP-ER or an empty vector (WZ Biosciences) at a multiplicity of infection of 20, and cells were analyzed 72 h post-infection.

### Generation knockout (KO) cell lines

KO cells were generated using the CRISPR-Cas9 gene-editing system. The mouse *Pla2g6* guide RNA sequence (WZ Biosciences): 5′ CTCAAGTGAACGTGTCCGGG 3′, the mouse *IP3R1* guide RNA sequence (IGE Biotechnology): 5′ CCGTCAACTGTAATACAAGC 3′, the human *PLA2G6* guide RNA sequence (IGE Biotechnology): 5′ GAGGGGGCTGGCTCCGTAACG 3′. The sequence was cloned into the PX459 vector, which carries the pSpCas9. This construct was then transfected into N2a cells using Lipofectamine 3000 (Invitrogen, L3000075), following the manufacturer’s instructions. After 36 h, cells were subjected to puromycin selection for an additional 36 h. Surviving cells were diluted into single-cell populations in 96-well plates, and gene knockout was confirmed via immunoblotting.

### Animals and animal care

*Pla2g6* KO C57BL/6Smoc mice (Cat. No. NM-KO-200413) were obtained from Shanghai Model Organisms Center, Inc. Mice were housed in a temperature- and humidity-controlled environment, under a 12-h light/dark cycle, with access to water and food. Three-month-old *Pla2g6* KO male mice and age-matched WT male littermates were used for experiments. Genotyping was performed using PCR, following the protocol provided by Shanghai Model Organisms Center, Inc. All experimental procedures were approved and overseen by the Institutional Ethics Committee of the Second Affiliated Hospital of Zhejiang University School of Medicine (approval No. 2022-0240).

### Human brain samples

Human brain samples were collected from one 54-year-old male and one 70-year-old female patients who underwent deep brain stimulation. They provided written informed consent. Experiments involving human brain samples were conducted in accordance with the Declaration of Helsinki and were approved by the Institutional Ethics Committee of the Second Affiliated Hospital of Zhejiang University School of Medicine (approval No. 2024-0134).

### MAM fractionation

MAM fractions were isolated from N2a cells and mouse ventral midbrains using a previously established protocol^23,80^. Cells or tissues were homogenized in lysis buffer (225 mM mannitol, 75 mM sucrose, and 30 mM Tris-Cl, pH 7.4) using a Dounce homogenizer. Lysates were first centrifuged at 600 × *g* for 5 min to remove unbroken cells and nuclei. The resulting supernatant was centrifuged at 7000 × *g* and then at 10,000 × *g* for 10 min to separate the crude mitochondrial pellet from the ER-containing supernatant. To purify the ER, the supernatant was centrifuged at 20,000 × *g* for 30 min and then at 100,000 × *g* for 1 h. The crude mitochondrial pellet was resuspended in mitochondrial suspension buffer (MRB, 250 mM mannitol, 5 mM HEPES pH 7.4, and 0.5 mM EGTA), and the suspension was layered onto a Percoll medium (225 mM mannitol, 25 mM HEPES pH 7.4, 1 mM EGTA, and 30% (v/v) Percoll). After centrifugation at 95,000 × *g* for 30 min, the MAM fraction was separated from the upper third of the centrifuge tube, while the mitochondrial fraction was isolated from the lower third. The mitochondrial fraction was centrifuged at 6300 × *g* for 10 min to obtain a pure mitochondrial pellet. The MAM fraction was then diluted in MRB and subjected to centrifugation at 6300 × *g* for 10 min. Subsequently, the supernatant was centrifuged at 100,000 × *g* for 1 h to isolate the MAM pellet.

### Submitochondrial localization analysis

N2a cells were harvested from six 15-cm dishes, and then isolated mitochondria. The mitochondrial pellets were resuspended in homogenization buffer (20 mM HEPES/KOH, pH 7.4, 220 mM mannitol, 70 mM sucrose, 1 mM EDTA, 0.5% BSA) supplemented with 1 mM PMSF and Protease inhibitor cocktail, then divided for different treatments: two aliquots in homogenization buffer (control), two in hypotonic buffer (10 mM HEPES/KOH, pH 7.4, 1 mM EDTA), and two in buffer containing 0.5% Triton ×-100 (incubated on ice for 10 min). One aliquot from each group was treated with 66.7 μg/ml Protease K on ice for 20 min, while the other remained untreated. Samples were precipitated with 300 μl of 30% (w/v) TCA on ice for 10 min, centrifuged at 18,000 × *g* for 10 min at 4 °C, washed with 100% ethanol, and dissolved in 100 μl sample buffer (60 mM Tris-HCl, pH 6.8, 7% glycerol, 2% 2-mercaptoethanol, 2% SDS, 0.02% bromophenol blue). After boiling at 95 °C for 10 min, 10 μl of each sample was subjected to immunoblotting.

### Immunofluorescent imaging

To determine the subcellular localization of PLA2G6, U2OS cells were seeded on the confocal dish (Cellvis, D35-20-1.5H) and transfected with pDsRed2-Mito, pEGFP-ER, and pTagBFP-PLA2G6 plasmids at 37 °C for 24 h. Super-resolution imaging was conducted using high intelligent and sensitive structured illumination microscopy (HIS-SIM) with a 100 × 1.5 NA oil immersion objective for three-dimensional analysis. Image processing is done using Wiener Reconstruction. The specific parameter information is as follows: defocus elimination 5, notch filter 10, 3D Wiener parameter 0.5. The 3D reconstruction utilized Imaris (Bitplane).

To assess the mitochondria-ER, mitochondria-lysosome, and ER-lysosome colocalization in U2OS cells, the cells were seeded on the confocal dish (cellvis, D35-20-1.5H) and transfected with fluorescent plasmids of organelles for 24 h. Dopaminergic neurons were grown on poly-D-lysine (PDL; Beyotime, ST508)-coated glass coverslips, followed by fixation, permeabilization, and blocking. Cells were incubated overnight at 4 °C with primary antibodies against TOM20 (CST, 42406S, 1:200), PDI (Abcam, ab2792, 1:100), and TH (Abcam, ab76442, 1:100). Secondary antibodies conjugated with Alexa Fluor (Invitrogen) were applied for one hour at room temperature the following day. 3D super-resolution images were captured by HIS-SIM and processed using Wiener Reconstruction (defocus elimination 5, notch filter 10, 3D Wiener parameter 0.5). Then we use Imaris software (Bitplane) to segment mitochondria and ER by Surface Creation and Filament Diameter, respectively. Finally, Pearson’s Correlation Coefficient was used to quantify the co-localization between ER and mitochondria.

For assessing the specificity of the Linker, N2a cells were cultured on glass slides and transfected with OMM-mRFP-ER, pTagBFP-Mito, and pEGFP-ER plasmids for 24 h. Super-resolution images were captured using HIS-SIM.

For assessing the intensity of pS129, the α-Syn-HEK293 or primary neurons were fixed with 4% PFA for 20 min, and then permeabilized with 1% TX-100 for 15 min at room temperature. After washing, cells were incubated overnight at 4 °C with primary antibodies against pS129 (Biolegend, 825701). Secondary antibodies conjugated with Alexa Fluor (Invitrogen) were applied for one hour at room temperature the following day. Images were captured by Leica TCS SP8 confocal microscope Leica TCS SP8 confocal microscopy and analyzed using ImageJ.

### Immuno-electron microscopy

Cells and mouse brains were fixed with 2% paraformaldehyde and 0.1% glutaraldehyde for 15 min at room temperature, followed by permeabilization with 0.1% saponin for 40 min and blocking with 0.1% BSA for 1 h at room temperature. Afterward, cells were incubated overnight at 4 °C with primary antibody PLA2G6 (Proteintech, 22030-1-AP, 1:50) or TH (CST, 58844, 1:100). Following PBS washes, cells were treated with a FluroNanogold-labeled anti-rabbit secondary antibody for 1 h at room temperature. Subsequent fixation was performed with 2.5% glutaraldehyde, followed by silver enhancement. Ultrathin sections were obtained using an ultramicrotome after 48 h of polymerization at 60 °C and examined via TEM (Tecnai G2 Spirit 120 kV).

### WB

Protein extraction from cells and mouse ventral midbrains was carried out using RIPA lysis buffer (Beyotime, P0013B) supplemented with a protease inhibitor cocktail (MCE, HY-K0010) on ice for 30 min. After centrifugation at 18407 × *g* for 10 min, the supernatant was collected, and protein concentration was quantified using the BCA Assay Kit (Invitrogen, 23227). Samples were mixed with loading buffer and boiled for 8 min before loading 20 μg of denatured protein onto 4–20% SDS-PAGE gels and transferring to PVDF membranes. The membranes were blocked with 5% milk for 1 h at room temperature, followed by overnight incubation with primary antibodies at 4 °C. The next day, membranes were treated with horseradish peroxidase-conjugated secondary antibodies. Immunoblotting signals were detected using a chemiluminescence reagent (Invitrogen, 32106) and quantified via ImageJ. The antibodies used for immunoblotting included PLA2G6 (Abcam, ab259950, 1:1000), GRP75 (Santa Cruz, sc-133137, 1:500), VDAC1 (Proteintech, 55259-1-AP, 1:1000), Sigma1R (CST, 61994, 1:1000), COX IV (ABclonal, A11631, 1:1000), Calreticulin (ABclonal, A20986, 1:1000), IP3R1 (ABclonal, A21471, 1:1000), MFN2 (Abcam, ab124773, 1:1000), TOM40 (ABclonal, A3213, 1:1000), TOM20 (CST, 42406S, 1:1000), TIM23 (Proteintech, 11123-1-AP, 1:1000), ubiquitin (Santa Cruz, sc-8017, 1:500), ERLIN2 (ABclonal, A0781, 1:1000), NDUFA9 (ABclonal, A3196, 1:1000), pS129 (CST, 23706, 1:1000), GSK3β (ABclonal, A2081, 1:1000), DJ-1 (Proteintech, 68915-6-Ig, 1:1000), GFP (HUABIO, ET1607-31, 1:2000), LAMP1 (Santa Cruz, sc-20011, 1:500) and GAPDH (Proteintech, 60004-1-Ig, 1:5000).

### Co-immunoprecipitation (Co-IP)

For Co-IP assays, proteins were extracted from cells and mouse ventral midbrains using 1% NP-40 lysis buffer (Beyotime, P0013F) supplemented with a protease inhibitor cocktail (MCE, HY-K0010) on ice for 30 min. Primary antibodies or IgG were incubated with 25 μL of protein G magnetic beads (Invitrogen, 10004D) at room temperature for 1 h. The protein lysates were then incubated with the antibody-bound magnetic beads overnight at 4 °C. The beads were washed four times with PBS the following day. Pulled-down proteins were eluted by boiling in 1× SDS-PAGE sample buffer and analyzed by WB. Antibodies used for IP included: PLA2G6 (Proteintech, 22030-1-AP, 1 μg), GRP75 (Santa Cruz, sc-133137, 1 μg), and IP3R1 (Santa Cruz, sc-271197, 1 μg). The precipitated proteins were in-gel digested by adding 1% trypsin and incubating at 37 °C overnight to peptides.

### Mass spectrometry (MS) analysis

For the IP experiment, PLA2G6 antibody (Proteintech, 22030-1-AP, 1 μg) was added to N2a cell lysates, with IgG serving as a negative control. Liquid chromatography-tandem mass spectrometry (LC-MS/MS) analysis was subsequently conducted by Hangzhou Cosmos Wisdom Biotech Co., Ltd. (IP, *n* = 2; IgG, *n* = 2 from 2 biologically independent experiments). The brief experimental protocols are as follows: the precipitated proteins were in-gel digested with 2 μg trypsin in 50 mM NH4HCO3 at 37 °C overnight to peptides. After digestion, peptides were extracted from the gel piece with 50% acetonitrile/0.1% formic acid, dried, and resuspended in 0.1% formic acid. The extracted peptides were dried in SpeedVacuum concentrator (Thermo Scientific) and resuspended in 0.1% formic acid for LC-MS/MS analysis.

Peptides were analyzed by LC-MS/MS using an EASY nLC-1200 system (Thermo Scientific) at a constant flow rate of 400 nl/min with the following gradient program: 2–7% mobile phase B (0.1% formic acid in 80% acetonitrile) over 1 min, 7–35% B over 35 min, 35–55% B over 9 min, 55–100% B over 7 min, and held at 100% B for 8 min. Eluates were analyzed using a Q Exactive HF-X mass spectrometer (Thermo Scientific) with an electrospray voltage of 2.0 kV; full MS scans were acquired at a resolution of 60,000, the top 20 most abundant precursor ions were fragmented by higher-energy collisional dissociation with a normalized collision energy of 27, MS/MS scans were acquired at a resolution of 30,000, and dynamic exclusion was set to 20 s.

Raw data were processed using Proteome Discoverer software (v2.4, Thermo Scientific) with database searches against the Swiss-Prot mouse protein database (release 2022_01). Trypsin/P was used as the cleavage enzyme with up to 2 missed cleavages; precursor ion mass tolerance was set to 10 ppm and fragment ion mass tolerance to 0.01 Da. Carbamidomethylation of cysteine was specified as a fixed modification, while oxidation of methionine and N-terminal acetylation were set as variable modifications. Only high-confidence peptides were retained for protein identification. Candidate proteins need to fulfill the following conditions: unique peptides ≥ 2, IP abundances/IgG abundances ≥ 10. Candidate proteins have all been listed in the Source Data, and PLA2G6, GRP75, and VDAC1 have been marked in red.

### Proximity ligation assay (PLA)

PLA was performed using Duolink® reagents (Sigma, DUO92008) to assess proteins located within <40 nm proximity. Briefly, cells or ventral midbrain sections were fixed, permeabilized, blocked, and incubated overnight at 4 °C with paired primary antibodies (Supplementary Table 1). After three washes, cells and sections were treated with oligonucleotide-conjugated secondary antibodies (anti-rabbit PLUS and anti-mouse MINUS). Ventral midbrain sections were incubated with the TH primary antibody (Abcam, ab76442, 1:400) following oligonucleotide-conjugated secondary antibody treatment. On Day 3, the sections were incubated with the corresponding secondary antibody (Invitrogen). A negative control was included for all PLA experiments. Images were captured using a Leica TCS SP8 confocal microscope, and PLA signal dots were quantified via ImageJ.

### Blue native-polyacrylamide gel electrophoresis (BN-PAGE)

Crude mitochondria were isolated from cells and ventral midbrains and lysed in 1% NP-40 on ice for 30 min. Supernatants were collected following centrifugation at 18407 × *g* for 10 min, and protein concentrations were determined with the BCA Assay Kit (Invitrogen, 23227). For the first dimension, 30 μg of protein mixed with 0.25% G-250 was loaded onto a 3–12% Bis-Tris gel (Invitrogen, BN1001BOX) and transferred to PVDF membranes. The gel was excised for the second dimension, reduced with 1 × NuPage reducing agent in 1 × NuPage LDS sample buffer for 15 min, and cysteine alkylation was performed with N,N-Dimethylacrylamide for 15 min at room temperature. The reaction was quenched with 20% ethanol in 1 × LDS and 0.1 × reducing agent for 15 min, after which the gel strip was transferred onto a 4–20% Tris-Glycine gel (Invitrogen, EC6026BOX) for immunoblotting analysis.

### TEM

N2a cells and dopaminergic neurons were fixed overnight at 4 °C in 2.5% glutaraldehyde, followed by refixation with 1% osmium tetroxide for 1 h, staining with 2% uranyl acetate for 30 min, and ethanol-gradient dehydration. Samples were embedded in 100% acetone for two hours and sectioned into 90 nm ultrathin slices. Sections were subsequently stained with 4% uranyl acetate and 1% lead citrate, and the ultrastructure of N2a cells was analyzed via TEM (Tecnai G2 Spirit 120 kV). The term “MAM length” refers to the measurement of the outer mitochondrial membrane specifically at the region where it contacts the ER membrane. And the criteria of ER-mitochondria contacts are defined as: the parallel membrane alignment ≤ 30 nm. The term “ratio of MAM length to mitochondrial perimeter” refers to the ratio of the total length of all MAMs to the sum of all mitochondrial perimeters in a single cell. To quantify the MAM lengths and mitochondrial perimeters, we first use ImageJ to convert the pixels of the image into lengths according to the scale bar. Then use the ImageJ manual tracing of the mitochondrial outer membrane to measure the length of MAM and the perimeter of the mitochondria. Measurements were carried out by investigators blinded to the experimental conditions.

### FIB-SEM

N2a cells were fixed in 2.5% glutaraldehyde for 24 h, followed by washing with 0.1 M phosphate buffer. Cells were then scraped and collected in 0.2% BSA/0.1 M phosphate buffer, and centrifuged at 845 × *g*. Post-fixation was performed with 2% OsO_4_ and 3% potassium ferricyanide in 0.1 M phosphate buffer for 1 h on ice. After rinsing with distilled water, cells were incubated in 1% thiocarbohydrazide for 20 min, followed by 30-min incubation with 1% aqueous uranyl acetate at room temperature. Samples were rinsed with distilled water, dehydrated in a graded ethanol series, and embedded in EPON 812 resin.

Resin blocks containing leaf tissue were meticulously trimmed using a trimmer. These blocks were mounted on a 90° stub and coated with platinum for 300 s using an ion-sputtering apparatus. To target specific areas, SEM equipped with an ultramicrotome was employed, facilitating simultaneous trimming of the resin blocks and imaging of the samples. Once the target area was identified, resin blocks were transferred to a 45° pre-tilted SEM stub and coated with platinum for 300 s. Imaging was performed with a dual beam SEM (FIB Helios G3 UC) in serial surface view mode with a 10 nm slice thickness at 30 kV and 0.79 nA. Each surface was imaged with a 2 kV acceleration voltage and 0.2 nA current in backscatter electron mode using an in-column backscatter electron detector (ICD). During milling, the sample stage was tilted at 7°, and for imaging, the tilt angle was adjusted to 45°. Images were captured at a resolution of 4096 × 3536 pixels with a dwell time of 2 μs per pixel.

The term “MAM surface” specifically denotes the region of the outer mitochondrial membrane that directly contacts the ER membrane. And the criteria of ER-mitochondria contacts are defined as: the parallel membrane alignment ≤ 30 nm. The term “MAM surface-to-mitochondrial surface ratio” refers to the proportion between the total contact surface of a single mitochondrial outer membrane with the ER membrane and the surface of that mitochondrion. The FIB-SEM slices stack is loaded by the Amira software. Then we run the Align Slices tool to correct the spatial misalignment between consecutive slices and run the Box Filter to reduce the noise of images. We segmented images by manual tracing of the mitochondrial outer membrane and ER membrane to generate surface reconstructions. To quantify the ER-mitochondria contacts, we first convert length (nm) to pixels according to voxel size (2.4847 × 2.4847 × 10 nm) and use the Arithmetic module to create a mask of overlapping proximity regions. Finally, we use Material Statistics to quantify the surface area of ER-mitochondria contacts (MAM surface) and the mitochondria surface. Measurements were carried out by investigators blinded to the experimental conditions.

### Subcellular Ca^2+^ monitoring

For Ca^2+^ monitoring, N2a cells were transfected with pCMV CEPIA4mt or pCMV R-CEPIA1er to assess mitochondrial or ER luminal Ca^2+^ levels. Dopaminergic neurons were stained with Rhod2 (Invitrogen, R1244) for mitochondrial Ca^2+^ level measurement, according to the manufacturer’s protocol. Following transfection or staining, cells were placed in Ca^2+^-free Hank’s buffered salt solution (HBSS) supplemented with 20 mM HEPES. Cells were stimulated with 200 μM histamine, 200 nM triptolide, or 5 mM caffeine, and imaged at 1.27-s intervals using an inverted confocal laser scanning microscope (ZEISS LSM 900) with a 40 × objective. Image analysis was performed with ImageJ using the time series analyzer V3 plugin. F_0_ values were calculated by averaging 20 frames prior to stimulation, and ΔF was defined as F_max_-F_0_.

### Oxygen consumption rate (OCR) measure

To measure the OCR, two million cells were added to each chamber of the Oxygraph-2k (OROBOROS Instruments). Sequential injections of the ATP synthase inhibitor Oligomycin (2.5 μM), the uncoupling reagent FCCP (2 μM), and a combination of rotenone (0.5 μM) and antimycin A (2.5 μM) were administered to block complexes I and III. These tests were utilized to determine various parameters of mitochondrial function, including basal respiration, ATP production, and maximal respiration. The basal respiration was calculated as the difference between the basal OCR (recorded before the addition of any modulators) and the non-mitochondrial OCR (recorded after the addition of antimycin A and rotenone). The ATP production was determined as the difference between basal respiration and the OCR recorded after the addition of oligomycin. The maximal respiration was calculated as the difference between the maximal OCR (recorded after the optimal concentration of FCCP addition) and the non-mitochondrial OCR.

### Mitochondrial membrane potential

Mitochondrial membrane potential was assessed in N2a cells using TMRM (Invitrogen, T668). Living cells were incubated with 100 nM TMRM dye for 30 min at 37 °C, followed by washing with PBS. The fluorescence of these living cells was measured using an inverted confocal laser scanning microscope (ZEISS LSM 900) with a 63 × objective.

### Lipid peroxidation

The levels of lipid peroxidation were measured using the BODIPY 581/591 C11 reagent (Invitrogen, D3861) following the manufacturer’s instructions. Briefly, The N2a cells were seeded in six-well plates and treated with RR or MCU-i4 for 24 h. They were then washed with PBS twice and incubated with 5 μM BODIPY 581/591 C11 reagent for 30 min at 37 °C. Cells were harvested and washed twice with PBS and then resuspended in 500 μL of PBS. 10,000 single cells were acquired from each group and measured by flow cytometer (Beckman CytoFLEX) equipped with a 488 nm laser. Data were analyzed using the FlowJo software.

### RNA isolation and qRT-PCR

Total RNA was isolated from N2a cells using TRIzol reagent (Takara, 9109) in accordance with the manufacturer’s instructions. Complementary deoxyribonucleic acid (cDNA) was synthesized using the 5 × Evo M-MLV RT Premix (Accurate Biology, AG11706). The qRT-PCR assay was then conducted using the 2 × SYBR Green Premix Pro Taq HS qPCR Kit (Accurate Biology, AG11701) on the StepOnePlus™ Real-Time PCR System (Invitrogen). The primers were purchased from Sangon Biotech and listed in Supplementary Table 2.

### Statistical analysis

All data presented were obtained from distinct samples, as specified in the figure legends, with no data excluded. Results are expressed as mean ± SEM for at least *n* = 3. Group differences were analyzed using a two-tailed unpaired Student’s *t*-test, while comparisons among multiple groups were assessed using one-way ANOVA with Tukey’s multiple comparison tests via Prism 9.5.1 software (GraphPad). A *p*-value of ≤ 0.05 was established as the threshold for statistical significance.

## Supplementary information

Supplementary Information

Description of Additional Supplementary Files

Supplementary Movie 1

Supplementary Movie 2

Supplementary Movie 3

Transparent Peer Review file

## Source data

Source data

## Data Availability

The protein mass spectrometry data have been deposited in ProteomeXchange under accession number PXD057620. The DNA sequencing data have been deposited in GSA for human under accession number HRA009235. The source data of Figs. 1–7 and Supplementary Figs. 1–13 are provided as a Source Data file. All other data and details of protocol are available from the corresponding author upon request. Source data are provided with this paper.
