# Supplementary material for: Parkinson’s disease-associated PLA2G6 protects IP3R1 protein to control ER-mitochondria tethering and Ca2+ transfer
Source: Nat Commun. 2026 Mar 19;17:5338. doi: 10.1038/s41467-026-70752-1 (PMC13272647; doi:10.1038/s41467-026-70752-1)
Supplement: Supplementary file 1 — Supplementary Information [file 41467_2026_70752_MOESM1_ESM.pdf]

Supplementary Information for

Parkinson's disease-associated PLA2G6 protects IP3R1 protein to control ER-mitochondria tethering and Ca^{2+} transfer

Zhi-Hao Lin *et al.*

*Corresponding author: Bao-Rong Zhang, brzhang@zju.edu.cn
; Jia-Li Pu, jialipu@zju.edu.cn

This PDF file includes:

Supplementary Figs. 1 to 13
Supplementary Tables 1 and 2

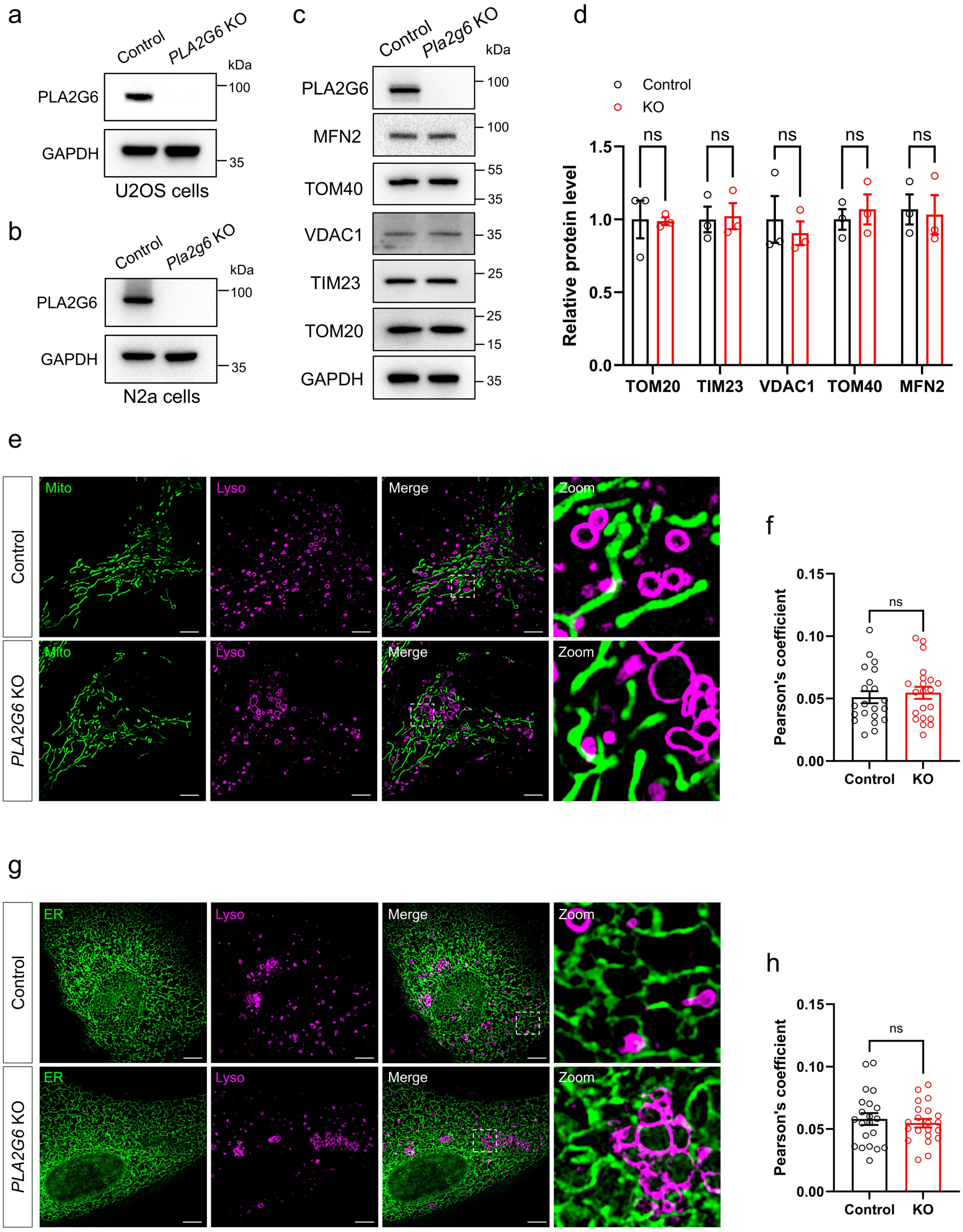

Supplementary Fig. 1 PLA2G6 deficiency does not alter mitochondria-lysosome and ER-lysosome contacts. (a, b) A stable *PLA2G6* KO U2OS (a) and a stable *Pla2g6* KO N2a (b) monoclonal cell line were established using the CRISPR/Cas9 method. (c) WB analyzed control and *Pla2g6* KO N2a cell lysates with antibodies against PLA2G6, MFN2, TOM40, VDAC1, TIM23, TOM20, and GAPDH. (d) Quantitative analysis of protein expression in control and *Pla2g6* KO N2a cell lysates. Data were normalized to GAPDH (n = 3 biologically independent experiments). (e) HIS-SIM analysis of mitochondria (green)-lysosome (magenta) interactions in control

and *PLA2G6* KO U2OS cells. White dotted boxes in the images are magnified on the right (scale bar, 5 μm). (f) Quantification of mitochondria-lysosome colocalization using Pearson's coefficient (control, n = 21 cells; *PLA2G6* KO, n = 21 from three independent experiments). (g) HIS-SIM analysis of ER (green)-lysosome (magenta) interactions in control and *PLA2G6* KO U2OS cells. White dotted boxes in the images are magnified on the right (h). Quantification of ER-lysosome colocalization using Pearson's coefficient (control, n = 21 cells; *PLA2G6* KO, n = 21 from three independent experiments). Data are presented as means ± SEM. Two-tailed unpaired *t*-tests were used for statistical analysis (d, f, h). Abbreviations: ns, not significant; Mito, mitochondria; Lyso, lysosome; ER, endoplasmic reticulum; MFN2, mitofusin 2; TOM40, translocase of outer mitochondrial membrane 40; VDAC1, voltage dependent anion channel 1; TIM23, translocase of inner mitochondrial membrane 23; TOM20, translocase of outer mitochondrial membrane 20.

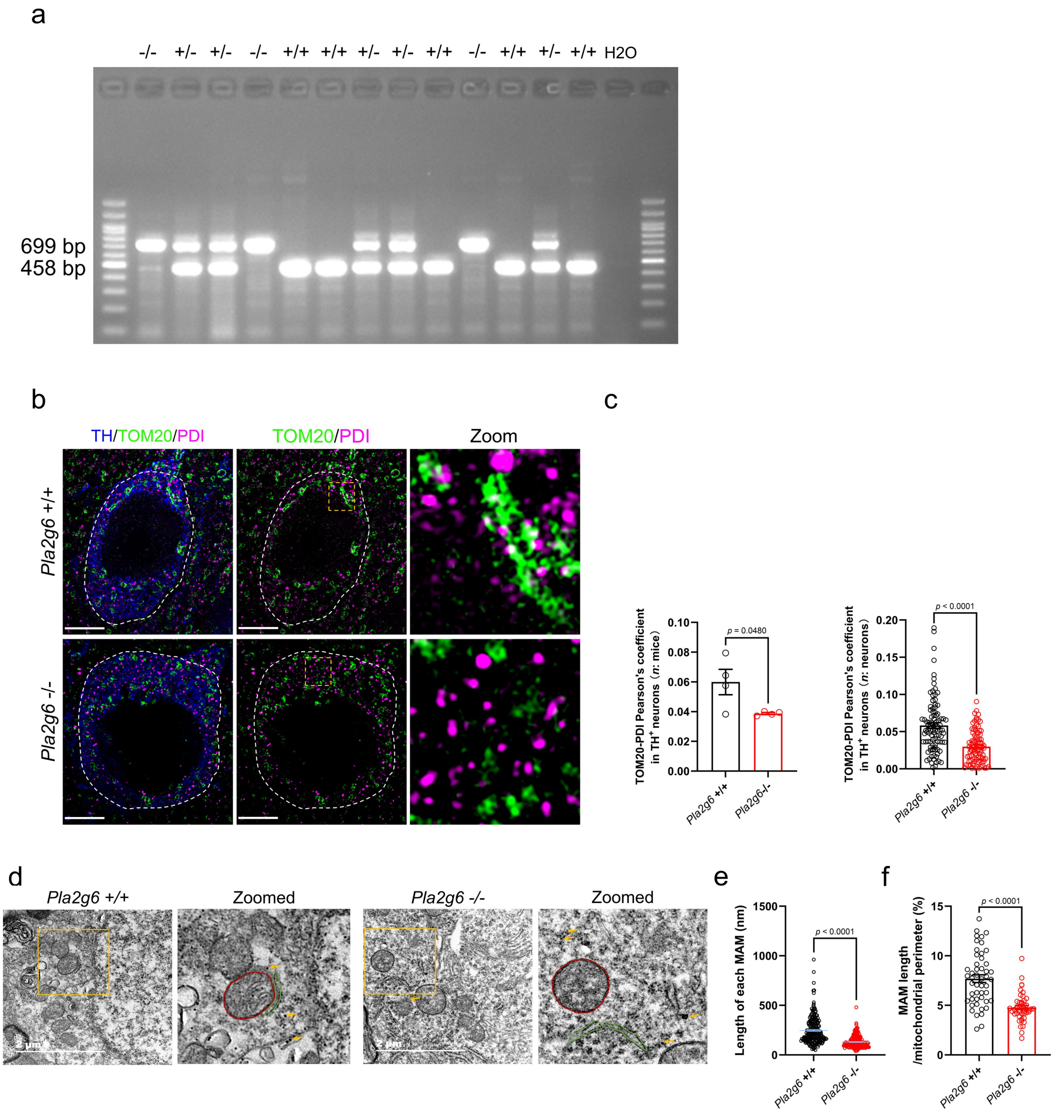

Supplementary Fig. 2 ***Pla2g6*** **KO decreases ER-mitochondria contacts in mice.** (a) The KO and WT mice were determined by a PCR experiment. The KO mice show only a 699 bp band, whereas the WT mice show only a 458 bp band. (b) Analysis of ER-mitochondria interaction by HIS-SIM. Cells were immunostained using TH (blue), TOM20 (green), and PDI (magenta) as marked for dopaminergic neurons, mitochondria, and ER, respectively. The area within white dashed lines represents the dopaminergic neurons. The yellow dotted boxes in the images were magnified on the right (scale bar, 10 μm). (c) Quantification of TOM20-PDI colocalization in dopaminergic neurons of WT (n = 4 mice, n = 96 neurons) and KO mice (n = 4 mice, n = 90 neurons) through Pearson's coefficient. (d) Representative immune electron microscopy images of dopaminergic neurons from WT and *Pla2g6* KO mice. The

yellow arrowheads indicate colloidal gold of TH. The yellow boxes are magnified on the right. The areas of red and green lines represent mitochondria and endoplasmic reticulum, respectively (scale bar, 2 μm). (e, f) Quantification of MAM length (nm) and MAM length/mitochondrial perimeter (%) in neurons from WT and *Pla2g6* KO mice (WT, n = 49 fields and 329 mitochondria; KO, n = 46 fields and 311 mitochondria from three mice per group). Data are presented as means ± SEM. Two-tailed unpaired *t*-tests were used for statistical analysis (c, e, f). Abbreviations: TH, tyrosine hydroxylase; TOM20, translocase of outer mitochondrial membrane 20; PDI, protein disulfide isomerase.

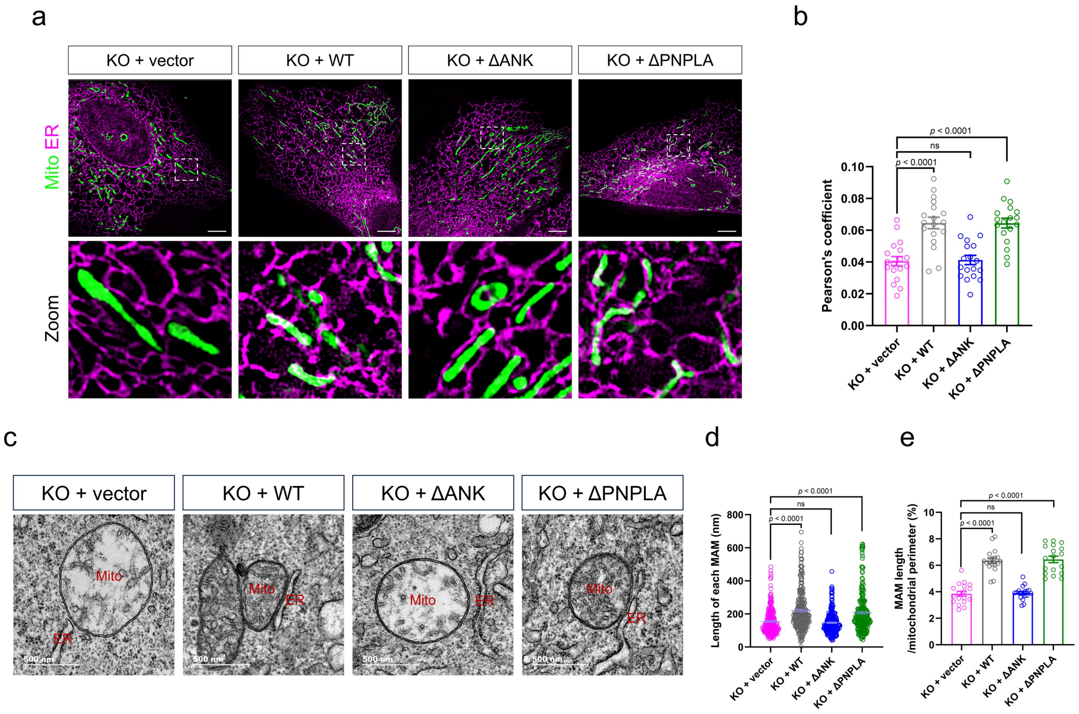

Supplementary Fig. 3 The ankyrin repeat (ANK) domain of PLA2G6 is essential for MAM formation. (A) ΔANK and ΔPNPLA indicate ANK (aa120–403) and PNPLA (aa481–665) truncated mutants, respectively. Representative HIS-SIM images of interactions between mitochondria (green) and ER (magenta) in the KO + vector, KO + WT, KO + ΔANK, and KO + ΔPNPLA groups. White dotted boxes in the images are magnified below (scale bar, 5 μm). (b) Quantification of ER-mitochondria colocalization using Pearson's coefficient (n = 18 cells per group, from three independent experiments). (c) Representative TEM micrographs of KO + vector, KO + WT, KO + ΔANK, and KO + ΔPNPLA groups (scale bar, 500 nm) (d, e, f) Determination of length of each MAM (nm) and MAM length/mitochondrial perimeter (%) in each group (KO + vector group, n = 18 cells and 357 mitochondria; KO + WT group, n = 18 cells and 369 mitochondria; KO + ΔANK group, n = 18 cells and 389 mitochondria; KO + ΔPNPLA group, n = 18 cells and 352 mitochondria from three independent experiments). Data are means ± SEM. One-way ANOVA with Tukey's multiple comparison tests was used for statistical analysis (b, d, e) . Abbreviations: ns, not significant; Mito, mitochondria; ER, endoplasmic reticulum; ANK, ankyrin repeat; PNPLA, patatin-like phospholipase.

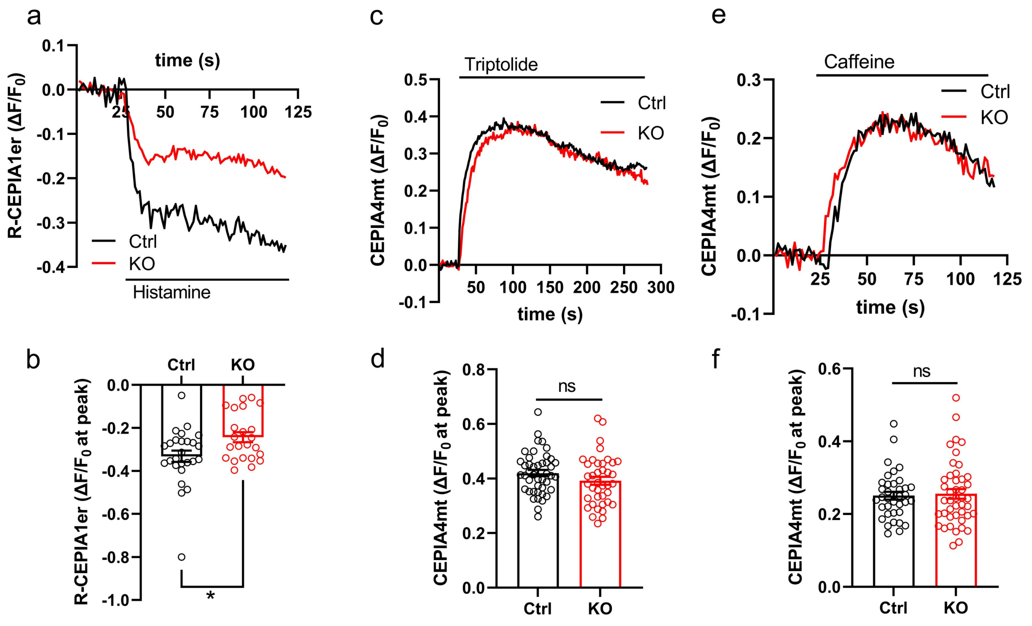

Supplementary Fig. 4 PLA2G6 deficiency reduces mitochondrial Ca^{2+} import primarily through the IP3R1-VDAC1 channel. (a) Representative ER Ca^{2+} traces following histamine (200 µM) stimulation in control and *Pla2g6* KO N2a cells, were measured using the R-CEPIA1er plasmid. (b) Quantification of histamine-stimulated peak values (control, n = 26 cells; *Pla2g6* KO, n = 23 cells from three independent experiments). (c) Representative mitochondrial Ca^{2+} traces following triptolide treatment (200 nM) in control and *Pla2g6* KO N2a cells, were measured using the CEPIA4mt plasmid. (d) Quantification of triptolide-stimulated peak values (control, n = 44 cells; *Pla2g6* KO, n = 39 cells from three independent experiments). (e) Representative mitochondrial Ca^{2+} traces after caffeine treatment (5 mM) in control and *Pla2g6* KO N2a cells, were measured using the CEPIA4mt plasmid. (f) Quantification of caffeine-stimulated peak values (control, n = 37 cells; *Pla2g6* KO, n = 44 cells from three independent experiments). Data are means ± SEM. Two-tailed unpaired *t*-tests were used for statistical analysis (b, d, f). Abbreviation: ns, not significant.

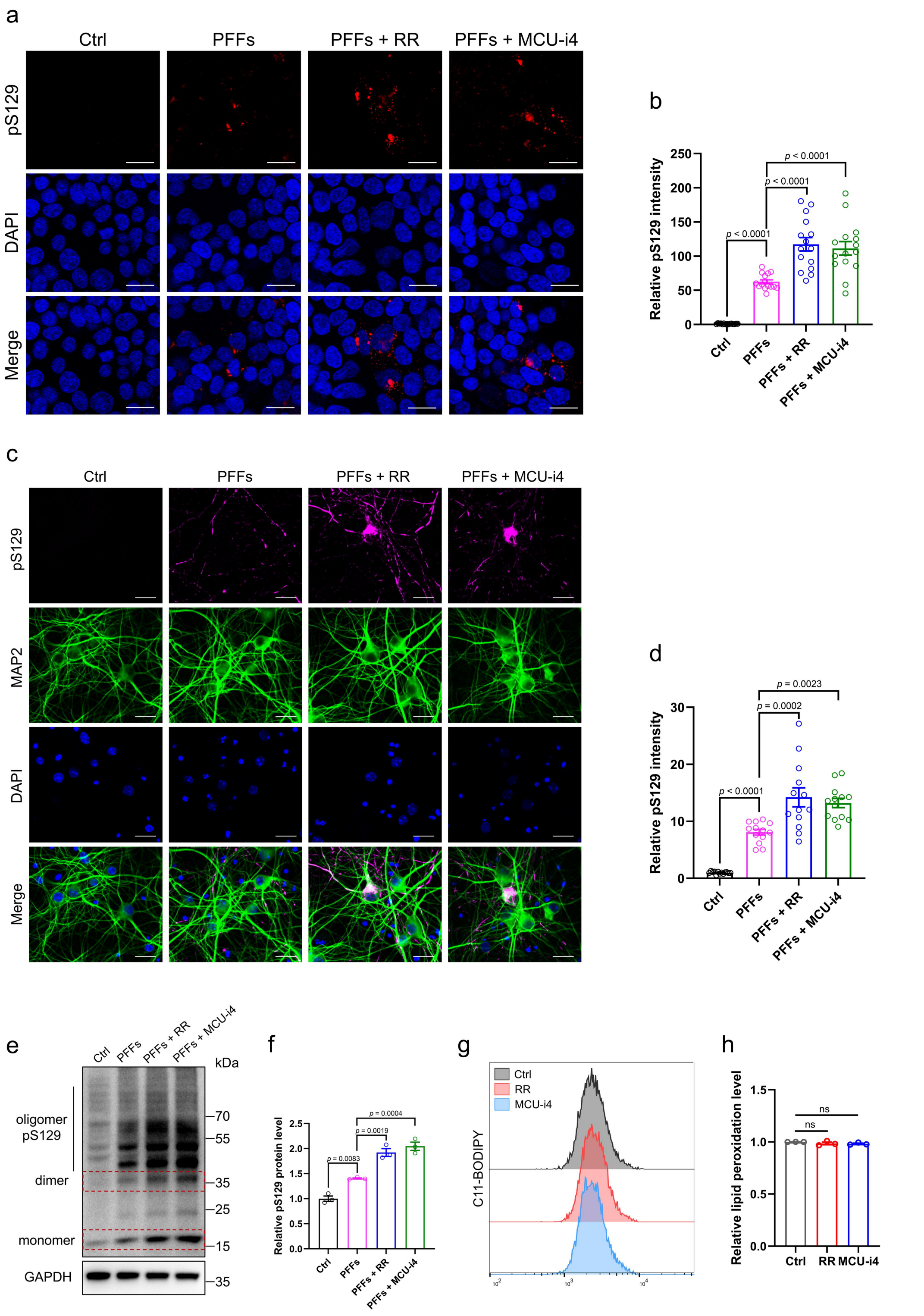

Supplementary Fig. 5 Inhibition of mitochondrial calcium uniporter promotes the aggregation and phosphorylation of α-synuclein (α-Syn). (a) The representative immunofluorescent image of pS129 in α-Syn-HEK293 cells (scale bar, 20 μm). α-Syn-HEK293 cells were treated with 5 μM RR or 2 μM MCU-i4 to inhibit

the MCU and then incubated with α-Syn PFFs for 48 h. DAPI (blue), pS129 (red). (b) Quantitative analysis of pS129 intensity (n = 15 fields of each group from three independent experiments). (c) The representative immunofluorescent image of pS129 in primary neurons (scale bar, 20 μm). Primary neurons were treated with 5 μM RR or 2 μM MCU-i4 together with α-Syn PFFs for 48 h. DAPI (blue), MAP2 (green), pS129 (magenta). (d) Quantitative analysis of pS129 intensity (n = 13 fields of each group from three independent experiments). (e) WB experiment in primary neurons treated with α-Syn PFFs together with RR or MCU-i4. (f) Quantitative analysis of pS129 in primary neurons (n = 3 biologically independent experiments). (g) The lipid peroxidation was measured by flow cytometry using C11-BODIPY. (h) Quantitative analysis of lipid peroxidation in N2a cells (n = 3 biologically independent experiments). Data are means ± SEM. One-way ANOVA with Tukey's multiple comparison tests was used for statistical analysis (b, d, f, h). Abbreviations: PFFs, α-Syn preformed fibrils; RR, ruthenium red; MCU-i4, mitochondrial calcium uniporter-inhibitor 4; pS129, α-synuclein serine129 phosphorylation; MAP2, microtubule Associated Protein 2; ns, not significant.

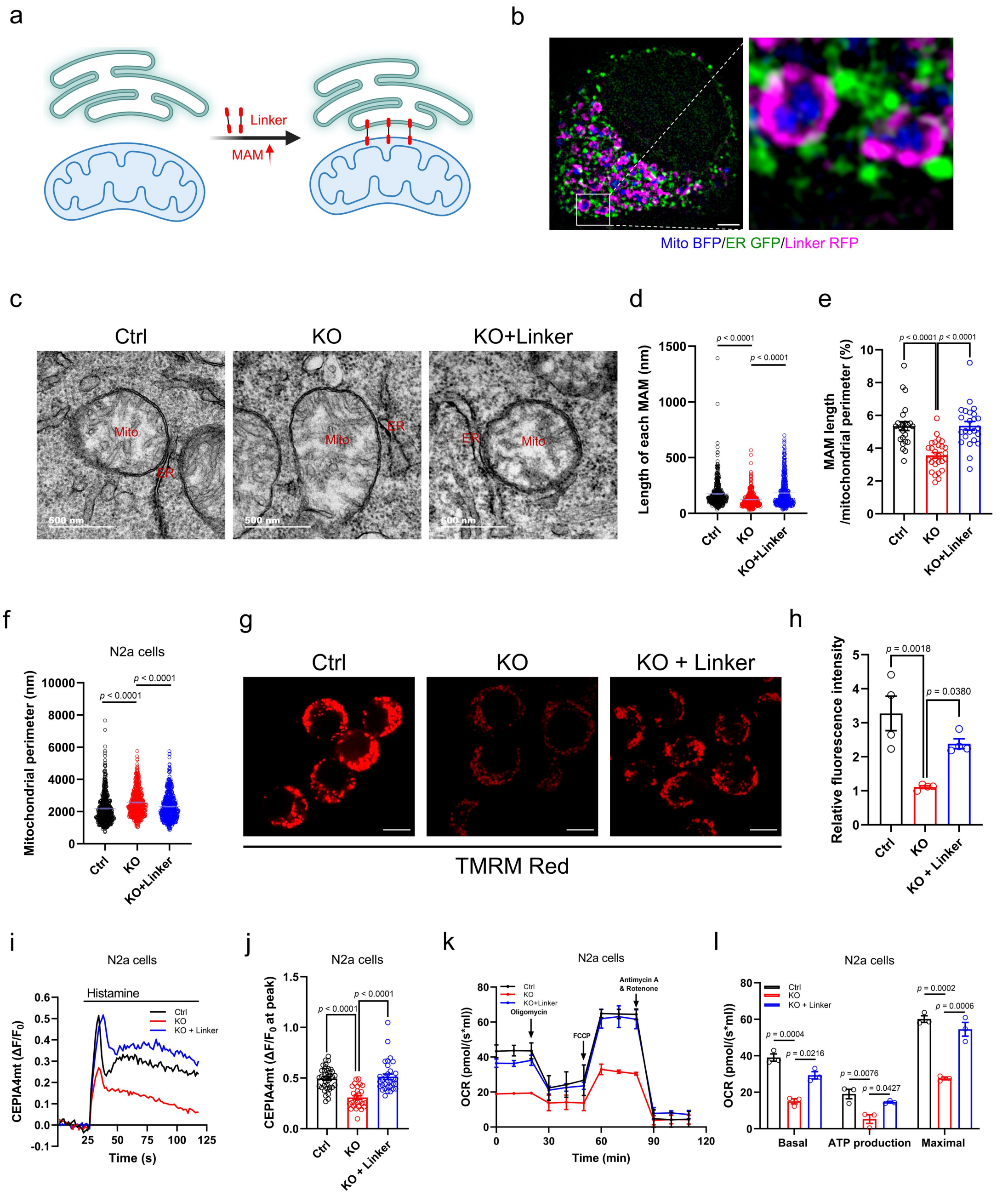

Supplementary Fig. 6 MAM Linker reverses the phenotypes induced by PLA2G6 deficiency. (a) Schematic illustration of MAM formation enhancement using the Linker. (b) The subcellular location of the Linker was analyzed by HIS-SIM in normal N2a cells by expression of Linker-RFP (magenta), Mito-BFP (blue), and ER-GFP (green). The white boxes are magnified on the right (scale bar, 2 μm) (Created in BioRender. Lin, Z. (2026) https://BioRender.com/ydl59ox). (c) Representative TEM micrographs of the control group, *Pla2g6* KO group, and *Pla2g6* KO + Linker group (scale bar, 500 nm) (d, e, f) Determination of length of each

MAM (nm), MAM length/mitochondrial perimeter (%) and mitochondrial perimeter (nm) in control group, *Pla2g6* KO group, and *Pla2g6* KO + Linker group (control group, n = 24 cells and 593 mitochondria; KO group, n = 27 cells and 539 mitochondria; KO + Linker group, n = 24 cells and 598 mitochondria from three independent experiments). (g) Representative mitochondrial membrane potential (red) was tested using TMRM (scale bar, 10 μm). (h) Quantification of mitochondrial membrane potential (n = 4 biologically independent experiments). (i) Representative mitochondrial Ca^{2+} traces following histamine treatment (200 μM) were assessed using CEPIA4mt plasmid. (j) Quantification of histamine-stimulated peak values (control, n = 36 cells; *Pla2g6* KO, n = 28 cells; *Pla2g6* KO + Linker, n = 32 cells from three independent experiments). (k) The OCR measurement in each group. (l) Quantification of basal, ATP production, and maximal OCR in two million cells from each group (n = 3 biologically independent experiments). Data are means ± SEM. One-way ANOVA with Tukey's multiple comparison tests was used for statistical analysis (d, e, f, h, j, l). Abbreviations: Mito, mitochondria; ER, endoplasmic reticulum; TMRM, tetramethylrhodamine.

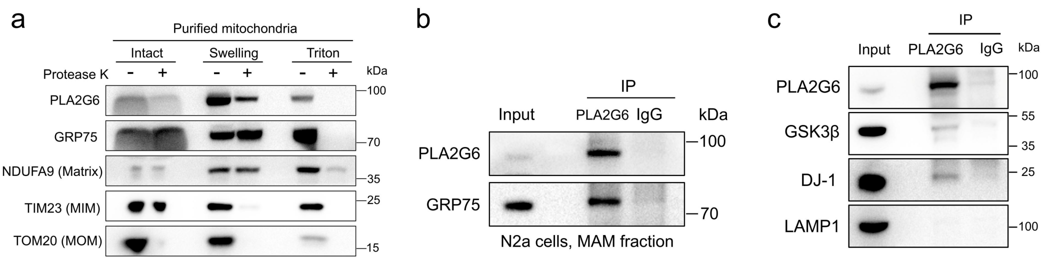

Supplementary Fig. 7 PLA2G6 interacts with GRP75 in the MAM. (a) Determination of the submitochondrial localization of PLA2G6 and GRP75 by Protease K digestion. Intact mitochondria were isolated from N2a cells treated with hypotonic swelling buffer or lysed with Triton X-100 buffer. Different mitochondrial preparations were then digested (n = 3 independent experiments). (b) Immunoblotting analysis of GRP75 in PLA2G6 immunoprecipitates from MAM fractions of normal N2a cells (n = 3 independent experiments). (c) Immunoblotting analysis of GSK3β, DJ-1, and LAMP1 in PLA2G6 immunoprecipitates in normal N2a cells (n = 3 independent experiments). Abbreviations: MAM, mitochondria-associated ER membrane; IP, immunoprecipitation.

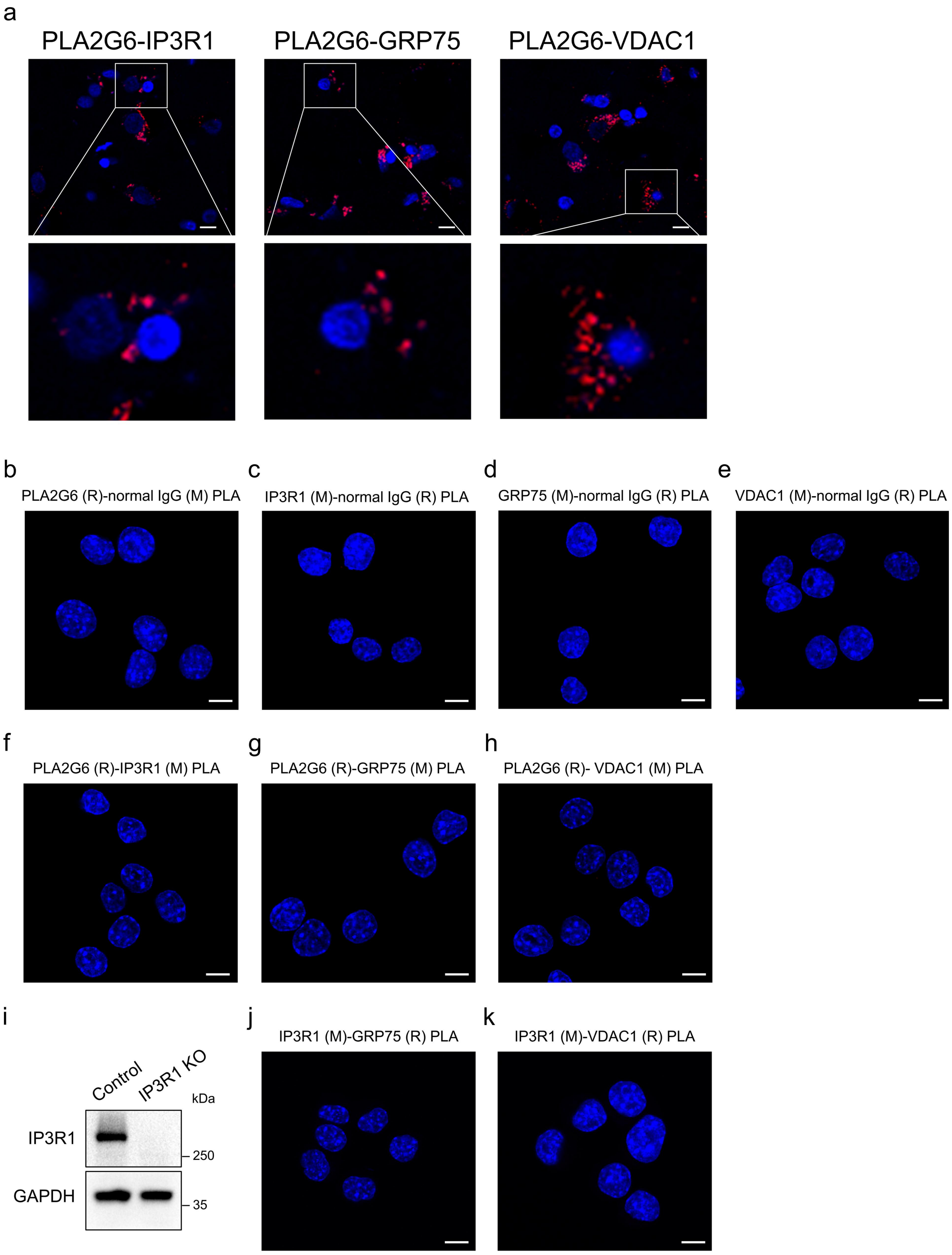

Supplementary Fig. 8 The proximity ligation assay (PLA) in human brains and control experiments. (a) Representative PLA images illustrate the in situ close association between PLA2G6-IP3R1 (left), PLA2G6-GRP75 (middle), and PLA2G6-VDAC1 (right) in human brains (scale bars, 10 μm) (n = 2 independent experiments). (b–e) To exclude the nonspecific binding of antibodies, we employed antibodies and IgG from different species to label normal N2a cells (scale bar, 10 μm).

(f–h) Biological control experiments for PLA were conducted by labeling using antibodies targeting PLA2G6-IP3R1 (f), PLA2G6-GRP75 (g), and PLA2G6-VDAC1 (h) in *Pla2g6* KO N2a cells (scale bar, 10 μm). In all control experiments, PLA signals were not detected. (i) A stable *IP3R1* KO N2a monoclonal cell line was established using the CRISPR/Cas9 method. (j, k) Biological control experiments for PLA were conducted by labeling using antibodies targeting IP3R1-GRP75 (j), and IP3R1-VDAC1 (k) in *IP3R1* KO N2a cells (scale bar, 10 μm). Abbreviation: PLA, the proximity ligation assay. R and M denote the species targeted by the secondary antibodies used, namely rabbit and mouse, respectively.

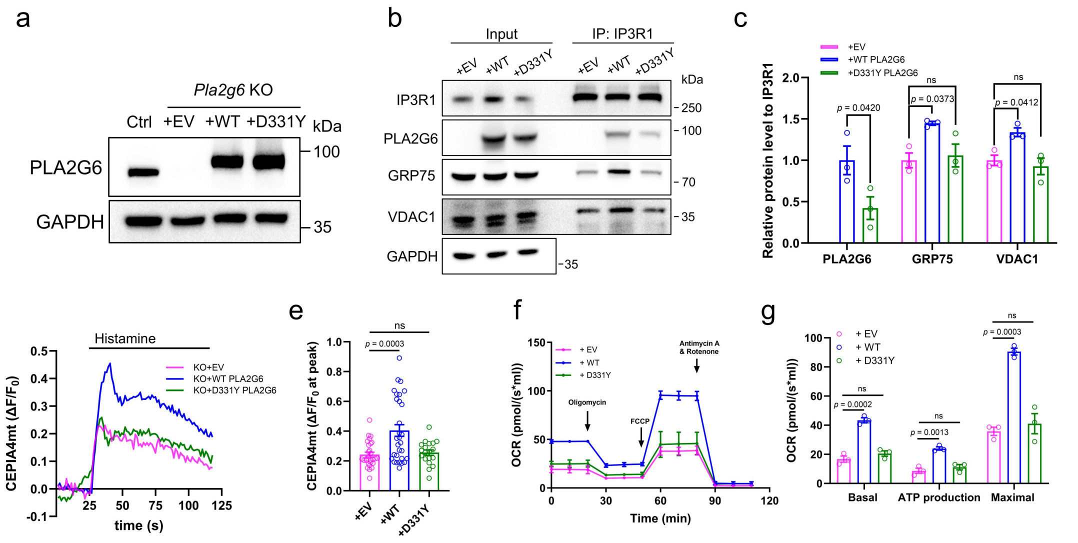

Supplementary Fig. 9 WT but not the PD-associated D331Y mutant *Pla2g6* rescues the *Pla2g6* KO-induced MAM deficits. (a) Representative immunoblot showing re-expression of PLA2G6 in *Pla2g6* KO N2a cells. (b) Cell lysates were immunoprecipitated using an anti-IP3R1 antibody and probed with antibodies against IP3R1, PLA2G6, GRP75, and VDAC1 through WB. (c) Quantitative analysis of IP3R1, PLA2G6, GRP75, and VDAC1 in the precipitates (n = 3 biologically independent experiments). (d) Representative mitochondrial Ca^{2+} traces after histamine treatment (200 µM) were assessed using the CEPIA4mt plasmid. (e) Quantification of histamine-stimulated peak values (*Pla2g6* KO + EV, n = 28 cells; *Pla2g6* KO + WT, n = 30 cells; *Pla2g6* KO + D331Y = 19 cells from three independent experiments). (f) Measurement of OCR in each group. (g) Quantification of basal OCR, ATP production, maximal OCR, and reserve capacity in two million cells per group (n = 3 biologically independent experiments). Data are presented as means ± SEM. One-way ANOVA with Tukey's multiple comparison tests was used for statistical analysis (c, e, g). Abbreviations: EV, empty vector; FCCP, carbonyl cyanide 4-(trifluoromethoxy) phenylhydrazone.

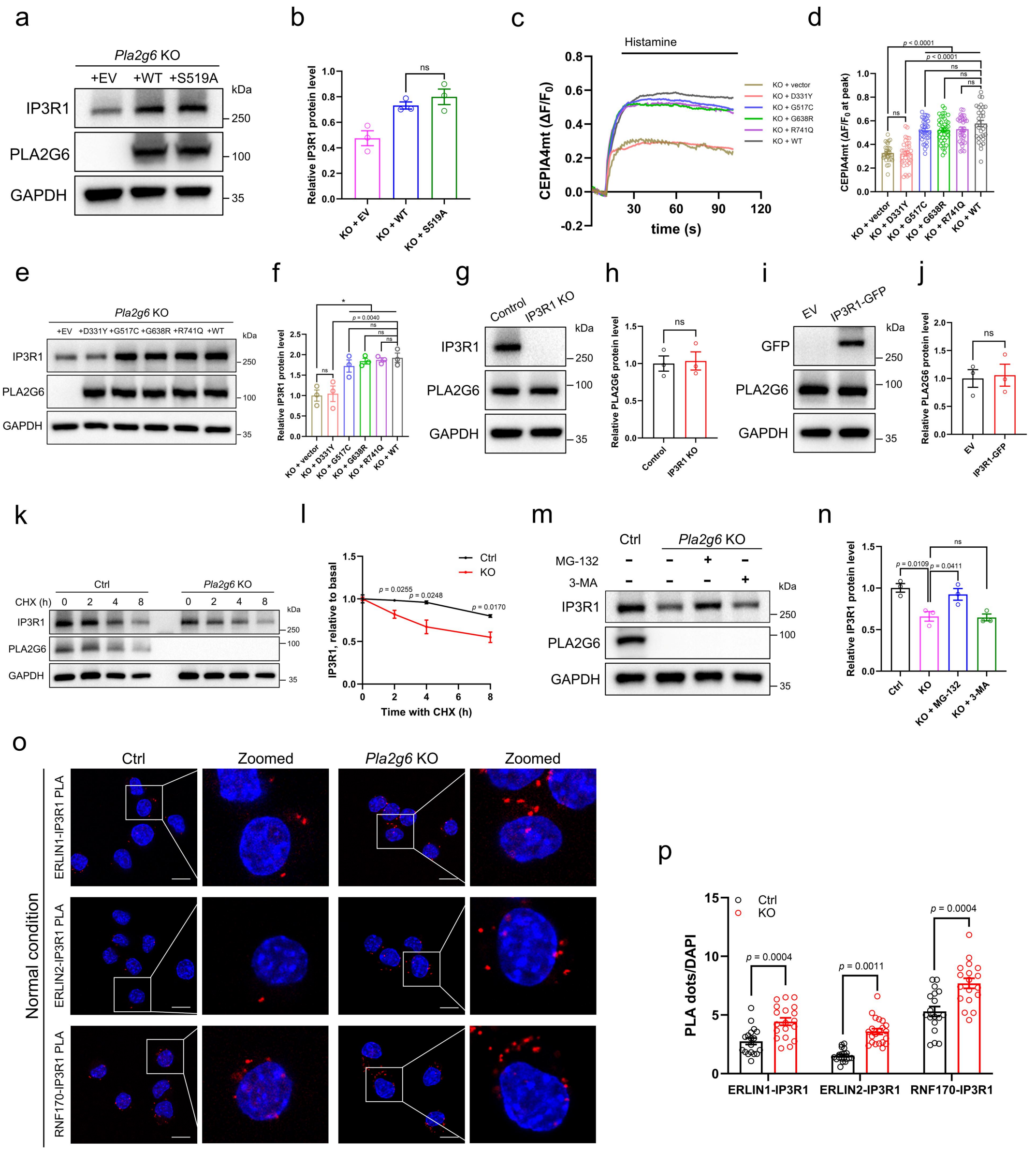

Supplementary Fig. 10 PLA2G6 deficiency decreases the expression of IP3R1 protein. (a) Representative immunoblot showing re-expression of WT or S519A mutant PLA2G6 in *Pla2g6* KO N2a cells. (b) Quantitative analysis of PLA2G6 in N2a cells (n = 3 biologically independent experiments). (c) Representative mitochondrial Ca^{2+} traces. (d) Quantification of histamine-stimulated Ca^{2+} peak values (KO + vector, n = 29 cells; KO + D331Y, n = 31 cells; KO + G517C, n = 34 cells; KO + G638R, n = 39 cells; KO + R741Q, n = 34 cells; KO + WT, n = 31 cells from three independent experiments). (e) Representative immunoblot showing re-expression of D331Y, G517C, G638R, R741Q mutant, and WT PLA2G6 in *Pla2g6* KO N2a cells. (f) Quantitative analysis of IP3R1 in N2a cells (n = 3

biologically independent experiments). Vector-G517C, *p = 0.0168; vector-G638R, *p = 0.0056; vector-R741Q, *p = 0.0043; vector-WT, *p = 0.0027. (g) Representative immunoblot showing the expression of PLA2G6 in control and *IP3R1* KO N2a cells. (h) Quantitative analysis of PLA2G6 in control and *IP3R1* KO N2a cells (n = 3 independent experiments). (i) Representative immunoblot showing the expression of PLA2G6 in control and *IP3R1* overexpression N2a cells. (j) Quantitative analysis of PLA2G6 in control and *IP3R1* overexpression N2a cells (n = 3 independent experiments) . (k) Immunoblot analysis of time-dependent degradation of IP3R1 following 100 μg/mL CHX stimulation at different times in control and *Pla2g6* KO N2a cells. (l) Quantitative analysis of changes in IP3R1 protein expression after CHX treatment (n = 3 biologically independent experiments). (m) Immunoblot analysis of the alteration of IP3R1 expression following 10 μM MG-132 or 10 mM 3-MA treatment in *Pla2g6* KO N2a cells. (n) Quantitative analysis of IP3R1 expression following MG-132 or 3-MA treatment in *Pla2g6* KO N2a cells (n = 3 biologically independent experiments). (o) In situ PLA analysis of ERLIN1-IP3R1, ERLIN2-IP3R1, and RNF170-IP3R1 interactions in control and *Pla2g6* KO N2a cells under normal conditions. White boxes in the images are magnified on the right (scale bars, 10 μm). (p) Quantification of PLA dots in control (ERLIN1-IP3R1, n = 184 cells in 18 fields; ERLIN2-IP3R1, n = 169 cells in 19 fields; RNF170-IP3R1, n = 166 cells in 18 fields from three independent experiments) and *Pla2g6* KO N2a cells (ERLIN1-IP3R1, n = 156 cells in 19 fields; ERLIN2-IP3R1, n = 200 cells in 21 fields; RNF170-IP3R1, n = 163 cells in 18 fields from three independent experiments). Data are presented as means ± SEM. Two-tailed unpaired *t*-tests (h, j, l, p) and one-way ANOVA with Tukey's multiple comparison tests (b, d, f, n) were used for statistical analysis. Abbreviations: EV, empty vector; CHX, cycloheximide; PLA, the proximity ligation assay; ns, not significant.

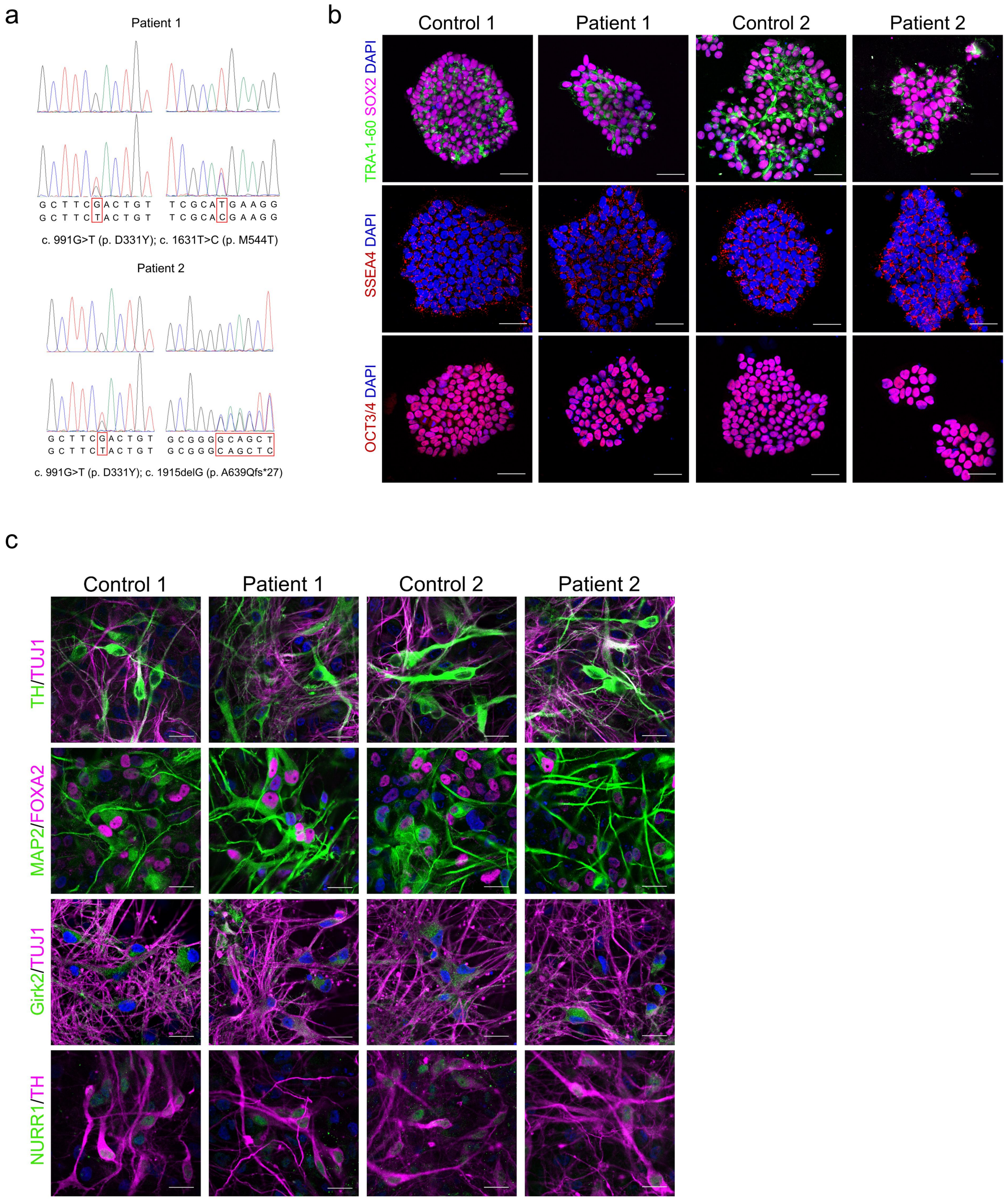

Supplementary Fig. 11 Characterization of iPSCs and iPSCs-derived dopaminergic neurons from healthy controls and PD patients carrying *PLA2G6* mutations. (a) Patient 1 with PD harboring compound heterozygous variants: c.991G>T (p.D331Y), c.1631T>C (p.M544T). Patient 2 with PD harboring compound heterozygous variants: c.991G>T (p.D331Y), c.1915delG (p.A639Qfs*27). (b) Healthy controls and PD patients induced iPSCs express pluripotent markers including TRA-1-60, SOX2, SSEA4, and OCT3/4 (scale bar, 50 µm). (c) Dopaminergic neurons were analyzed by immunofluorescence for the expression of TH, TUJ1, MAP2, FOXA2, GIRK2, and NURR1 (scale bar, 20 µm). Abbreviations:

iPSCs, induced pluripotent stem cells; TRA-1-60, tumor-related antigen 1-60; SOX2, SRY-box transcription factor 2; SSEA4, stage-specific embryonic antigen 4; OCT3/4, octamer-binding transcription factor 3/4; TH, tyrosine hydroxylase; TUJ1, class III beta-tubulin; MAP2, microtubule-associated protein 2; FOXA2, forkhead box A2; Girk2, G protein-activated inwardly rectifying potassium channel 2; NURR1, nuclear receptor related 1 protein.

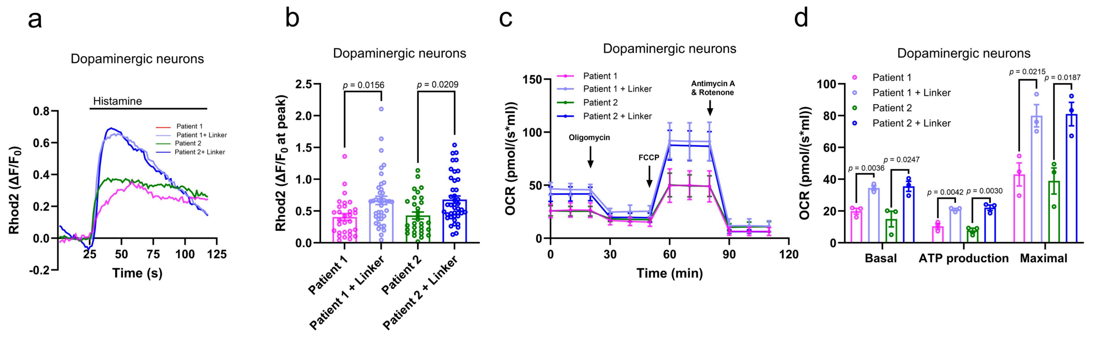

Supplementary Fig. 12 The MAM Linker rescues the reduction of mitochondrial Ca^{2+} import and ATP production in *PLA2G6* mutant neurons. (a) Representative traces of mitochondrial Ca^{2+} following histamine treatment (200 µM) in dopaminergic neurons from controls and patients were assessed using the Rhod2 dye. (b) Quantification of histamine-stimulated peak values (patient 1, n = 30 neurons; patient 1 + Linker, n = 40 neurons; patient 2, n = 30 neurons; patient 2 + Linker, n = 42 neurons from three independent experiments). (c) OCR was measured in dopaminergic neurons from patients and patients + Linker. (d) Quantification of basal OCR, ATP production, and maximal OCR for two million cells of each group (n = 3 biologically independent experiments). Data are presented as means ± SEM. One-way ANOVA with Tukey's multiple comparison tests was used for statistical analysis (b, d). Abbreviation: FCCP, carbonyl cyanide 4-(trifluoromethoxy) phenylhydrazone.

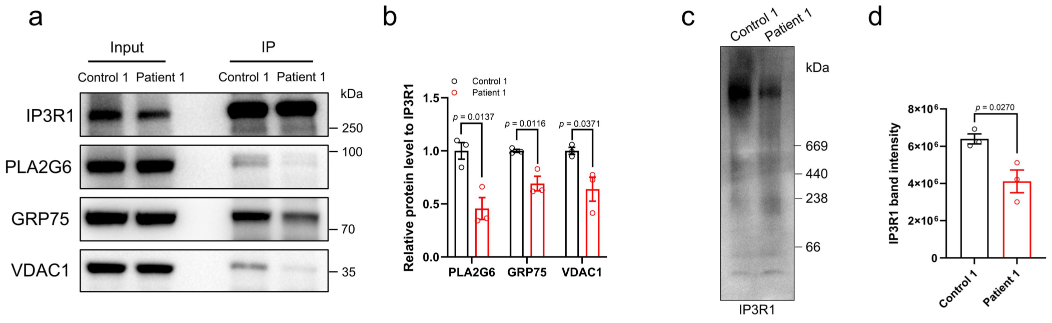

Supplementary Fig. 13 PLA2G6 mutation disrupts the IP3R1-GRP75-VDAC1 complex in iPSCs-derived dopaminergic neurons. (a) Immunoprecipitation of IP3R1 from lysates of Control 1 and Patient 1-derived neurons, followed by WB using IP3R1, PLA2G6, GRP75, and VDAC1 antibodies. (b) Quantitative analysis of IP3R1, GRP75, PLA2G6, and VDAC1 in immunoprecipitates (n = 3 biologically independent experiments). (c) BN-PAGE image showing the IP3R1 complex in crude mitochondria from Control and Patient 1-derived dopaminergic neurons. (n) Quantitative analysis of the IP3R1 complex in crude mitochondria from Control and Patient 1-derived neurons (n = 3 biologically independent experiments). Data are presented as means ± SEM.Two-tailed unpaired *t*-tests were used for statistical analysis (b, d). Abbreviation: IP, immunoprecipitation.

Table S1 The antibodies used in PLA assays

Primary antibodies	Rabbit anti-X	Mouse anti-X
Fig. 3d	PLA2G6 (Proteintech, 22030-1-AP, 1:200)	IP3R1 (Santa Cruz, sc-271197, 1:50)
Fig. 3d	PLA2G6 (Proteintech, 22030-1-AP, 1:200)	GRP75 (Santa Cruz, sc-133137, 1:50)
Fig. 3d	PLA2G6 (Proteintech, 22030-1-AP, 1:200)	VDAC1 (Abcam, ab14734, 1:200)
Fig. 4a	VDAC1 (Proteintech, 55259-1-AP, 1:100)	IP3R1 (Santa Cruz, sc-271197, 1:50)
Fig. 4c	GRP75 (CST, 3593, 1:100)	IP3R1 (Santa Cruz, sc-271197, 1:50)
Fig. 4e	VDAC1 (Proteintech, 55259-1-AP, 1:100)	IP3R1 (Santa Cruz, sc-271197, 1:50)
Fig. 4g	GRP75 (CST, 3593, 1:100)	IP3R1 (Santa Cruz, sc-271197, 1:50)
Fig. 5k	ERLIN1 (ABclonal, A14843, 1:100)	IP3R1 (Santa Cruz, sc-271197, 1:50)
Fig. 5k	ERLIN2 (ABclonal, A0781, 1:100)	IP3R1 (Santa Cruz, sc-271197, 1:50)
Fig. 5k	RNF170 (Affinity, DF14713, 1:100)	IP3R1 (Santa Cruz, sc-271197, 1:50)
Fig. S8a	PLA2G6 (Proteintech, 22030-1-AP, 1:200)	IP3R1 (Santa Cruz, sc-271197, 1:50)
Fig. S8a	PLA2G6 (Proteintech, 22030-1-AP, 1:200)	GRP75 (Santa Cruz, sc-133137, 1:50)
Fig. S8a	PLA2G6 (Proteintech, 22030-1-AP, 1:200)	VDAC1 (Abcam, ab14734, 1:200)
Fig. S8b	PLA2G6 (Proteintech, 22030-1-AP, 1:200)	IgG
Fig. S8c	IgG	IP3R1 (Santa Cruz, sc-271197, 1:50)
Fig. S8d	IgG	GRP75 (Santa Cruz, sc-133137, 1:50)
Fig. S8e	IgG	VDAC1 (Abcam, ab14734, 1:200)
Fig. S8f	PLA2G6 (Proteintech, 22030-1-AP, 1:200)	IP3R1 (Santa Cruz, sc-271197, 1:50)
Fig. S8g	PLA2G6 (Proteintech, 22030-1-AP, 1:200)	GRP75 (Santa Cruz, sc-133137, 1:50)
Fig. S8h	PLA2G6 (Proteintech, 22030-1-AP, 1:200)	VDAC1 (Abcam, ab14734, 1:200)
Fig. S8j	GRP75 (CST, 3593, 1:100)	IP3R1 (Santa Cruz, sc-271197, 1:50)
Fig. S8k	VDAC1 (Proteintech, 55259-1-AP, 1:100)	IP3R1 (Santa Cruz, sc-271197, 1:50)
Fig. S10o	ERLIN1 (ABclonal, A14843, 1:100)	IP3R1 (Santa Cruz, sc-271197, 1:50)
Fig. S10o	ERLIN2 (ABclonal, A0781, 1:100)	IP3R1 (Santa Cruz, sc-271197, 1:50)
Fig. S10o	RNF170 (Affinity, DF14713, 1:100)	IP3R1 (Santa Cruz, sc-271197, 1:50)

Table S2 The primers for mutation detection and QPCR.

Mutations or gene	samples	Forward	Reverse
c. 991G>T (*PLA2G6*)	Fibroblasts	CCTCTCAGAGCAGAAGTGGC	TCCTGGGCTCACCGACAT
c. 1631T>C (*PLA2G6*)	Fibroblasts	CTCTGCAGGCTGTTCTACGG	GTCCGTCATCTTGGTGTGCT
c. 1915delG (*PLA2G6*)	Fibroblasts	ACCAGGACGAACTAGCCAGA	TCGGTCCCTAGCATGGTTTG
IP3R1	N2a cells	CGATGACATCGTTCGTGTGGTC	CACCTCCGTATCCACATAGCAG
GAPDH	N2a cells	GGTTGTCTCCTGCGACTTCA	TGGTCCAGGGTTTCTTACTCC
