# Supplementary material for: Parkinson’s disease-associated PLA2G6 protects IP3R1 protein to control ER-mitochondria tethering and Ca2+ transfer
Source: Nat Commun. 2026 Mar 19;17:5338. doi: 10.1038/s41467-026-70752-1 (PMC13272647; doi:10.1038/s41467-026-70752-1)
Supplement: Supplementary file 2 — Description of Additional Supplementary Files [file 41467_2026_70752_MOESM2_ESM.pdf]

<u>Description of Additional Supplementary Files</u>

File name: Supplementary movie 1

Description: Serial z-stack images of U2OS cells

The ER, mitochondria, and PLA2G6 were labeled with green, blue, and magenta, respectively.

File name: Supplementary movie 2

Description: 3D FIB-SEM image series and animation showing reconstructed ER, mitochondria, and ER-mitochondria contacts in control N2a cells

The ER and mitochondria were labeled in magenta and green, respectively, followed by the display of ER-mitochondria contact sites and mitochondria labeled in magenta and green, respectively.

File name: Supplementary movie 3

Description: 3D FIB-SEM image series and animation showing reconstructed ER, mitochondria, and ER-mitochondria contacts in *Pla2g6* KO N2a cells

The ER and mitochondria were labeled in magenta and green, respectively, followed by the display of ER-mitochondria contact sites and mitochondria labeled in magenta and green, respectively.
