# Supplementary material for: Parkinson’s disease-associated PLA2G6 protects IP3R1 protein to control ER-mitochondria tethering and Ca2+ transfer
Source: Nat Commun. 2026 Mar 19;17:5338. doi: 10.1038/s41467-026-70752-1 (PMC13272647; doi:10.1038/s41467-026-70752-1)

Fig. 1a

Representative image

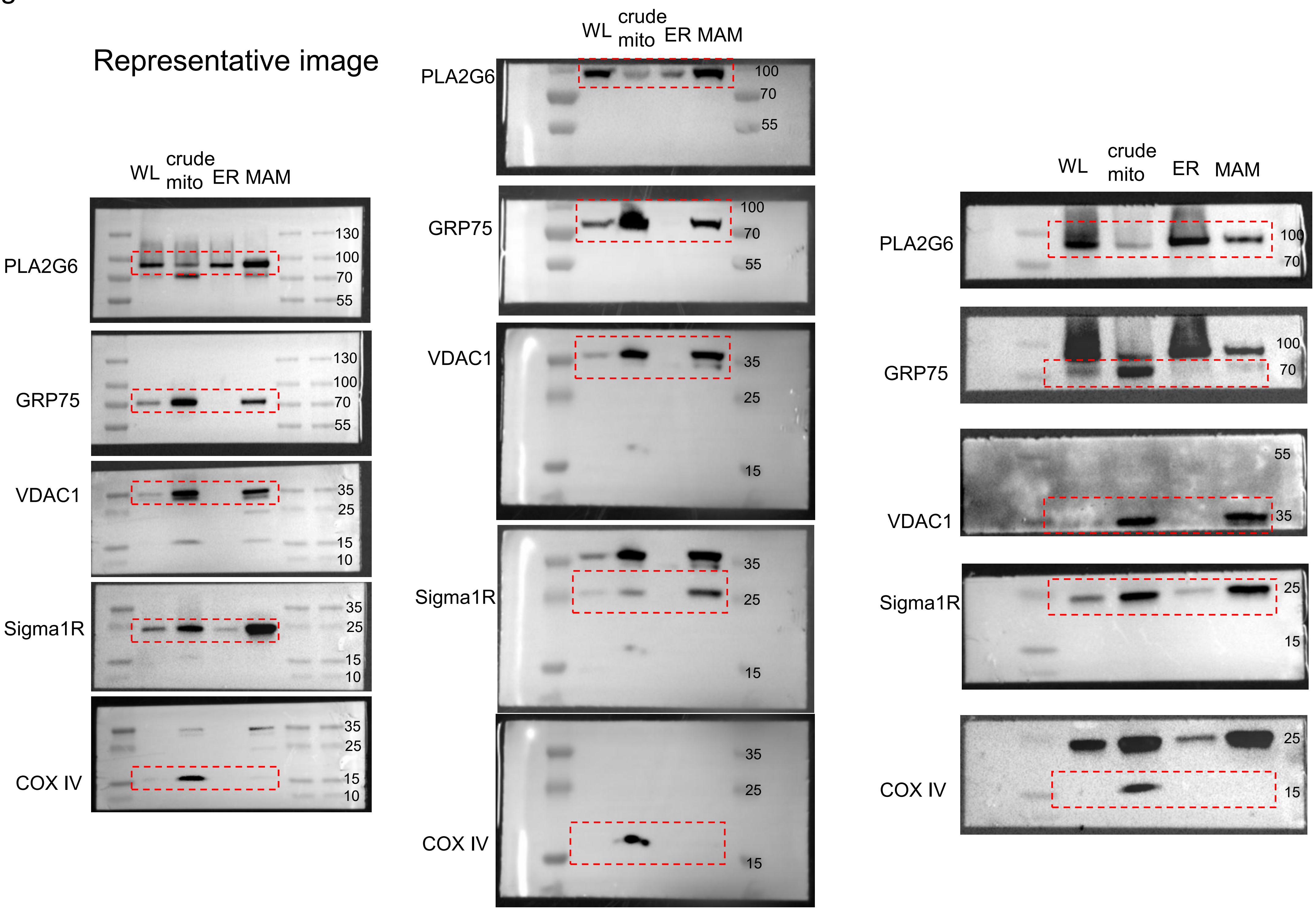

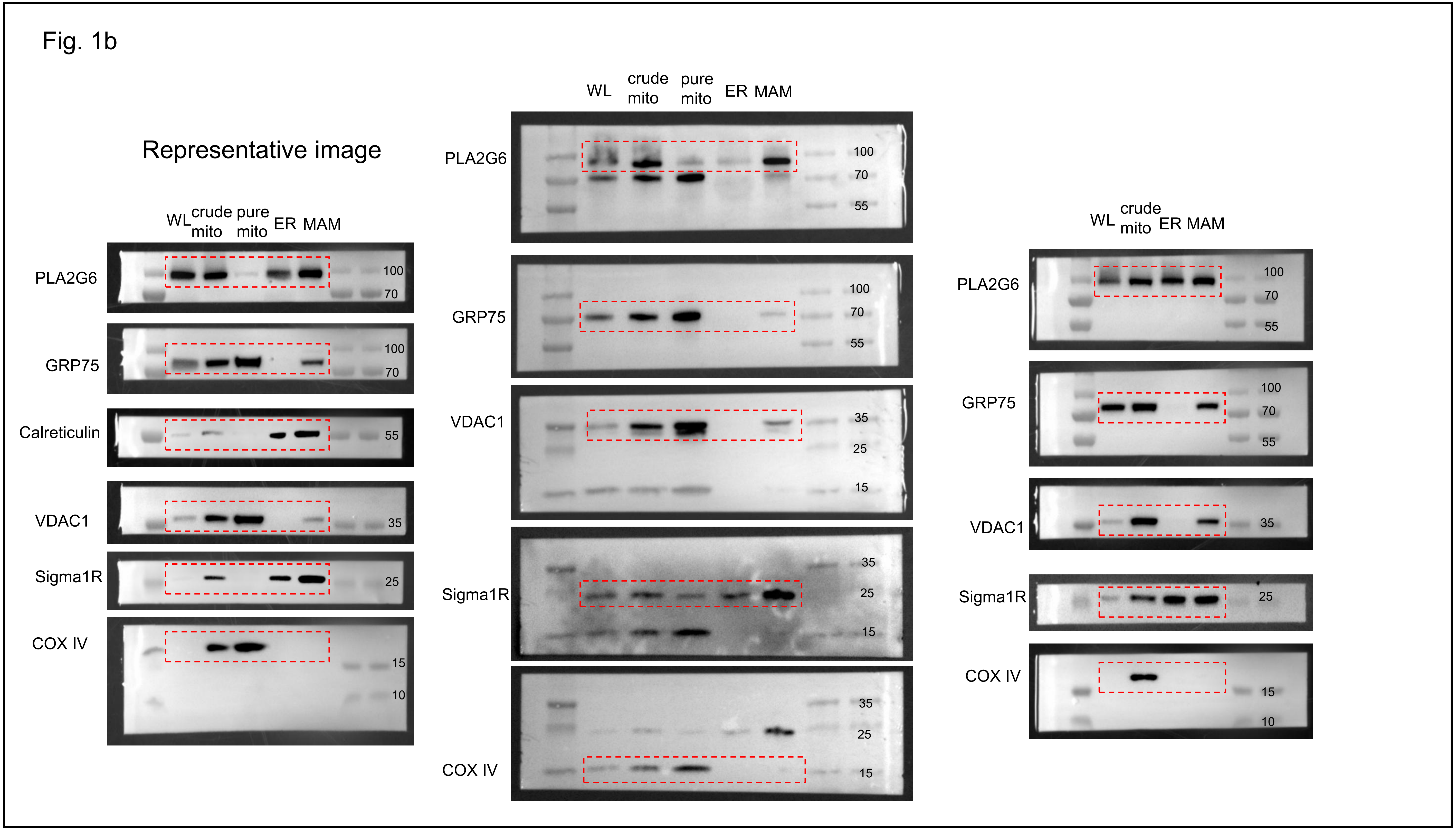
Fig. 1b
Representative image
WL
crude mito
pure mito
ER
MAM
PLA2G6
GRP75
Calreticulin
VDAC1
Sigma1R
COX IV
100
70
55
35
25
15
10

Fig. 1c

Representative image

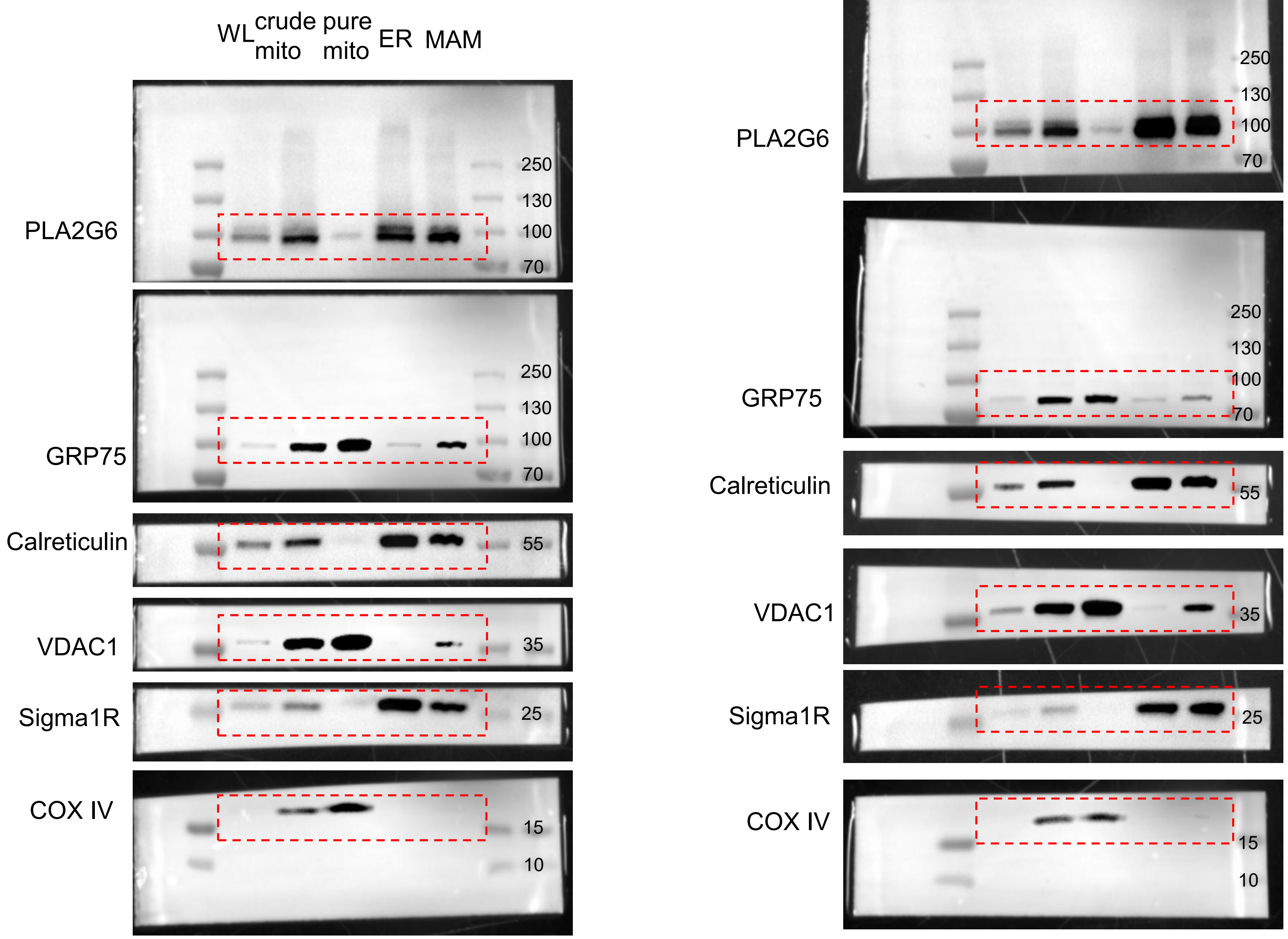

Fig. 3b

Representative image

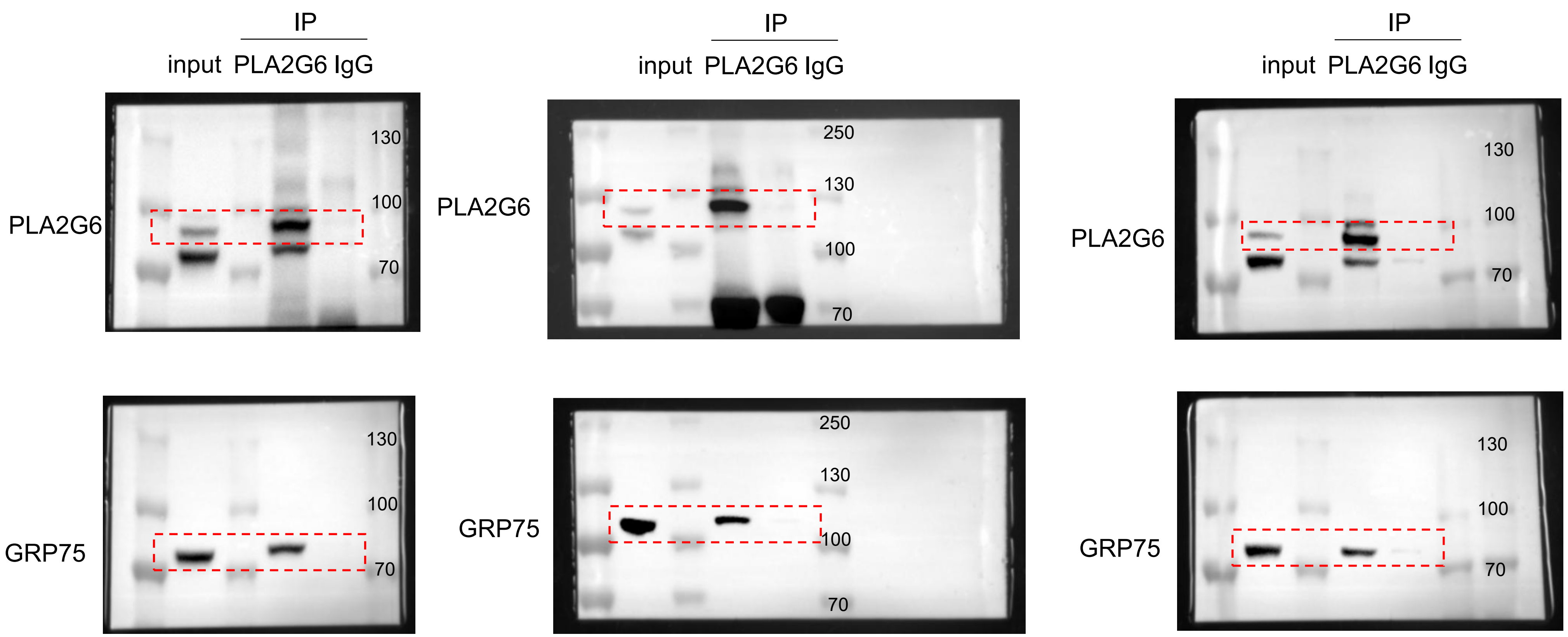

Fig. 3c

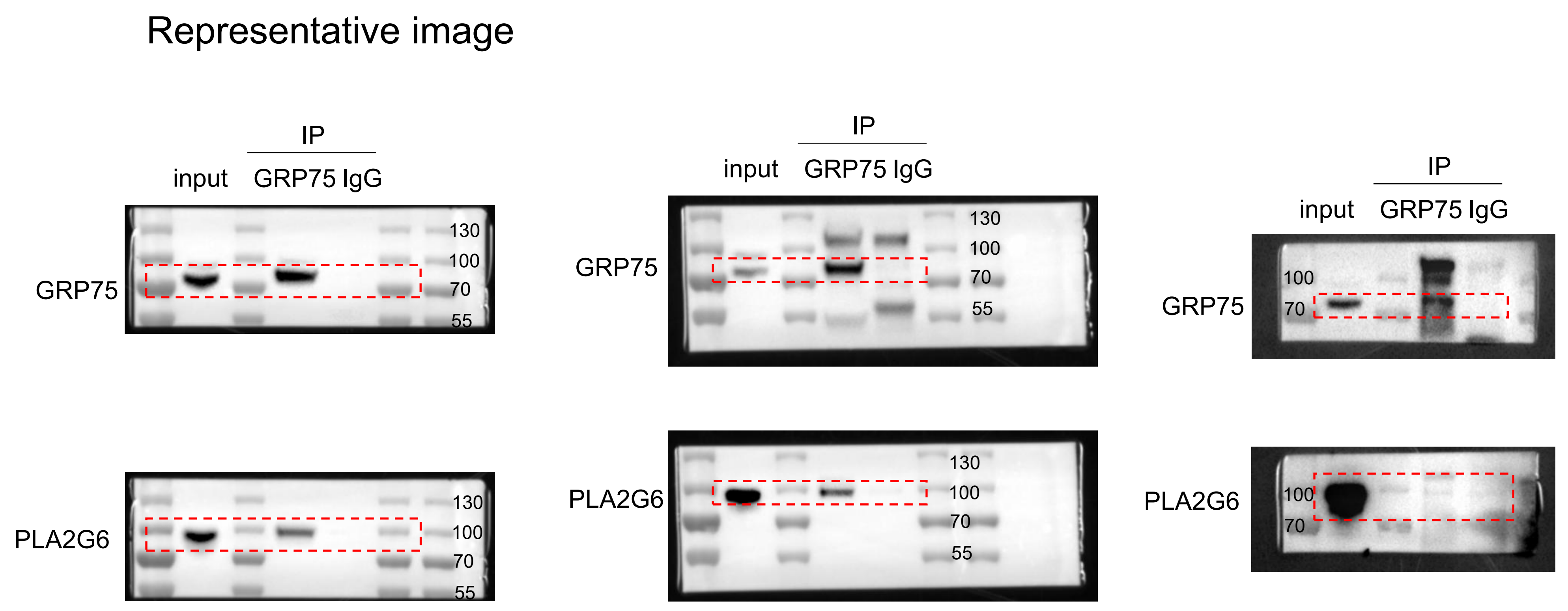
Representative image
IP
input
GRP75 IgG
GRP75
PLA2G6
130
100
70
55
IP
input
GRP75 IgG
GRP75
PLA2G6
130
100
70
55
IP
input
GRP75 IgG
GRP75
PLA2G6
100
70

Fig. 3e

Representative image

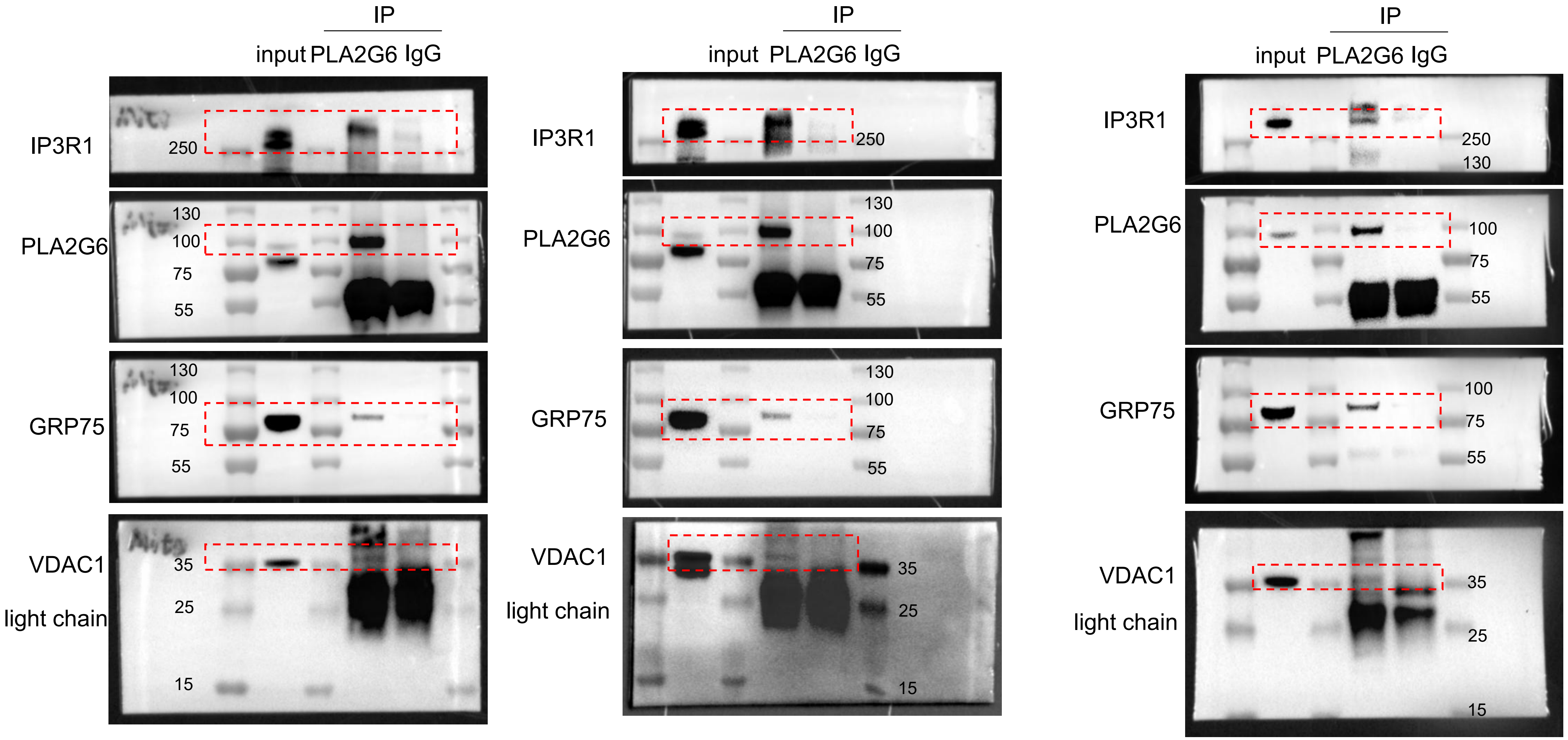

Fig. 3f

Representative image

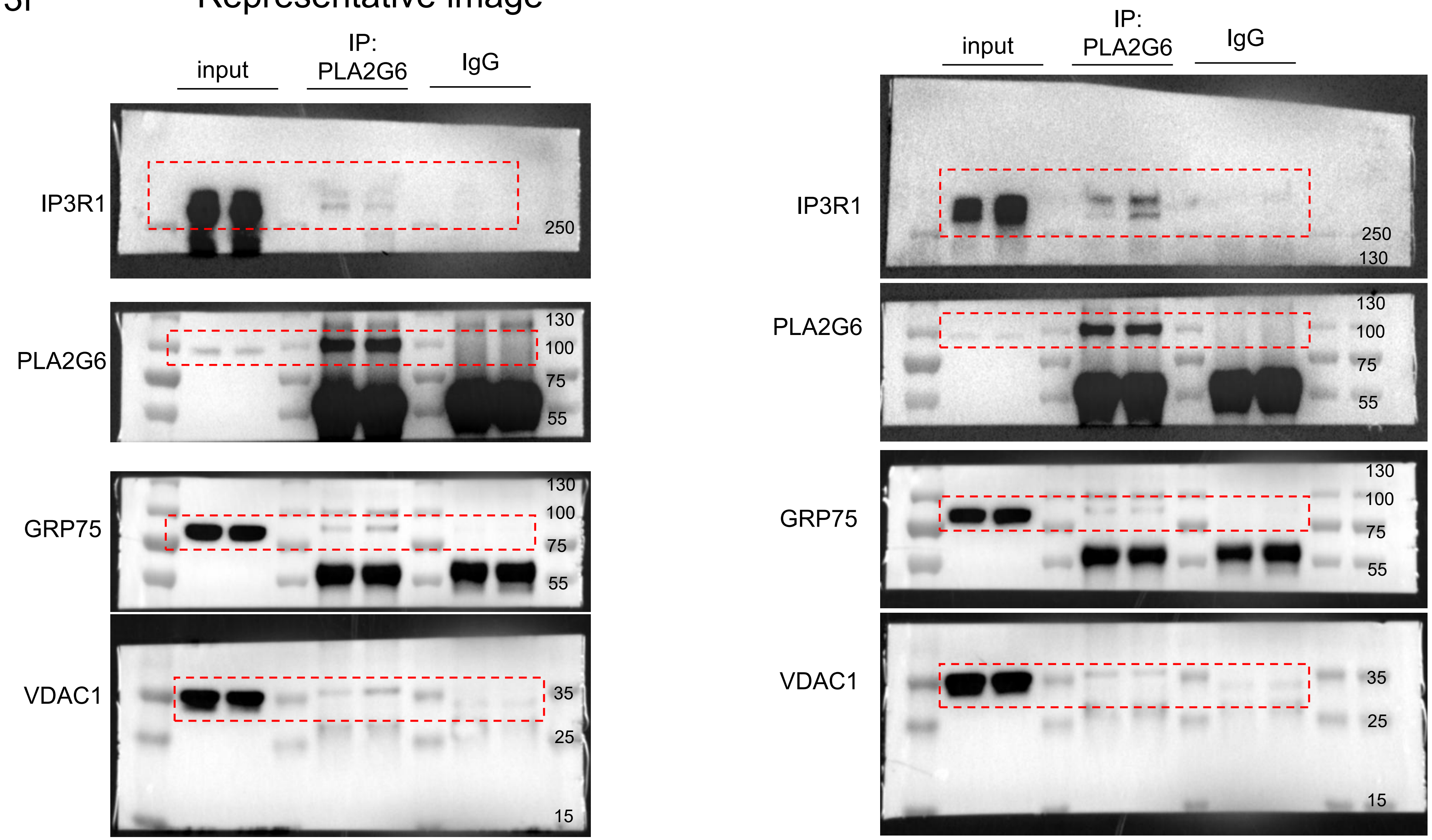

Fig. 3g

Representative image

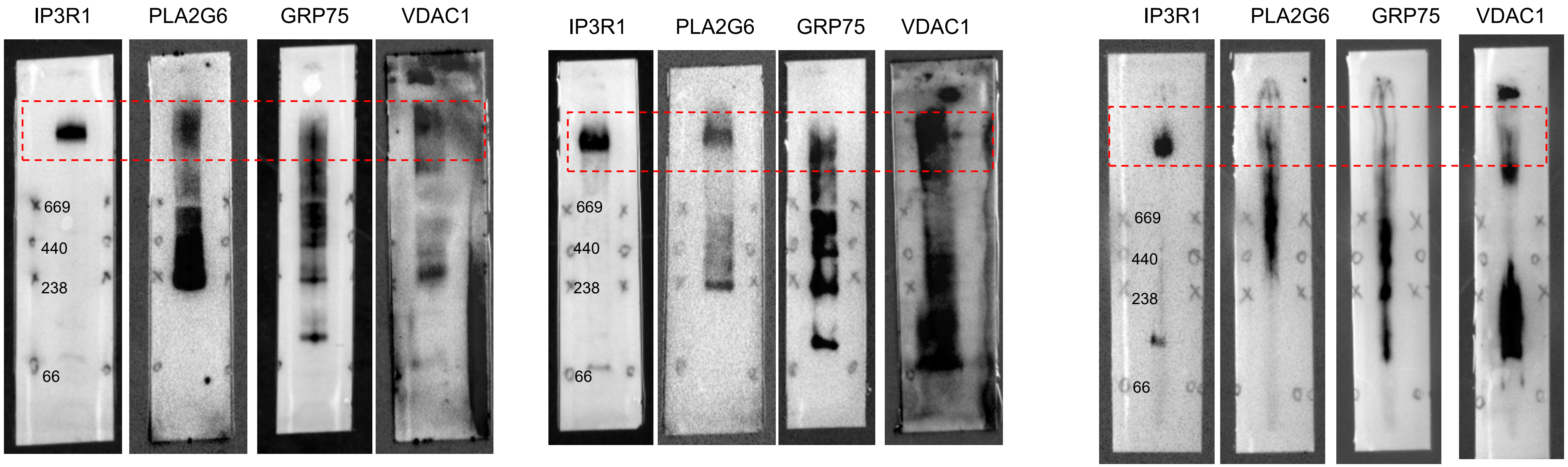

Fig. 3h

Representative image

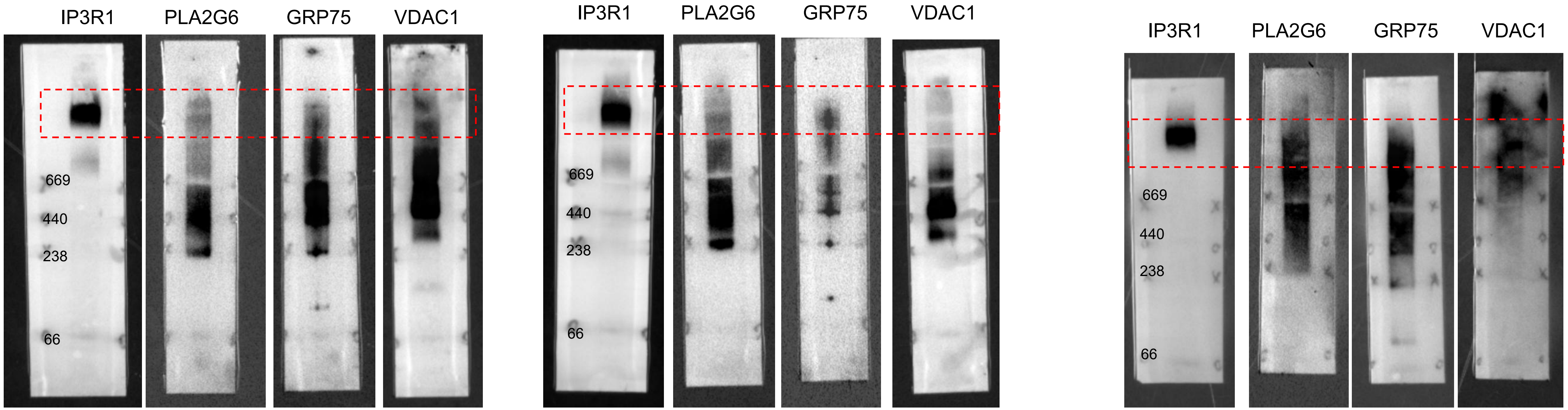

Fig. 3i Representative image

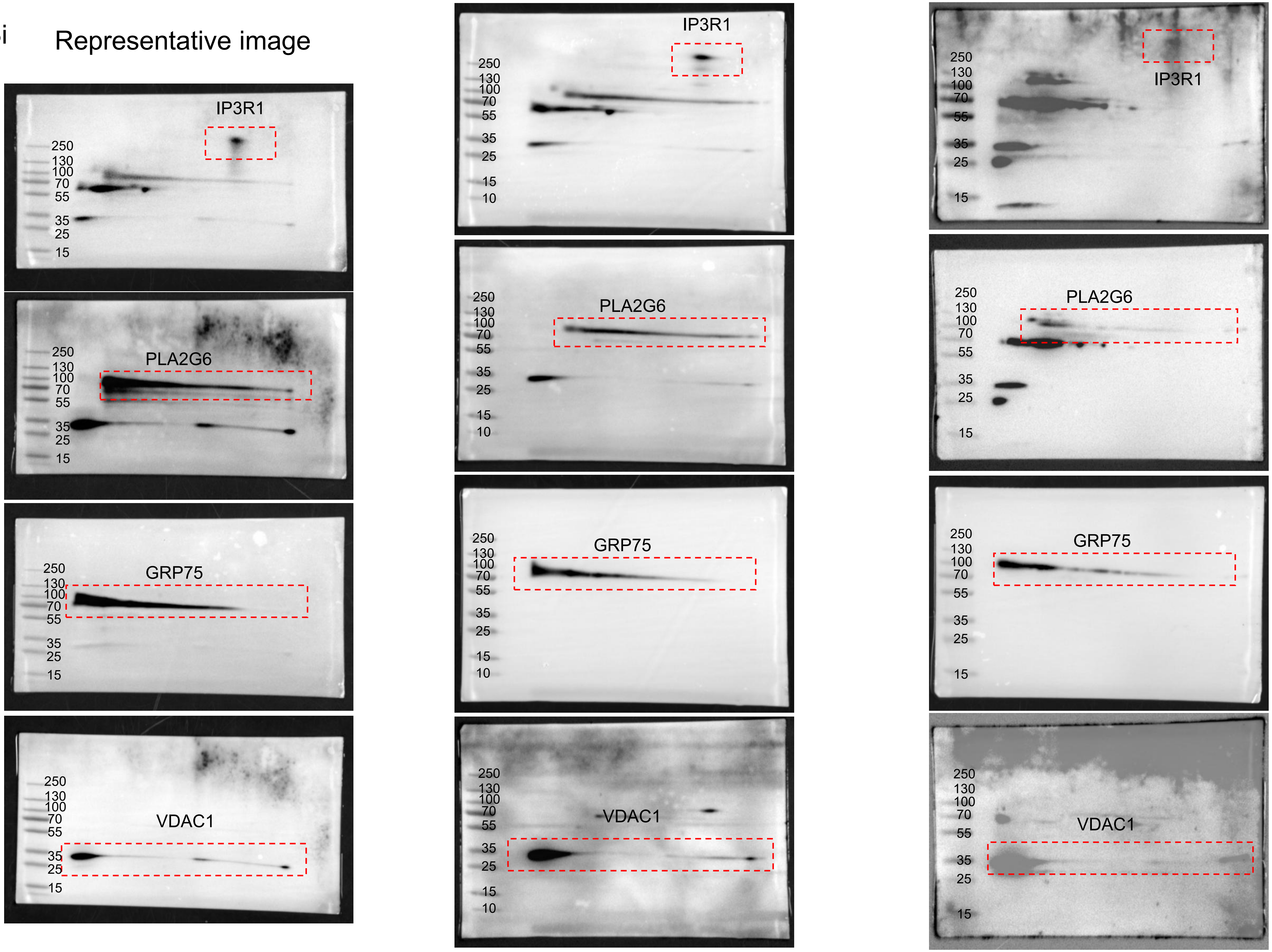

Fig. 4i

Representative image

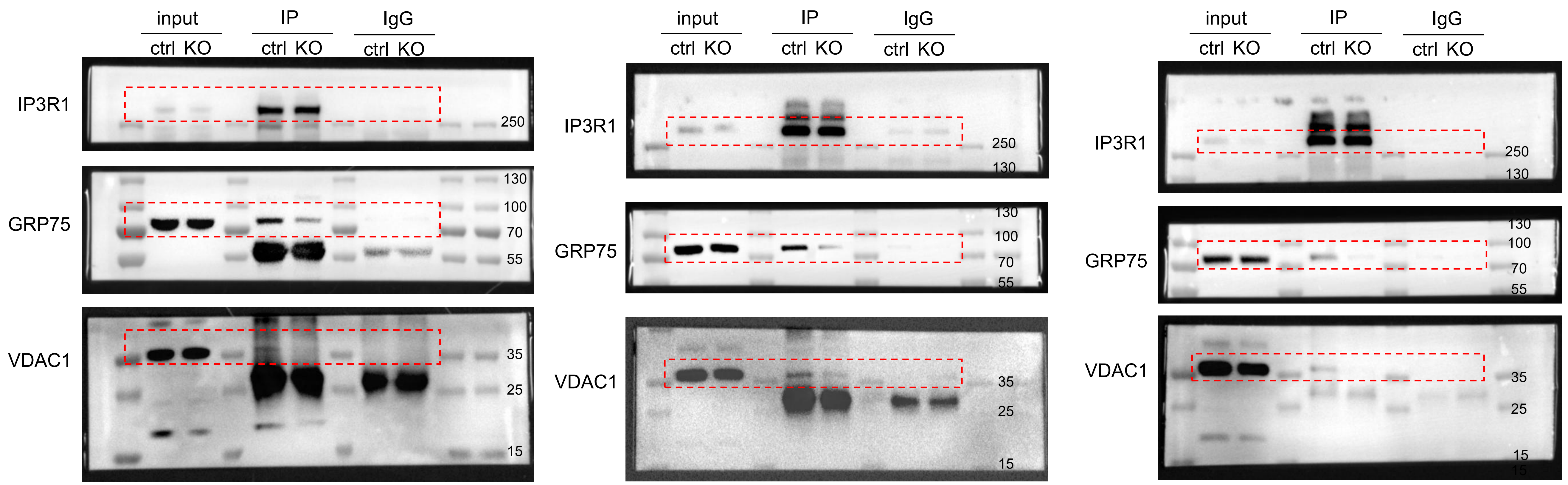

Fig. 4k

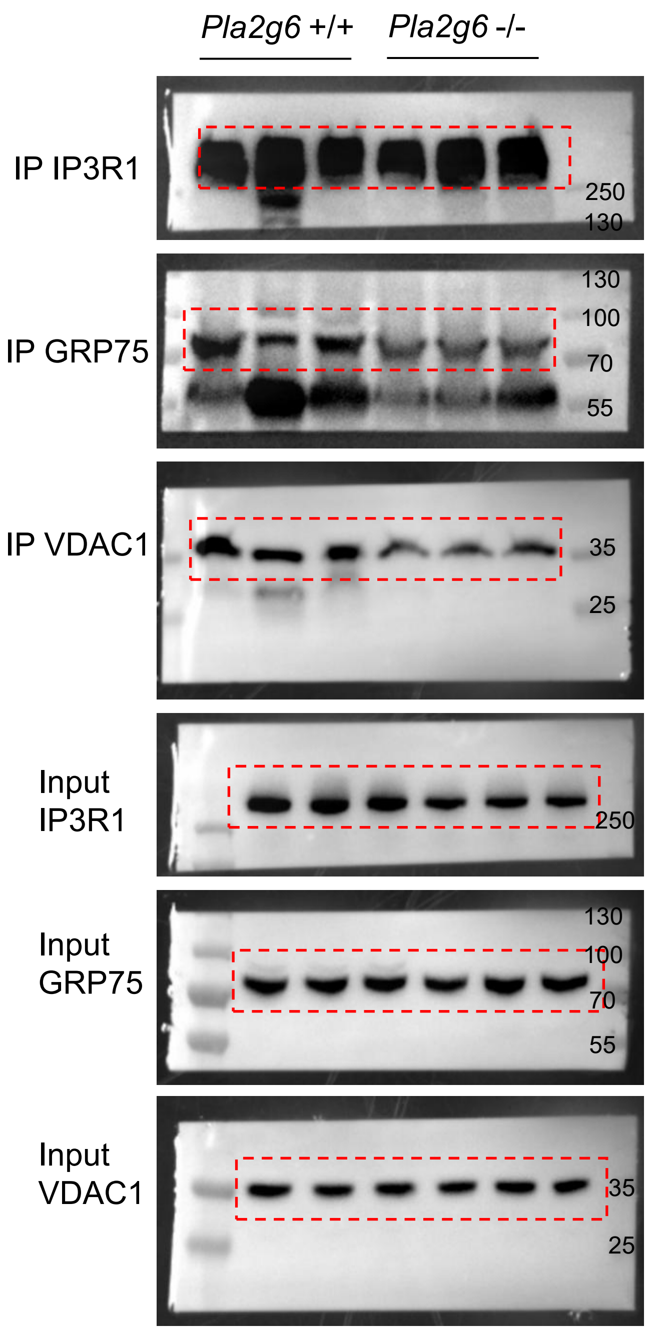

Pla2g6 +/+
Pla2g6 -/-
IP IP3R1
250
130
IP GRP75
130
100
70
55
IP VDAC1
35
25
Input
IP3R1
250
Input
GRP75
130
100
70
55
Input
VDAC1
35
25

Fig. 4m

Representative image

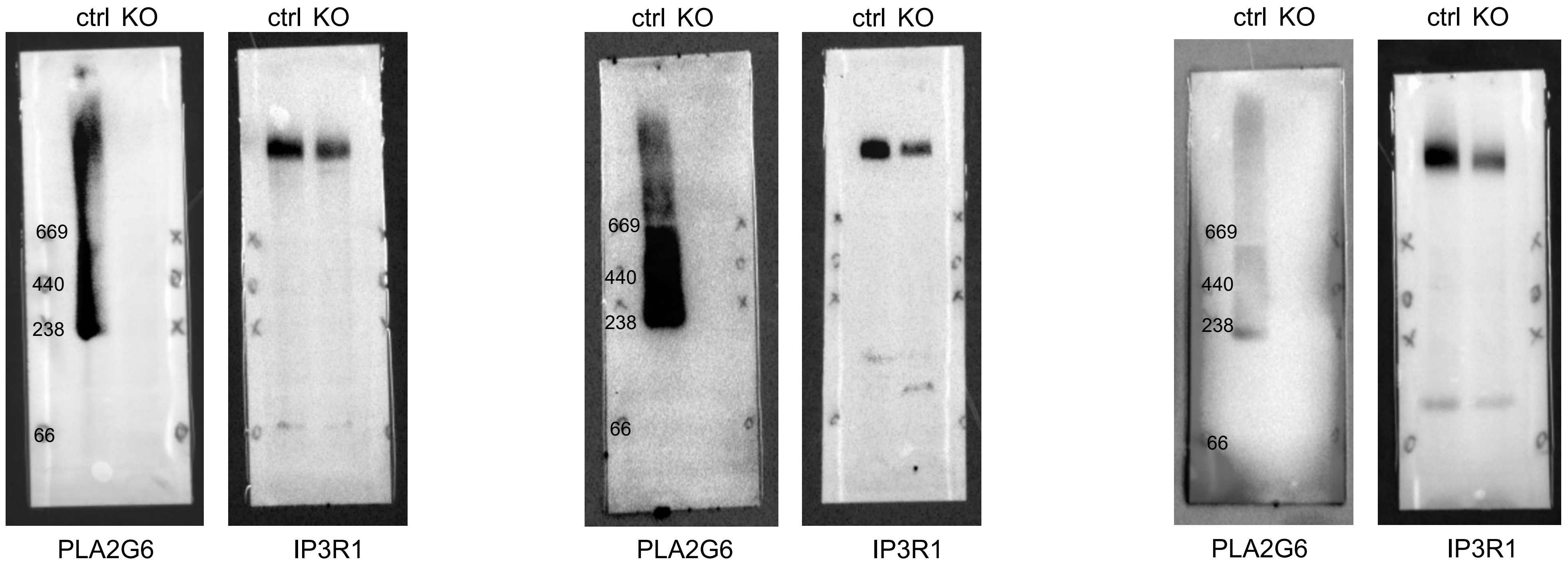

Fig. 4o

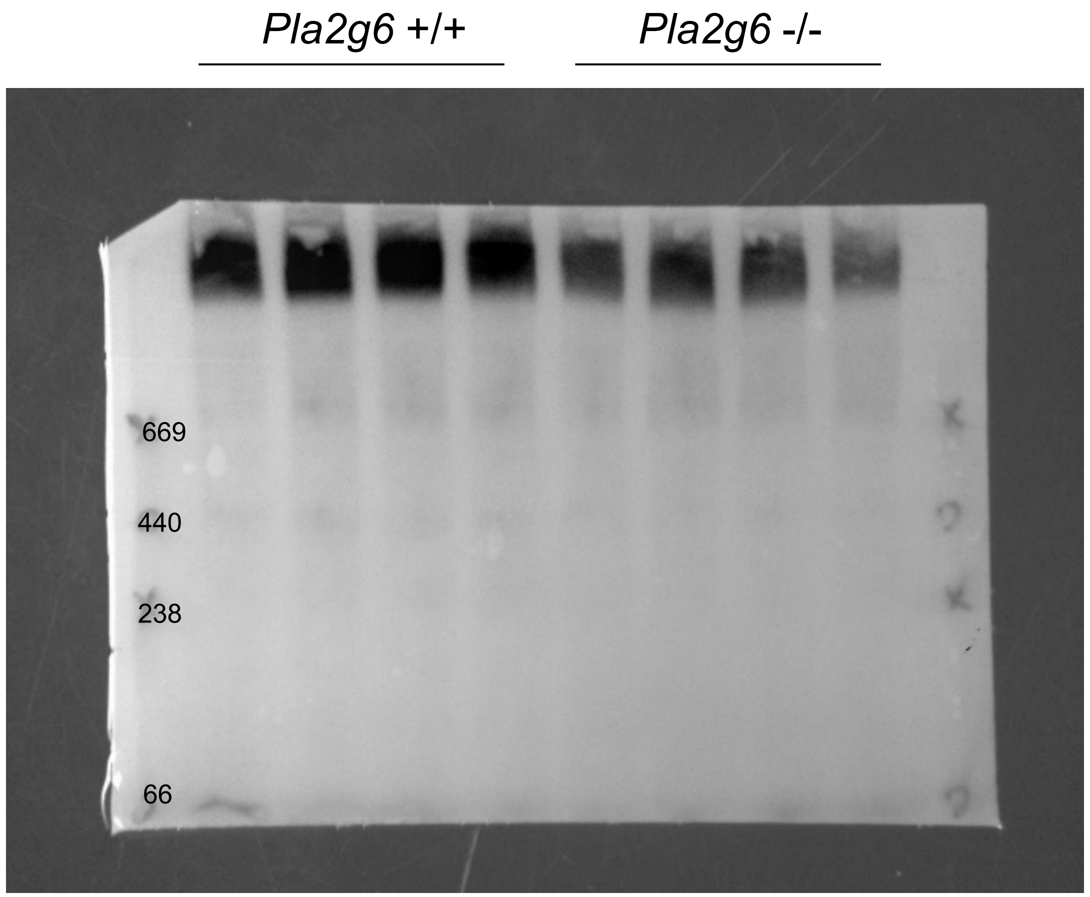

BN-PAGE, IP3R1

Fig. 5a

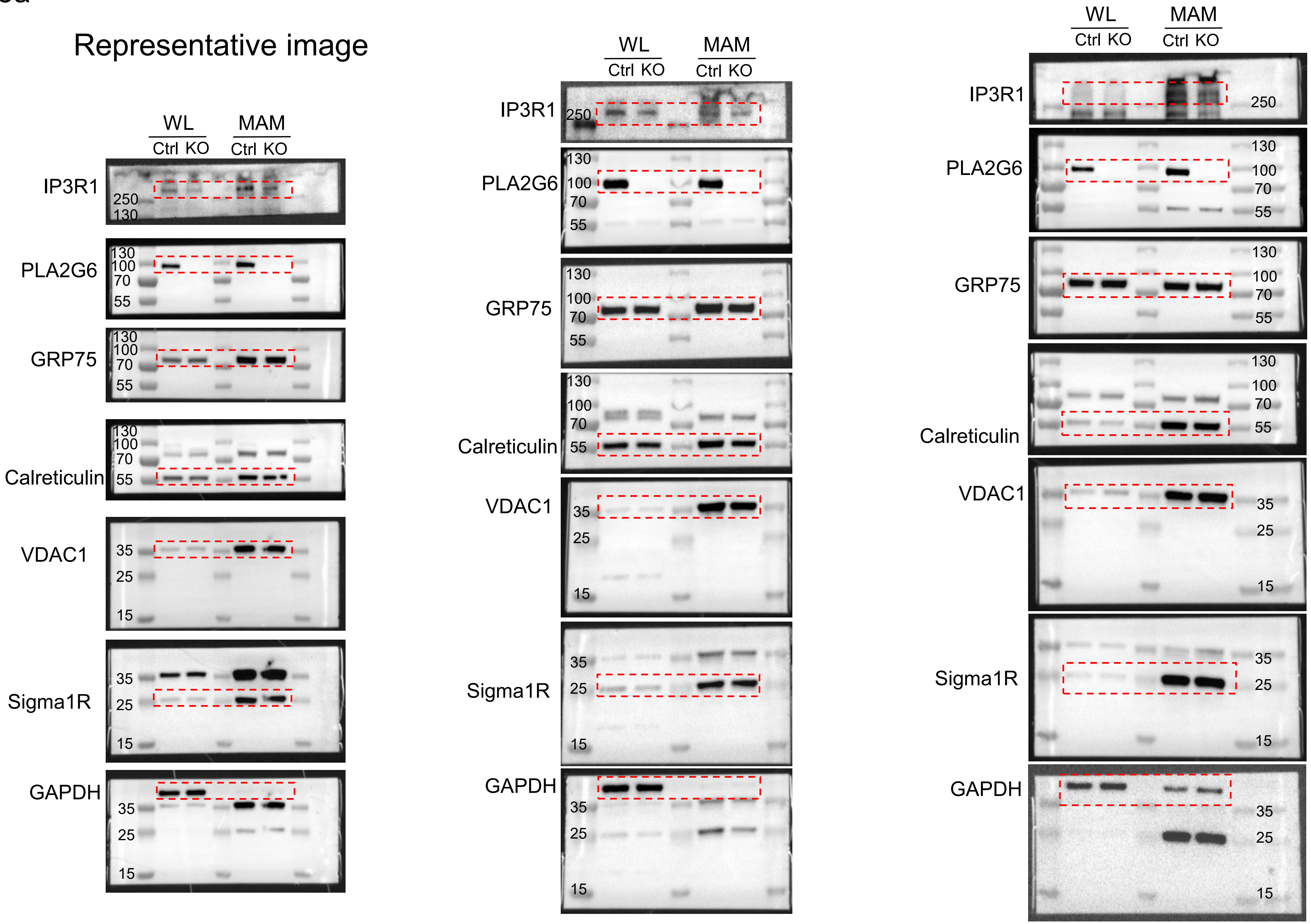
Representative image
WL
MAM
Ctrl KO
Ctrl KO
IP3R1
PLA2G6
GRP75
Calreticulin
VDAC1
Sigma1R
GAPDH
250
130
100
70
55
35
25
15

Fig. 5d

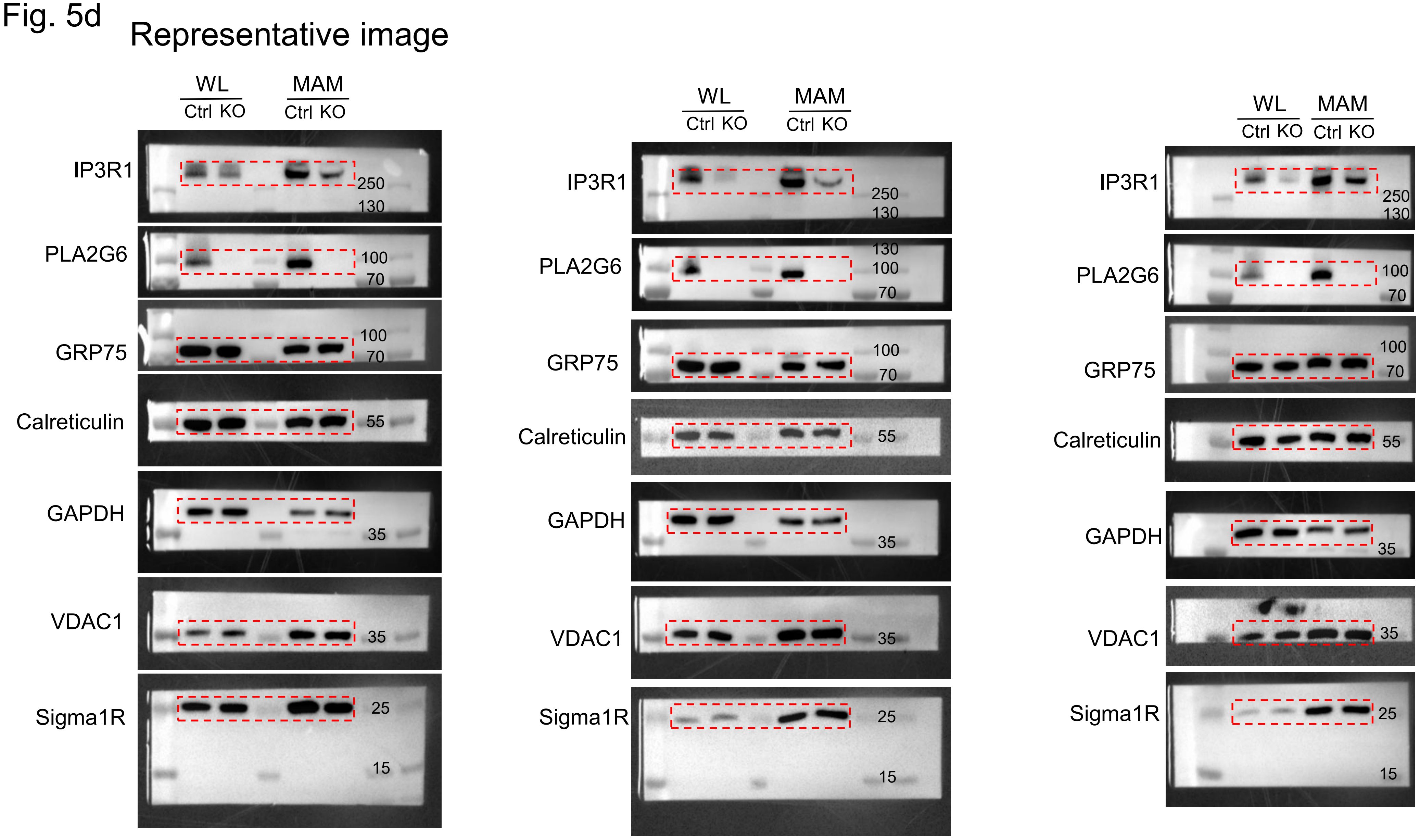
Representative image
WL
MAM
Ctrl KO
Ctrl KO
IP3R1
PLA2G6
GRP75
Calreticulin
GAPDH
VDAC1
Sigma1R
250
130
100
70
55
35
25
15

Fig. 5h

Representative image

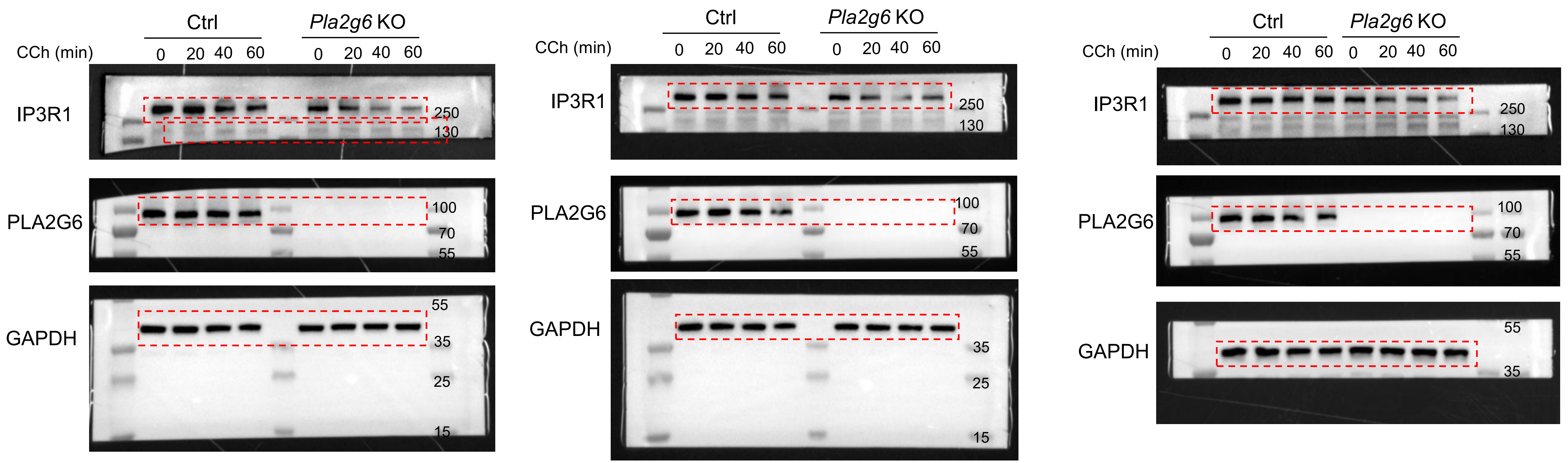

Fig. 5j

Representative image

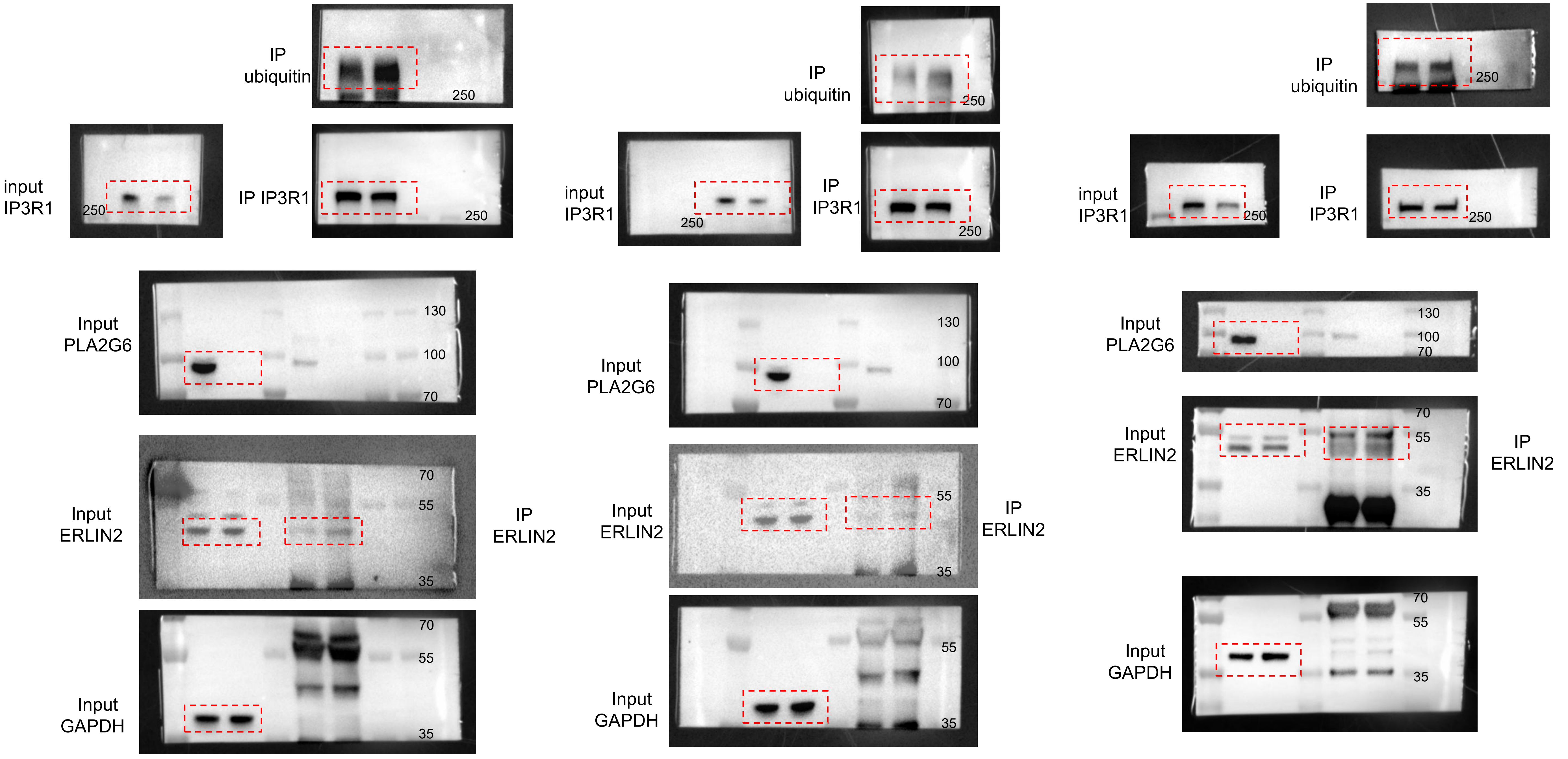

Fig. S1a

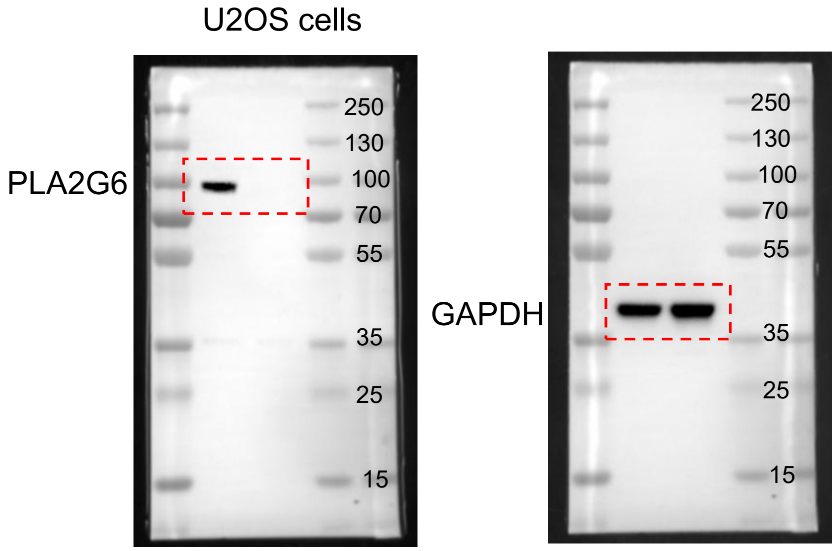

Fig. S1b

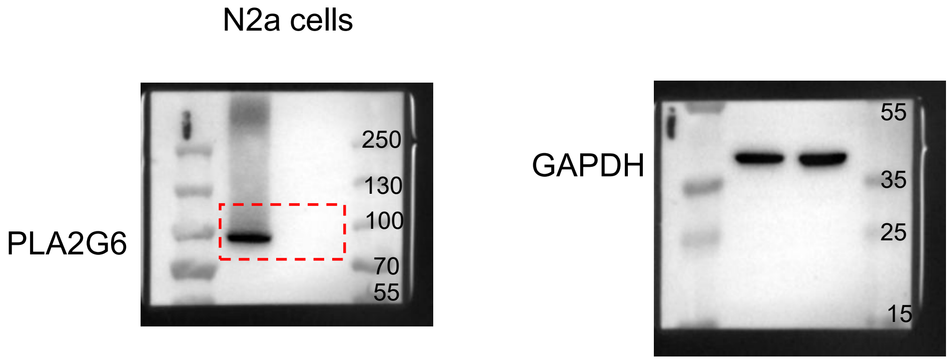

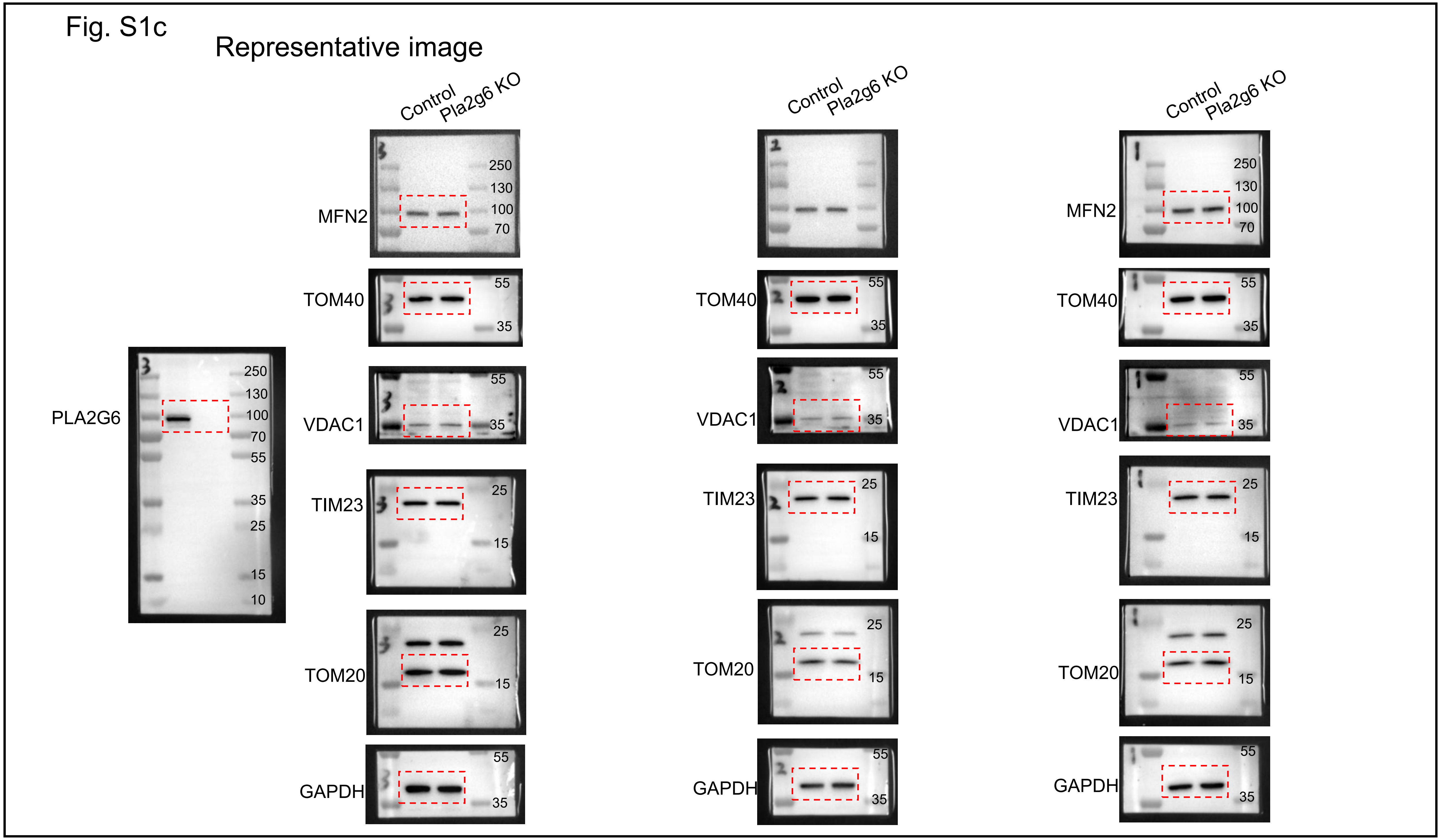

Fig. S1c
Representative image
Control
Pla2g6 KO
PLA2G6
MFN2
TOM40
VDAC1
TIM23
TOM20
GAPDH
250
130
100
70
55
35
25
15
10

Fig. S5e

Representative image

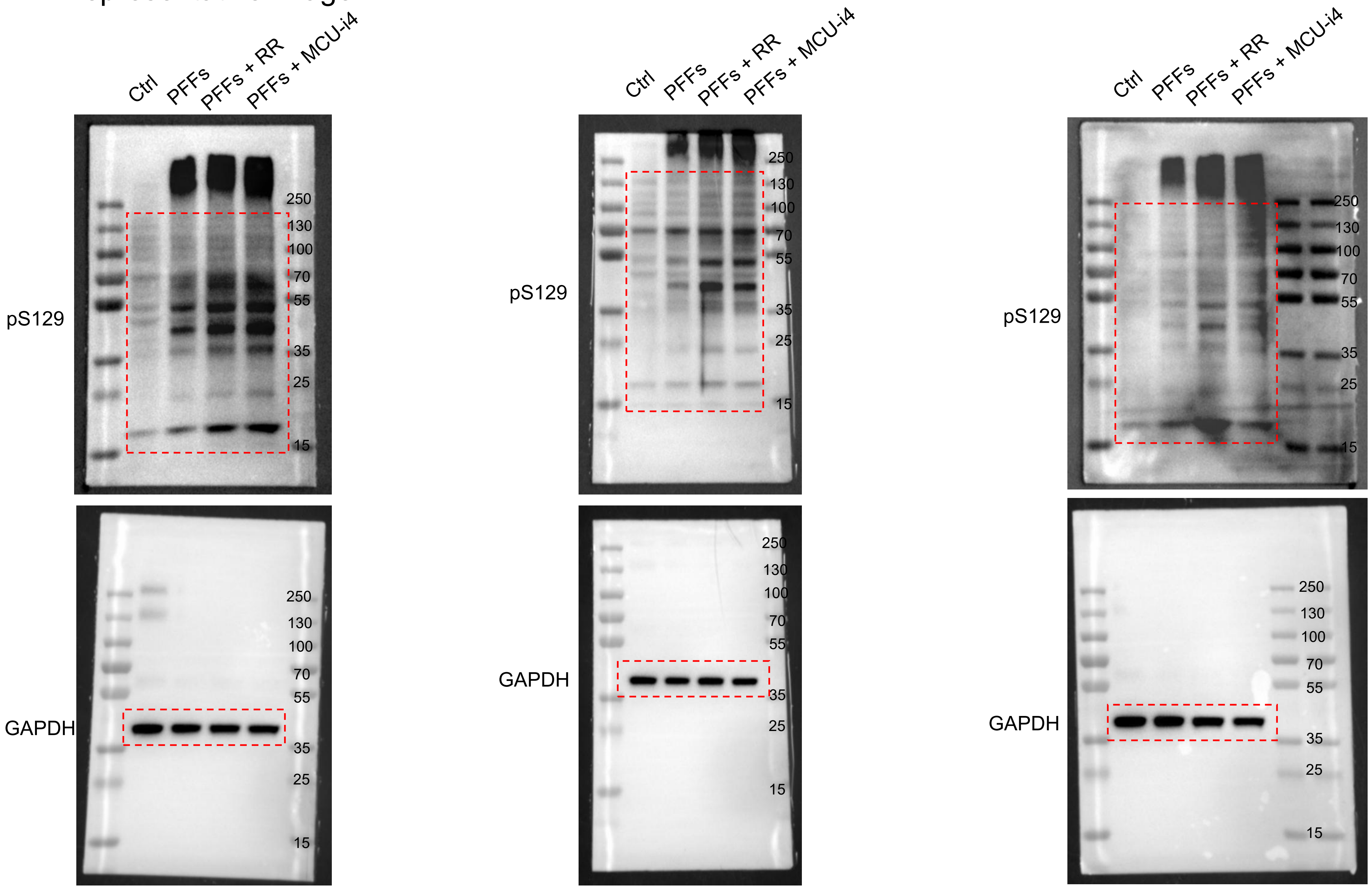

Fig. S7a

Representative image

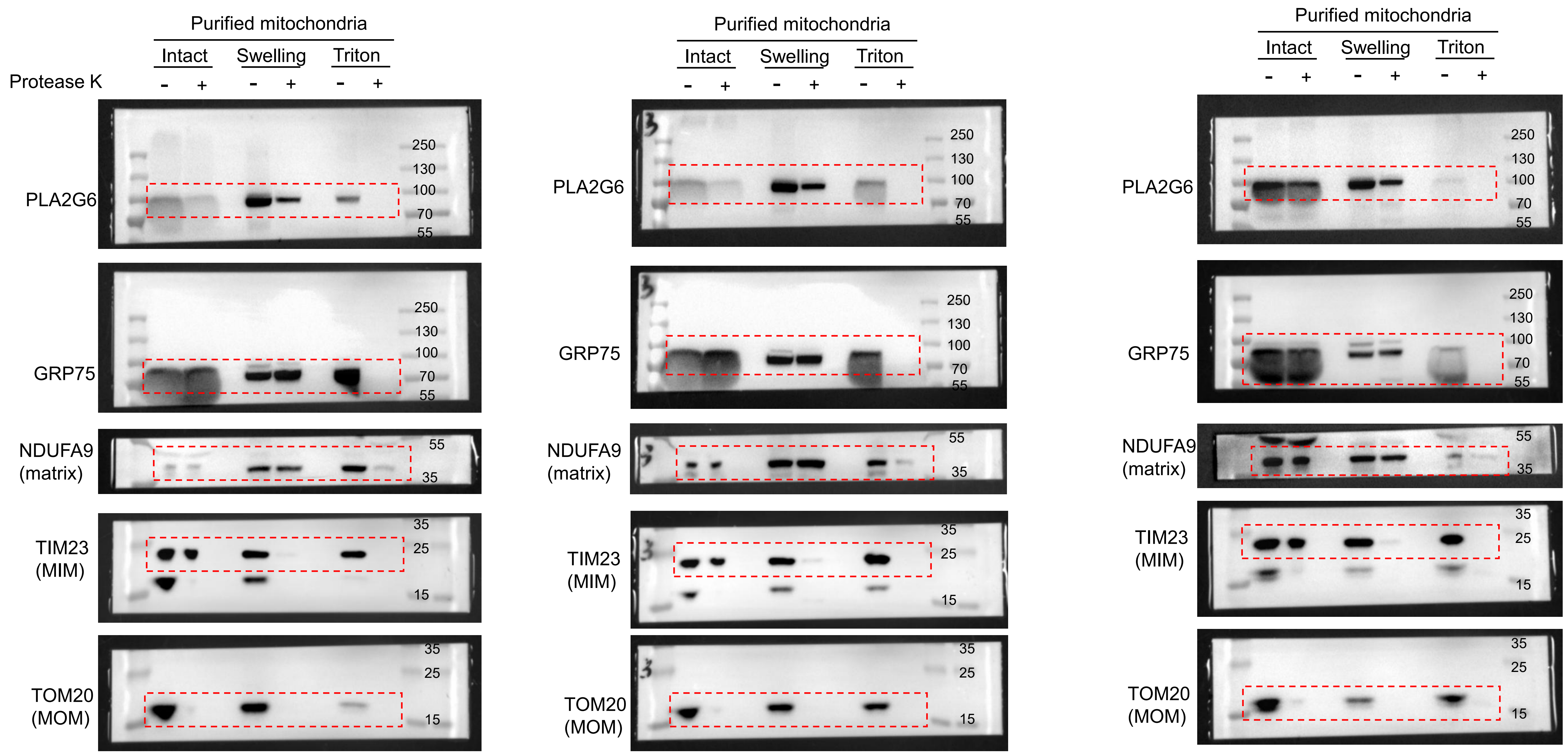

Fig. S7b

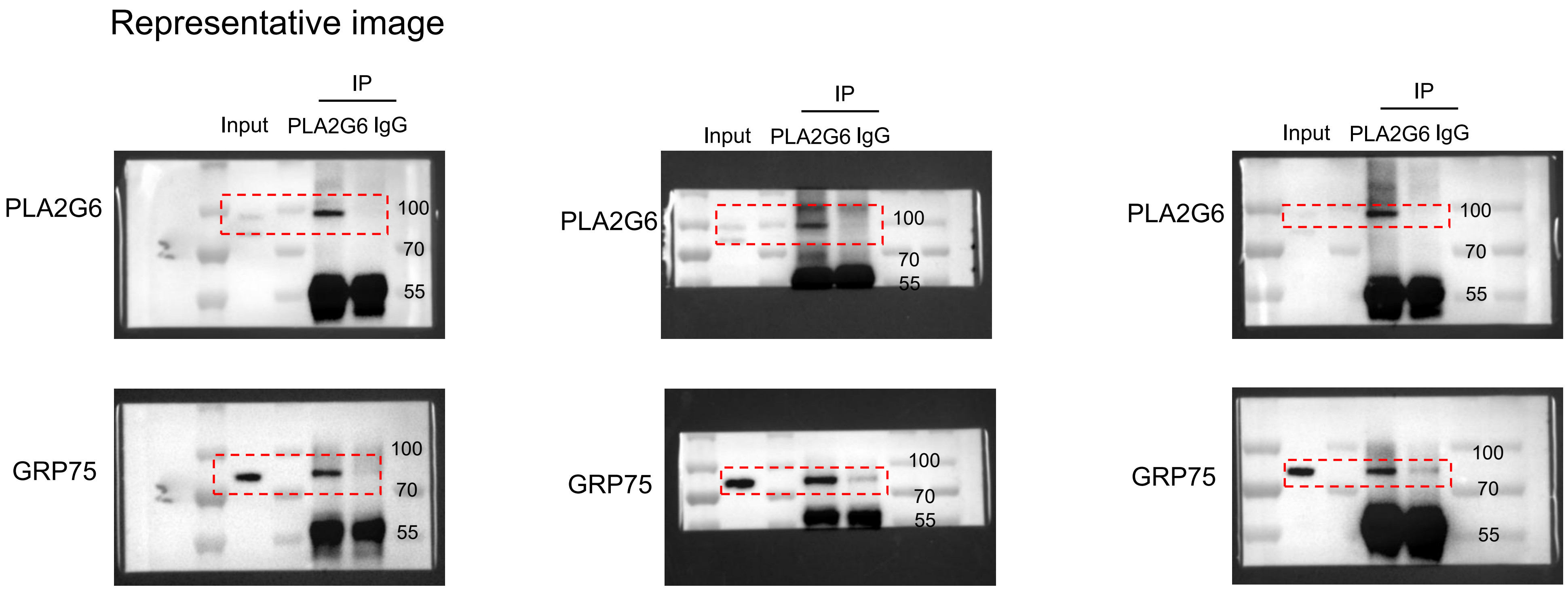
Representative image
IP
Input
PLA2G6
IgG
PLA2G6
GRP75
100
70
55

Fig. S7c

Representative image

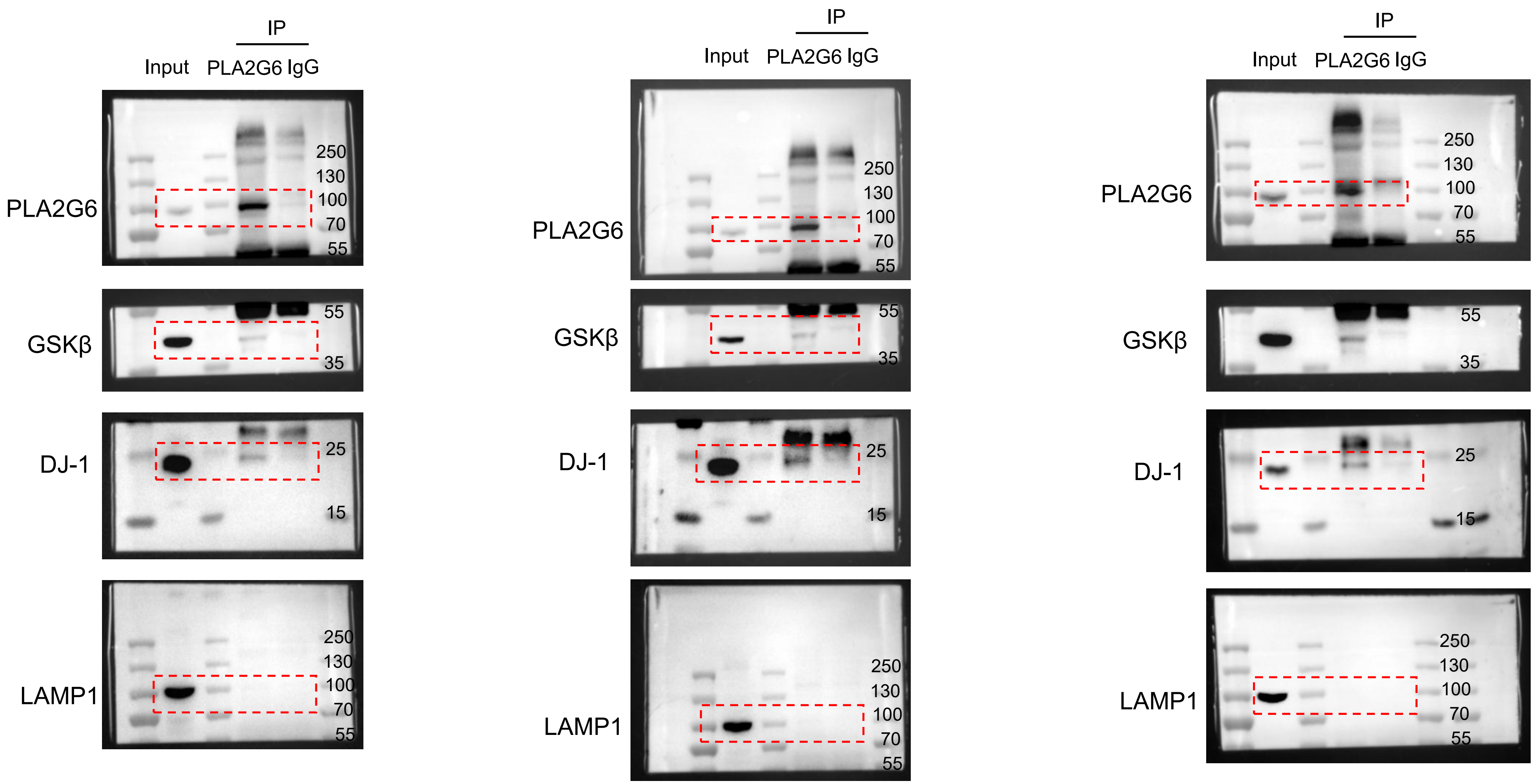

Fig. S8i

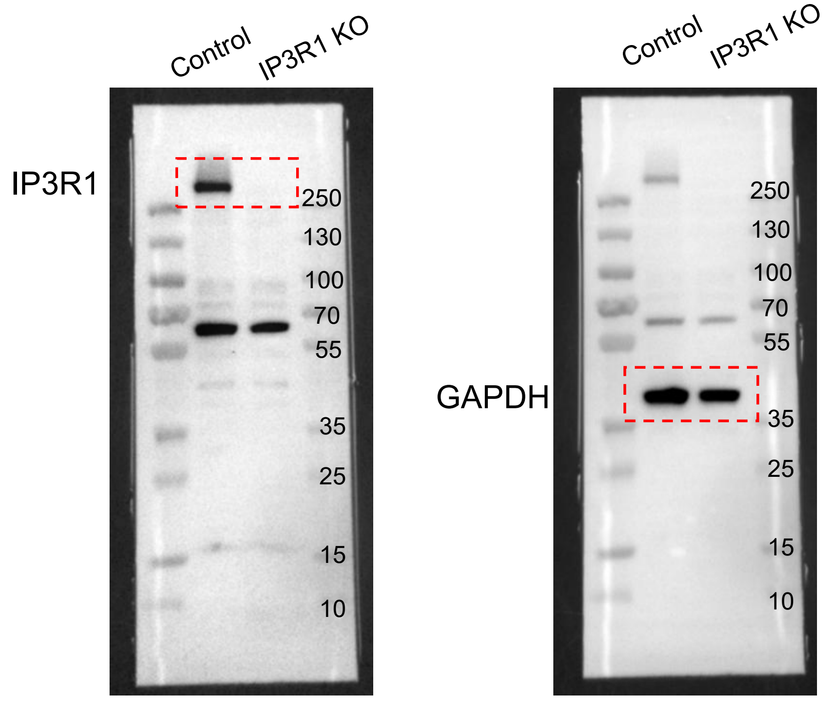

Fig. S9a

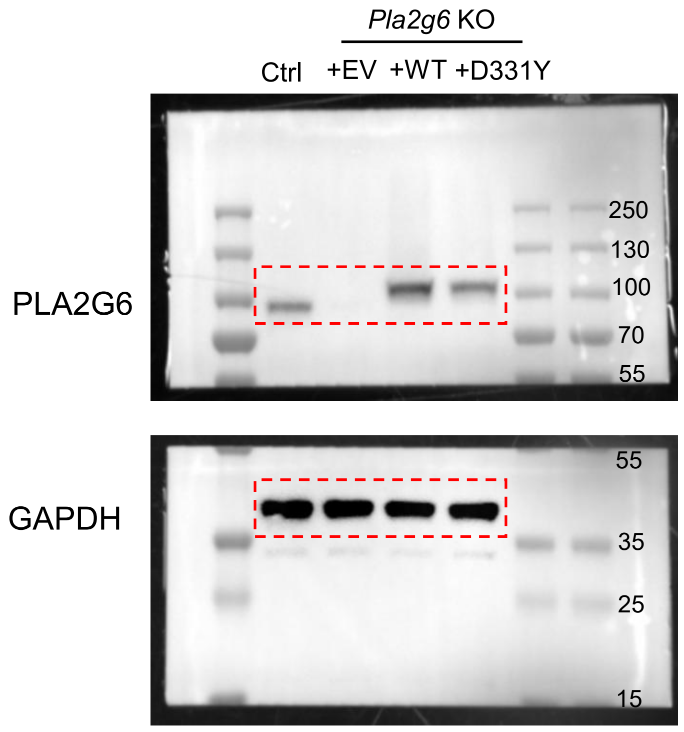

Fig. S9b

Representative image

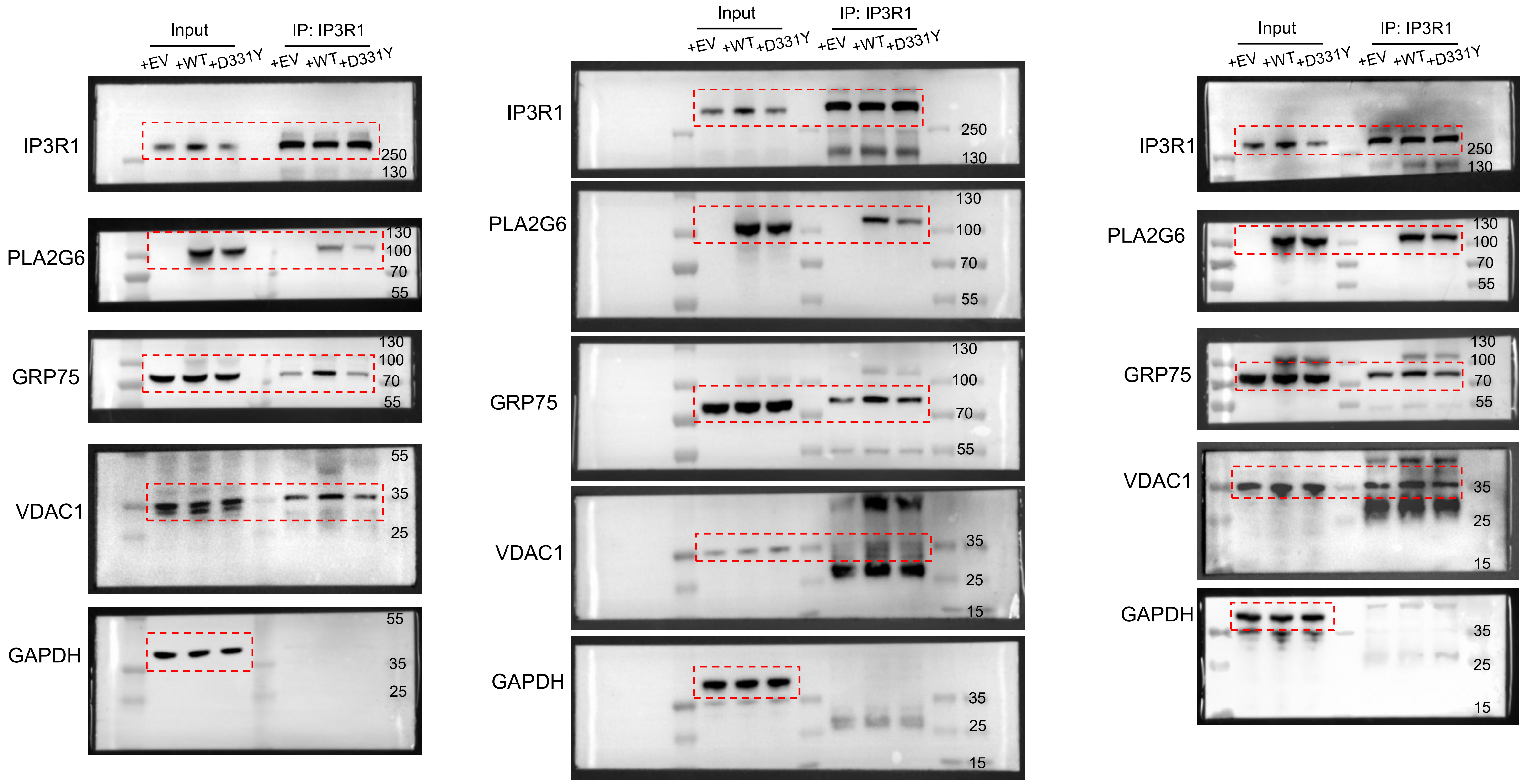

Fig. S10a

Representative image

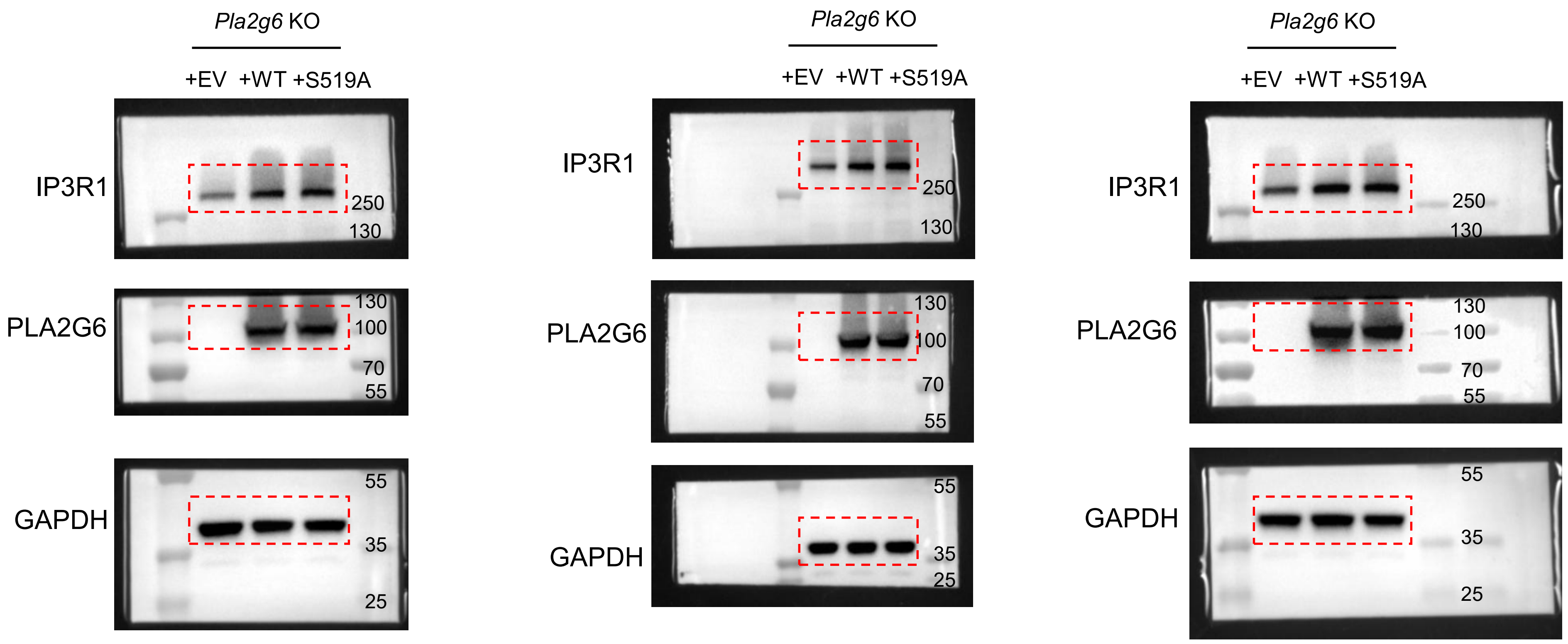

Fig. S10e

Representative image

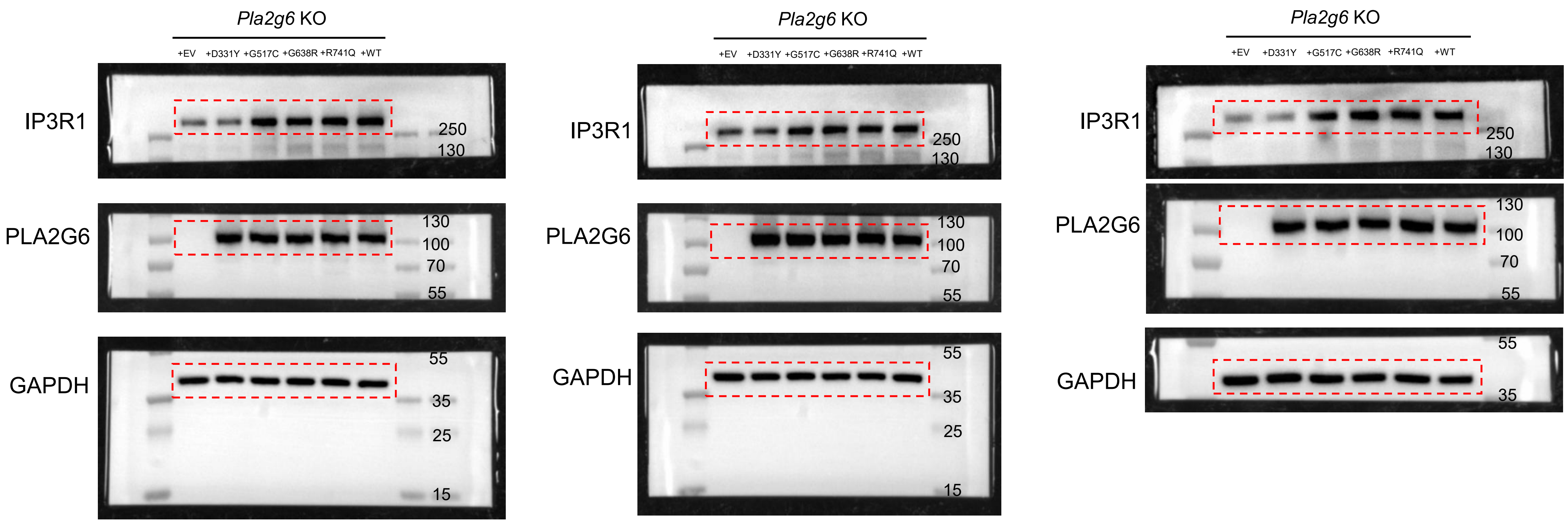

Fig. S10g

Representative image

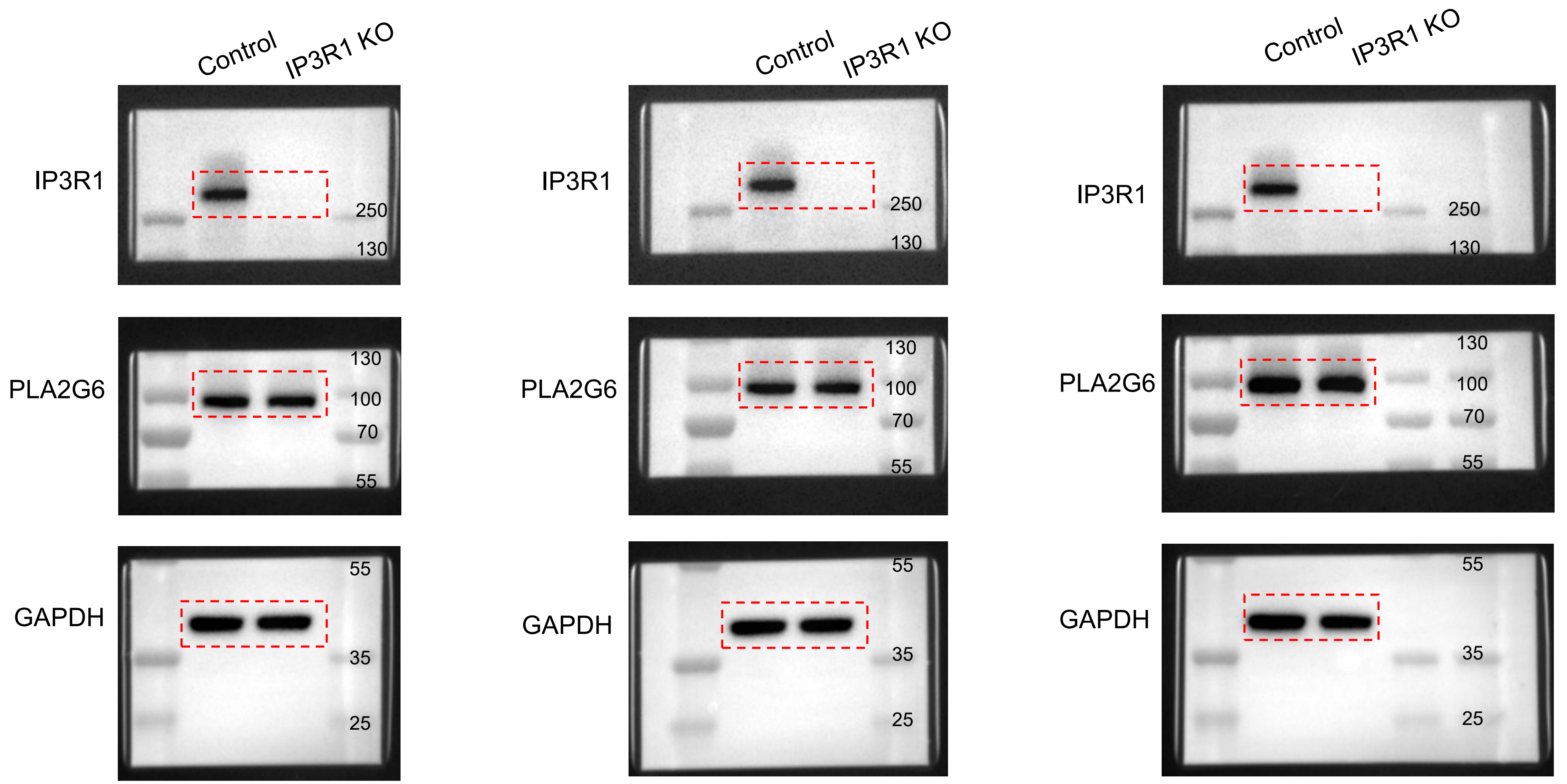

Fig. S10i

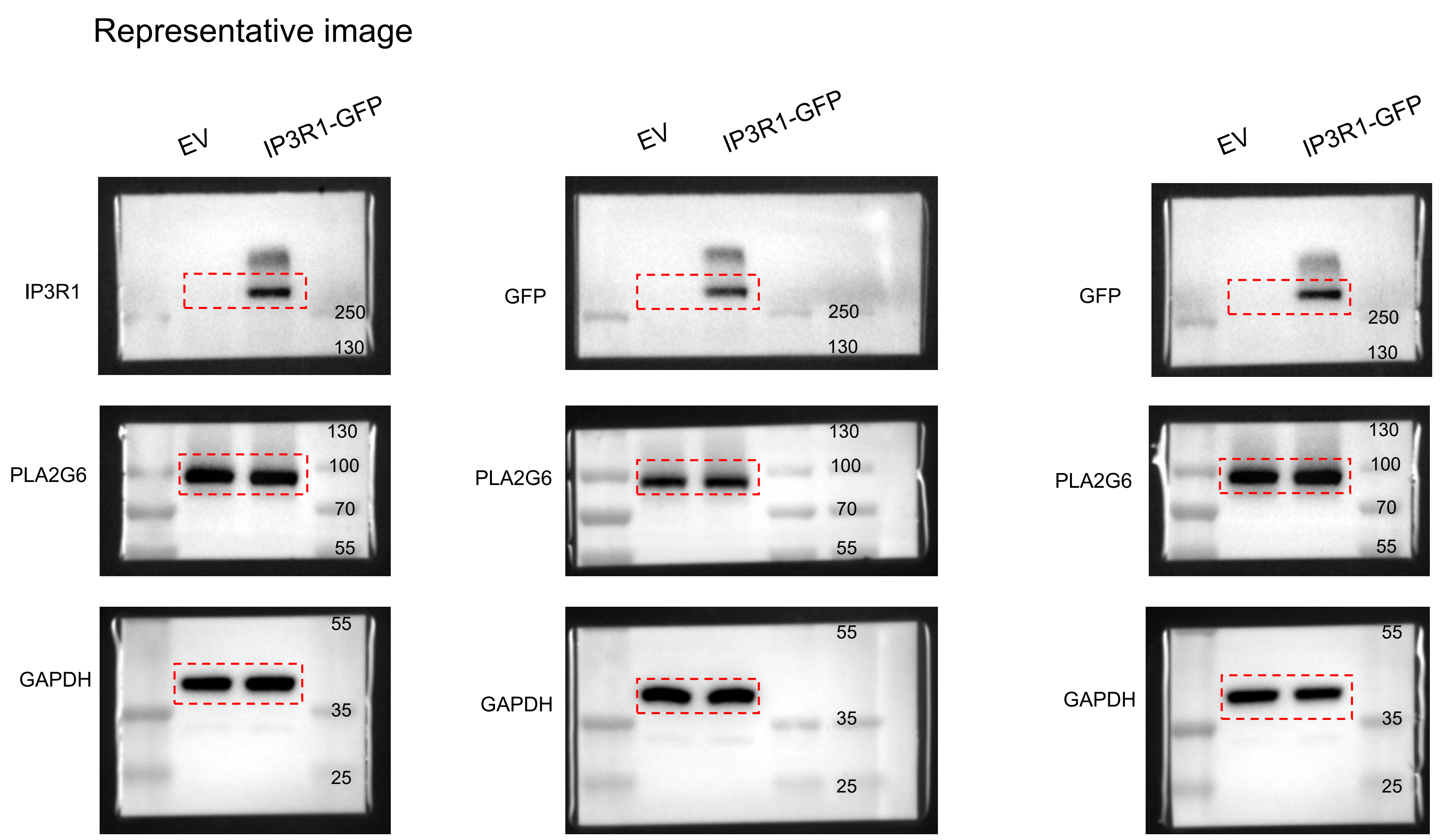
Representative image
EV
IP3R1-GFP
IP3R1
GFP
PLA2G6
GAPDH
250
130
100
70
55
35
25

Fig. S10k

Representative image

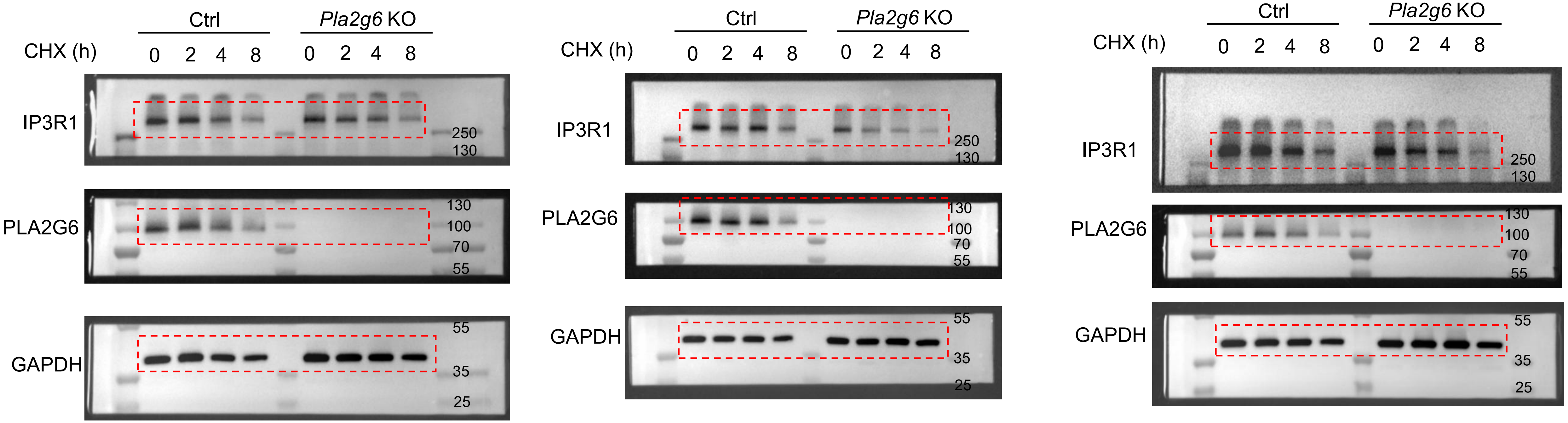

Fig. S10m

Representative image

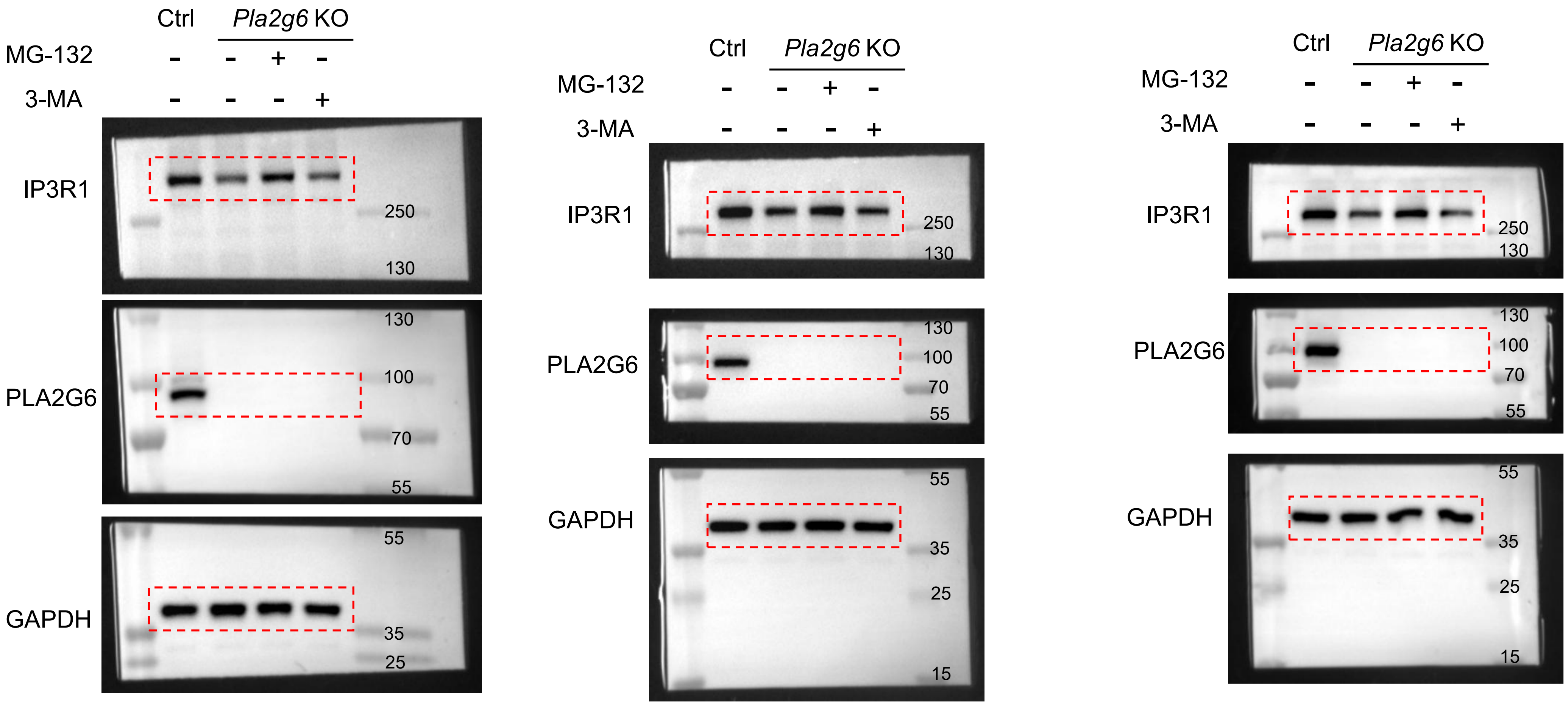

Fig. S13a

Representative image

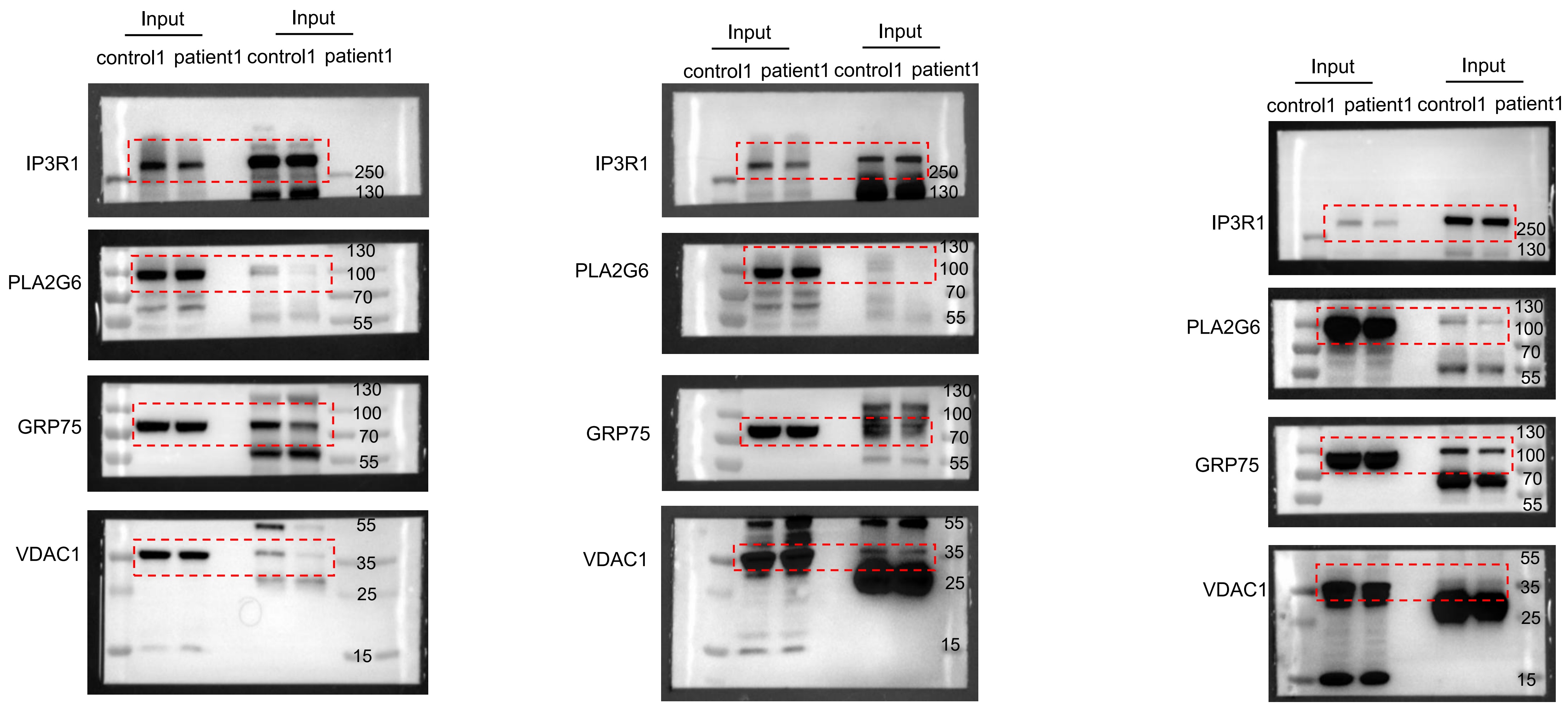

Fig. S13c

Representative image

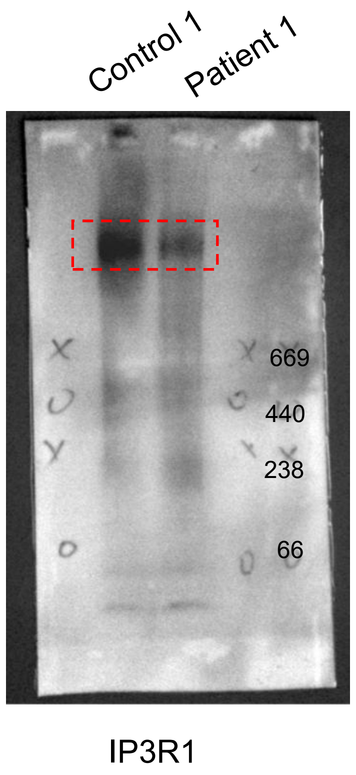

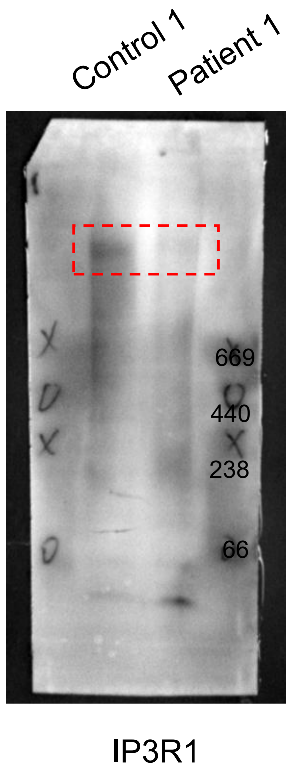

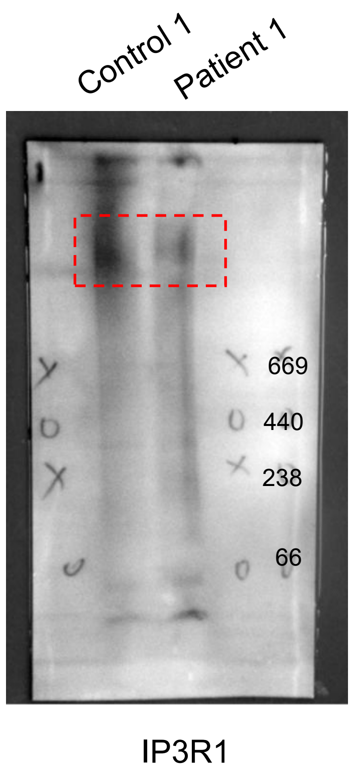

Supplement: Supplementary file 7 — Source data [file 41467_2026_70752_MOESM7_ESM.zip › Source data/Western Blotting_uncropped blots.pdf]
