# Supplementary material for: Parkinson’s disease-associated PLA2G6 protects IP3R1 protein to control ER-mitochondria tethering and Ca2+ transfer
Source: Nat Commun. 2026 Mar 19;17:5338. doi: 10.1038/s41467-026-70752-1 (PMC13272647; doi:10.1038/s41467-026-70752-1)
Supplement: Supplementary file 6 — Transparent Peer Review file [file 41467_2026_70752_MOESM6_ESM.pdf]

nature portfolio

Peer Review File

Parkinson's disease-associated PLA2G6 protects IP3R1 protein to control ER-mitochondria tethering and Ca2+ transfer

Corresponding Author: Professor Baorong Zhang

This file contains all reviewer reports in order by version, followed by all author rebuttals in order by version.

Version 0:

Reviewer comments:

Reviewer #1

(Remarks to the Author)
In this manuscript, the authors present evidence suggesting that PLA2G6 is a component of the IP3R1-GRP75-VDAC1 complex and is localized to the ER-mitochondria contact sites (MAMs). They propose that PLA2G6 may play a role in regulating the transfer of Ca2+ from the ER to mitochondria, highlighting its potential involvement in MAM function. They further demonstrate that loss of PLA2G6 leads to degradation of IP3R1 and destabilizes the IP3R1-GRP75-VDAC1 complex, which in turn disrupts ER-to-mitochondria Ca2+ transfer. This model is further supported by experiments in iPSC-derived dopaminergic neurons from Parkinson’s disease patients harboring PLA2G6 variants.
The manuscript's primary strength lies in its report of a potential interaction between PLA2G6 and the IP3R1-GRP75-VDAC1 complex. However, several major concerns affect the novelty and robustness of their conclusions. The manuscript does not clearly establish a causal link between the observed reduction in MAM length and the disruption of mitochondrial/ER morphology and functon(See 1). Moreover, the evidence suggesting that PLA2G6 loss leads to a reduction in MAM length is not compelling (see 2). Furthermore, while PLA2G6 is in the IP3R1-GRP75-VDAC1 complex, the authors do not address PLA2G6’s role in maintaining MAM integrity. Since the authors' model hinges on these three points, the absence of critical supporting data, along with several other issues (outlined below), raises many issues. Therefore, the manuscript is not ready for publication. Here is a detailed list of the specific concerns.
1. Defects in PLA2G6 have been widely associated with ER and mitochondrial abnormalities across various models, including flies, cultured cells and iPSC-derived dopaminergic neurons. In this manuscript, the authors propose that loss of PLA2G6 disrupts MAMs. However, a key issue is that the structural abnormalities observed in MAMs could be a secondary consequence of changes to the ER and/or mitochondria. To assess this, it is essential to investigate the role of PLA2G6 in both the ER and mitochondria, examine potential alterations in structure (e.g., area or length) and function. For instance, the observed reduction in mitochondrial Ca2+ transfer could result from impaired Ca2+ release from the ER or reduced mitochondrial Ca2+ uptake.
2. In Fig. 2d, the change in "Length of each MAM" between control and KO cells is subtle, and only two sample points are different in the control group. Similarly, the "Size of each MAM" (Fig. 2g) also has a subtle reduction. These small differences raise concerns about the robustness of the MAM phenotype and its role in the Ca2+ transfer in PLA2G6 KO cells.
3. Building on point 2, the "MAM length/mitochondrial perimeter (%)" (Fig. 2e) and "MAM surface/mitochondrial surface (%)" (Fig. 2h) show a more pronounced reduction than the "Length of each MAM" (Fig. 2d) and "Size of each MAM" (Fig. 2g). This suggests that the MAM defect is primarily driven by the expansion of the mitochondrial perimeter, rather than changes in the length of individual MAMs. These findings further support the concern that the observed MAM defects are secondary to broader ER and mitochondrial abnormalities.
4. Figures 1a-c show that PLA2G6 is not only present in MAM, but predominantly found in crude mitochondrial and ER fractions. Additionally, the staining data in Fig. 1d reveals that PLA2G6 is homogeneously distributed across both the ER and mitochondria. These findings indicate that PLA2G6 is broadly localized to the ER and mitochondria, rather than being specifically enriched at the MAM. BTW, if PLA2G6 were truly localized to the MAM, one would expect puncta-like staining in Fig. 1d, which is not observed. These observations strengthen concerns that the MAM phenotypes are secondary to defects in the ER and mitochondria, thereby diminishing the novelty of the manuscript's central claim.
5. In Fig. S2, the PLA2G6 KO N2a and HEK293T cells do not exhibit the lysosome expansion phenotype. This contradicts previous reports showing expanded lysosomes in PLA2G6 KO cells, mice, and patient-derived neurons. This discrepancy raises concerns about the specificity of their KO methodology and, consequently, the robustness of their data.

6. Previous studies have shown that PLA2G6[ko/ko] mice typically exhibit detectable defects at around 1 year of age. At 3 months, no obvious mitochondrial morphological defects have been reported (PMID: 27030050). However, in Fig. 2j, the authors show obvious mitochondrial morphological defects in the dopaminergic neurons of the 3-month-old PLA2G6[ko/ko] mice. Can the authors provide an explanation for this discrepancy? Are mitochondria in dopaminergic neurons more sensitive to PLA2G6 loss?

Reviewer #2

(Remarks to the Author)
This manuscript demonstrates that PLA2G6 is localized to the mitochondria-associated membranes (MAMs) and plays a critical role in MAM maintenance, as shown in cultured cells, mouse models, and iPSC-derived neurons. Additionally, the study suggests that MAM destabilization reduces Ca2+ influx into mitochondria, resulting in mitochondrial dysfunction, as observed in cultured cells and patient-derived neurons.

As a mechanism by which PLA2G6 maintains MAM, the study reveals that the stability of IP3R1 in the IP3R1-GRP75-VDAC1 complex within the MAM is enhanced in the presence of PLA2G6. Conversely, in the pathogenic mutant D331Y, MAM stability is compromised. Upon activation, IP3R1 is ubiquitinated by ERLINs/RNF170 and subsequently degraded. The authors speculate that PLA2G6 might inhibit the binding of ERLINs/RNF170 to IP3R1, thereby stabilizing IP3R1.

The role of PLA2G6 proposed in this study is straightforward and clear. However, cells from patients with PLA2G6 mutations exhibit a variety of severe pathologies, including increased lipid membrane oxidation and enhanced aggregation of alpha-synuclein. It is highly questionable whether these severe phenotypes can be explained solely by the reduction in Ca2+ influx into mitochondria. Notably, phospholipase activity is reportedly diminished in PLA2G6 D331Y; is phospholipase activity essential for IP3R1 stabilization? If so, through what mechanism? This question could be addressed using a phospholipase-dead mutant.

Additional concerns are listed below:
• The super-resolution images show fragmented mitochondria and ER, which do not appear in normal morphology. What image processing techniques were used? The super-resolution images suggest a deviation from normal morphology, as indicated by comparison with FIB-SEM-reconstructed ER and mitochondria. Meanwhile, in Fig. 2c, the mitochondria in wild-type N2a cells appear swollen, suggesting potential fixation artifacts.
• In Fig. 2, please define the metrics for "length," "size," and "surface of MAM." Additionally, what does "the ratio of MAM length to mitochondrial perimeter" indicate?
• What evidence supports that the EM images in Fig. 2j are from dopaminergic neurons?
• In the legend of Fig. 2i, the sentence "The ER surface was quantified as the region surrounding mitochondria." is unclear.
• For Fig. 3a, where interacting proteins were identified by mass spectrometry using anti-PLA2G6 immunoprecipitates, could the authors provide the full dataset? Were IP3R1, GRP75, VDAC1, ERLIN1/2, and RNF170 detected? Was GRP75, a known sticky protein, absent from control IgG immunoprecipitates?
• In Fig. 3b, PLA2G6 and GRP75 should be detected separately to validate antibody specificity.
• In Fig. 3e and f, IP was performed on mitochondrial crude fractions, but in comparison to the results of Fig. 3b and c, GRP75's co-immunoprecipitation appears significantly reduced relative to its presence in input. What does this suggest?
• The data in Fig. 3i do not provide sufficient evidence to convincingly conclude that these proteins form a complex.
• Why are signals concentrated near the nuclei in the PLA images of Fig. 4 and Fig. S6?
• Is the observed reduction in IP3R1 expression in PLA2G6 KO consistent? Fig. S7b does not show a reduction in IP3R1. Also, why does VDAC1 appear as a doublet in some instances and a single band in others?
• If PLA2G6 regulates IP3R1 stability under basal conditions, PLA data for ERLIN2-IP3R1 and RNF170-IP3R1 without CCh stimulation would be necessary. Are data available for ERLIN1? Were cells treated with CCh in Fig. 5k and l?
Minor Points:
• Fig. 2 and Fig. S7 present redundant KO data, and the figure layout could be optimized.
• Why does the introduced PLA2G6 in Fig. S7a differ in molecular weight from the endogenous protein?
• The citations in Lines 185 and 196 referring to Fig. S6 are incorrect and should reference Fig. S5.

Reviewer #3

(Remarks to the Author)
The study explores the role of PLA2G6 in regulating ER-mitochondrial contact sites, mitochondrial function, and its implications for Parkinson's disease (PD). Employing complementary approaches such as advanced imaging techniques (HIS-SIM, TEM, FIB-SEM), biochemical fractionation, and functional assays, the study investigates PLA2G6 localization and function across in vitro and in vivo models.
The presented data are interesting and relevant.
While the relationship between PLA2G6, mitochondria, and ER-mitochondrial contact sites has been previously documented—particularly the role of PLA2G6 at mitochondria-associated membranes (MAMs) in Drosophila (PMID: 31548400)—the current study should clearly articulate how its findings extend or differ from this prior work to underscore its novel contributions. This study strengthens translational relevance by using several complementary models, including

human induced pluripotent stem cell-derived systems. To further solidify the findings, additional experiments are recommended, particularly validation of the role of the GRP75/IP3R/VDAC complex in human PD patient-derived cells. This would provide deeper insights and enhance the study's contribution to understanding PLA2G6's role in PD pathogenesis.
The authors present evidence supporting a role for PLA2G6 in regulating mitochondria-associated membrane (MAM) integrity by modulating the IP3R-GRP75-VDAC1 complex through interactions with GRP75. To strengthen the mechanistic understanding, the authors should elaborate on how IP3R1 degradation impacts calcium homeostasis and its downstream effects on neuronal function.

1. The authors propose structural and functional associations between PLA2G6 and GRP75; however, additional experimental validation is necessary to substantiate this claim. Performing co-immunoprecipitation experiments to confirm the interaction between PLA2G6 and GRP75, while including known interactors of the GRP75/IP3R/VDAC complex as controls, would provide stronger evidence and significantly enhance the robustness of these findings.
2. A detailed cellular localization analysis of PLA2G6 and GRP75 within specific ER and mitochondrial subcompartments (e.g., ER lumen, ER membrane, MAM, MOM, MIM, and mitochondrial matrix) would provide critical insights into the spatial basis of these interactions. This analysis could clarify the precise sites of functional relevance. Additionally, Figure 1d is of poor quality, and the claimed finding is not evident. The figure should be improved, with higher resolution and clear annotations, to support the stated results effectively.
3. An additional figure where IP3R/VDAC1 PLA is performed in an IP3R or VDAC1 knock-out or knock-down cell line should be included as a negative control.
4. A blot against a non-PLAG2A6 interacting protein should be included to highlight that the IP only detects PLAG2A6 interacting proteins.
5. To determine whether reduced IP3R1 levels stem from increased degradation, the authors could consider blocking degradation pathways to clarify if degradation of IP3R1 overexpression impacts PLA2G6 or if there is a reciprocal regulatory mechanism.
6. While the study emphasizes IP3R1-mediated Ca^{2+} transfer, the lack of significant changes in PKD2- or ryanodine receptor-mediated Ca^{2+} flux could be better discussed to refine PLA2G6's specificity in regulating IP3R1-dependent processes.

Minor
- The Methods section should clarify the methods used to quantify mitochondrial morphology and MAM.
- The authors should provide a more comprehensive characterization of iPSC differentiation across the different lines by including additional relevant markers for validation. Furthermore, presenting low-magnification images would help assess the overall differentiation efficiency and uniformity, thereby enhancing the rigor and clarity of the study.

Reviewer #4

(Remarks to the Author)
The authors used transmission electron microscopy (TEM) and focused ion beam-scanning electron microscopy (FIB-SEM) to observe and analyze inter-organelle contacts, which is a fascinating area of research. However, there are several aspects where I would appreciate further clarification.

Major comments:

1. FIB-SEM Imaging and Segmentation Methodology: The manuscript mentions the image processing steps of "alignment, filtering, and manual segmentation" but lacks a detailed explanation of the specific methods or algorithms employed. It is unclear whether automated segmentation algorithms were used or if the segmentation process relied on manual intervention. If manual segmentation was utilized, how was consistency ensured across different datasets? Additionally, the resolution of the images in terms of lateral (horizontal) resolution should be clarified to provide a better understanding of the imaging quality.

2. Super-resolution Imaging and Co-localization Analysis: While the manuscript describes the use of super-resolution imaging for co-localization analysis, the description of the software and the specific analysis methods employed could be strengthened. It would be helpful to provide more detail on the computational tools or algorithms used to quantify co-localization, as well as how potential issues such as background noise and image artifacts were addressed during the analysis.

3. ER-mitochondria Contact Sites: What detailed methods were used to calculate the ER-mitochondria (also including ER-lysosome and mitochondria-lysosome) contact sites? Specifically, what hyperparameters of the contact site calculator in the Amira software did the authors implement? For example, how close must two objects be to be considered a contact site? If this threshold is changed, will the results change? Multiple softwares (Amira, Imaris, ImageJ) were used. Did they use the same quantification method or criterion? Understanding the methodology behind these calculations is crucial for interpreting the results accurately.

4. The Morphology of ER: As shown in Figures 2 c and f, it seems that there are not only differences between the ER-mitochondria contact sites, but also differences between ER morphology. For example, the diameter of the tubular structure formed by the ER (or MAM).

5. Results: The article appears to focus solely on the area of contact surfaces, noting that "in Pla2g6 KO N2a cells, the size of ER-mitochondria contacts decreased by 38.5% compared to control cells (Fig. 2g)." However, Figure 2f indicates that individual mitochondria have multiple contact sites. How might the analysis of changes in the number of contact sites

contribute to the understanding of this phenomenon? Could it occur that, despite a potential reduction in the overall contact area (i.e., the sum of all contact surfaces), the area of each individual contact site may increase? This phenomenon could arise as a result of a decrease in the number of contact points. An analysis of both surface area and contact site quantity could provide deeper insights.

Minor comments:
Finally, a minor discussion point: the morphology of mitochondria is closely related to both health and disease states [1]. Do the authors anticipate that variations in mitochondrial shape could influence the contact area and the number of contact sites? Exploring this relationship may add an interesting dimension to the current study.

I look forward to the authors' responses to these queries, which I believe will enhance the clarity and depth of the manuscript.

[1] Yapa N M B, Lisnyak V, Reljic B, et al. Mitochondrial dynamics in health and disease[J]. FEBS letters, 2021, 595(8): 1184-1204.

Version 1:

Reviewer comments:

Reviewer #1

(Remarks to the Author)
There are three major concerns that remain unaddressed. The original concerns are listed under "Unaddressed Concern" sections and my new comments are listed in the "Comment on the authors’ response to this concern" section.

Unaddressed Concern 1:
ER and mitochondrial abnormalities have previously been associated with PLA2G6 loss-of-function models. Hence, the structural changes observed at the mitochondria-associated membranes (MAMs) could be secondary to broader disruptions in ER and/or mitochondrial architecture. Two unresolved issues underscore this concern. First, in the original Fig. 1d, PLA2G6 was shown to colocalize predominantly with mitochondrial markers and only partially with ER markers, rather than being specifically enriched at the MAM. This finding directly contradicts the authors’ claim of a specific role for PLA2G6 at the MAM.

Comment on the authors’ response to this concern:
To address this concern, the authors replaced the images in Fig. 1d. The original figure displayed a relatively broad distribution of PLA2G6 across mitochondria and some ER, rather than a specific enrichment at MAMs. However, in the revised images, PLA2G6 staining is confined to the MAM, with no apparent signal in the surrounding ER or mitochondria. This not only contradicts their original data but also conflicts with the published literature, raising serious concerns.

Unaddressed Concern 2:
To follow up on the previous concern, the changes presented in Fig. 2 regarding MAM structure are subtle. For example: the difference in 'Length of each MAM' between control and knockout cells is minimal, with only two data points showing variation in the control group. Similarly, the reported reduction in 'Size of each MAM' is modest. These marginal differences raise concerns about the robustness and biological relevance of the proposed MAM phenotype resulting from PLA2G6 loss.

Comment on the authors’ response to this concern:
In the revised manuscript, the authors addressed the role of MAMs in Ca^{2+} transfer. However, they did not provide a clear explanation or additional data to support a biological role for PLA2G6 in regulating MAM morphology. The reported increase in 'Length of each MAM' appears to be driven primarily by two outliers, while the majority of data points show no significant difference. Similarly, the reported reduction in 'Size of each MAM' is modest.

Unaddressed Concern 3:
Previous studies have shown that PLA2G6[ko/ko] mice typically exhibit detectable defects around 1 year of age. At 3 months, no obvious mitochondrial morphological defects have been reported (PMID: 27030050). However, in Fig. 2j, the authors show obvious mitochondrial morphological defects in the dopaminergic neurons of the 3-month-old PLA2G6[ko/ko] mice. Can the authors provide an explanation for this discrepancy? Are mitochondria in dopaminergic neurons more sensitive to PLA2G6 loss?

Comment on the authors’ response to this concern:
In this revised version, the authors also have not provided any explanation or additional data to address this.

In summary, given these concerns, which significantly compromise the conclusions and biological relevance of their arguments, I recommend rejection.

Reviewer #2

(Remarks to the Author)
The revised manuscript presents significantly improved organelle imaging, which in turn enhances the persuasiveness of the authors' claims.

An important new finding in this revision is that the lipase activity of PLA2G6 does not appear to be related to the phenotypes observed by the authors. Due to the lack of definitive information on the physiological substrates of PLA2G6, the relationship between its lipase activity and disease-associated mutations remains unclear. Although previous reports have assessed lipase activity of disease-associated mutants using artificial substrates, the correlation between missense mutations and enzymatic activity has been inconsistent (PMID: 20886109, PMID: 21700586). To clarify the relationship between IP3R1 stability and the disease relevance, this reviewer recommends that the authors investigate the correlation between the observed Ca^{2+} dynamics (and/or IP3R1 levels) and several known pathogenic PLA2G6 variants. In particular, it would be valuable to determine whether there are differences between INAD/NBIA-related mutations and PD-associated mutations. Furthermore, clarifying which specific domain(s) of PLA2G6 are essential for MAM formation would substantially improve the quality of the manuscript.

Below are some minor points:
•Regarding the raw data for the mass spectrometry analysis, I was able to confirm the Excel dataset included in this manuscript; however, I could not locate PXD057620 in the PRIDE website.

•In the legend of Fig. S6, it states "n = 3 independent experiments." Could the authors clarify whether this refers to representative results from three independent experiments?

•In the legend of Fig. S6b, it reads "Immunoblotting analysis of GRP7." Please confirm whether this is a typographical error.

Reviewer #3

(Remarks to the Author)
The Authors have address all our previous concerns.

Reviewer #4

(Remarks to the Author)
I appreciate the authors' efforts in revising the manuscript. The additional technical details and discussions adequately address my concerns raised in the previous round. I find the current version improved and more complete. I have no further major comments.

Version 2:

Reviewer comments:

Reviewer #1

(Remarks to the Author)
The authors have addressed my concerns,

Reviewer #2

(Remarks to the Author)
The authors have made efforts to elucidate the correlation between pathogenic variants of PD and NBIA and Ca^{2+} dynamics, and this reviewer believes the overall quality of the manuscript has improved as a result. However, in Figure S3c–f, it remains impossible to compare the calcium dynamics of each mutant with those of wild-type PLA2G6. In other words, a "KO + WT" experimental group should have been included within the same experimental system. Considering the potential effects of disease-associated variants, it is entirely plausible that the results could show "partially impaired" rather than completely abolished function.

Version 3:

Reviewer comments:

Reviewer #2

(Remarks to the Author)
All concerns have been resolved.

Open Access This Peer Review File is licensed under a Creative Commons Attribution 4.0 International License, which permits use, sharing, adaptation, distribution and reproduction in any medium or format, as long as you give appropriate credit to the original author(s) and the source, provide a link to the Creative Commons license, and indicate if changes were made.
In cases where reviewers are anonymous, credit should be given to 'Anonymous Referee' and the source.
The images or other third party material in this Peer Review File are included in the article's Creative Commons license, unless indicated otherwise in a credit line to the material. If material is not included in the article's Creative Commons license and your intended use is not permitted by statutory regulation or exceeds the permitted use, you will need to obtain permission directly from the copyright holder.
To view a copy of this license, visit https://creativecommons.org/licenses/by/4.0/

Reviewer #1 (Remarks to the Author):

In this manuscript, the authors present evidence suggesting that PLA2G6 is a component of the IP3R1-GRP75-VDAC1 complex and is localized to the ER-mitochondria contact sites (MAMs). They propose that PLA2G6 may play a role in regulating the transfer of Ca2+ from the ER to mitochondria, highlighting its potential involvement in MAM function. They further demonstrate that loss of PLA2G6 leads to degradation of IP3R1 and destabilizes the IP3R1-GRP75-VDAC1 complex, which in turn disrupts ER-to-mitochondria Ca2+ transfer. This model is further supported by experiments in iPSC-derived dopaminergic neurons from Parkinson's disease patients harboring PLA2G6 variants.

The manuscript's primary strength lies in its report of a potential interaction between PLA2G6 and the IP3R1-GRP75-VDAC1 complex. However, several major concerns affect the novelty and robustness of their conclusions. The manuscript does not clearly establish a causal link between the observed reduction in MAM length and the disruption of mitochondrial/ER morphology and functon(See 1). Moreover, the evidence suggesting that PLA2G6 loss leads to a reduction in MAM length is not compelling (see 2). Furthermore, while PLA2G6 is in the IP3R1-GRP75-VDAC1 complex, the authors do not address PLA2G6's role in maintaining MAM integrity. Since the authors' model hinges on these three points, the absence of critical supporting data, along with several other issues (outlined below), raises many issues. Therefore, the manuscript is not ready for publication. Here is a detailed list of the specific concerns.

1. Defects in PLA2G6 have been widely associated with ER and mitochondrial abnormalities across various models, including flies, cultured cells and iPSC-derived dopaminergic neurons. In this manuscript, the authors propose that loss of PLA2G6 disrupts MAMs. However, a key issue is that the structural abnormalities observed in MAMs could be a secondary consequence of changes to the ER and/or mitochondria. To assess this, it is essential to investigate the role of PLA2G6 in both the ER and mitochondria, examine potential alterations in structure (e.g., area or length) and function. For instance, the observed reduction in mitochondrial Ca2+ transfer could result from impaired Ca2+ release from the ER or reduced mitochondrial Ca2+ uptake.

2. In Fig. 2d, the change in "Length of each MAM" between control and KO cells is subtle, and only two sample points are different in the control group. Similarly, the "Size of each MAM" (Fig. 2g) also has a subtle reduction. These small differences raise concerns about the robustness of the MAM phenotype and its role in the Ca2+ transfer in PLA2G6 KO cells.

3. Building on point 2, the "MAM length/mitochondrial perimeter (%)" (Fig. 2e) and "MAM surface/mitochondrial surface (%)" (Fig. 2h) show a more pronounced reduction than the "Length of each MAM" (Fig. 2d) and "Size of each MAM" (Fig. 2g). This suggests that the MAM defect is primarily driven by the expansion of the mitochondrial perimeter, rather than changes in the length of individual MAMs. These findings further support the concern that the observed MAM defects are secondary to broader ER and mitochondrial abnormalities.

4. Figures 1a-c show that PLA2G6 is not only present in MAM, but predominantly found in crude mitochondrial and ER fractions. Additionally, the staining data in Fig. 1d reveals that PLA2G6 is homogeneously distributed across both the ER and mitochondria. These findings indicate that PLA2G6 is broadly localized to the ER and mitochondria, rather than being specifically enriched at the MAM. BTW, if PLA2G6 were truly localized to the MAM, one would expect puncta-like staining in Fig. 1d, which is not observed. These observations strengthen concerns that the MAM phenotypes are secondary to defects in the ER and mitochondria, thereby diminishing the novelty

of the manuscript's central claim.
5. In Fig. S2, the PLA2G6 KO N2a and HEK293T cells do not exhibit the lysosome expansion phenotype. This contradicts previous reports showing expanded lysosomes in PLA2G6 KO cells, mice, and patient-derived neurons. This discrepancy raises concerns about the specificity of their KO methodology and, consequently, the robustness of their data.
6. Previous studies have shown that PLA2G6[ko/ko] mice typically exhibit detectable defects at around 1 year of age. At 3 months, no obvious mitochondrial morphological defects have been reported (PMID: 27030050). However, in Fig. 2j, the authors show obvious mitochondrial morphological defects in the dopaminergic neurons of the 3-month-old PLA2G6[ko/ko] mice. Can the authors provide an explanation for this discrepancy? Are mitochondria in dopaminergic neurons more sensitive to PLA2G6 loss?

Reviewer #2 (Remarks to the Author):
This manuscript demonstrates that PLA2G6 is localized to the mitochondria-associated membranes (MAMs) and plays a critical role in MAM maintenance, as shown in cultured cells, mouse models, and iPSC-derived neurons. Additionally, the study suggests that MAM destabilization reduces Ca^{2+} influx into mitochondria, resulting in mitochondrial dysfunction, as observed in cultured cells and patient-derived neurons.

As a mechanism by which PLA2G6 maintains MAM, the study reveals that the stability of IP3R1 in the IP3R1-GRP75-VDAC1 complex within the MAM is enhanced in the presence of PLA2G6. Conversely, in the pathogenic mutant D331Y, MAM stability is compromised. Upon activation, IP3R1 is ubiquitinated by ERLINs/RNF170 and subsequently degraded. The authors speculate that PLA2G6 might inhibit the binding of ERLINs/RNF170 to IP3R1, thereby stabilizing IP3R1.

The role of PLA2G6 proposed in this study is straightforward and clear. However, cells from patients with PLA2G6 mutations exhibit a variety of severe pathologies, including increased lipid membrane oxidation and enhanced aggregation of alpha-synuclein. It is highly questionable whether these severe phenotypes can be explained solely by the reduction in Ca^{2+} influx into mitochondria. Notably, phospholipase activity is reportedly diminished in PLA2G6 D331Y; is phospholipase activity essential for IP3R1 stabilization? If so, through what mechanism? This question could be addressed using a phospholipase-dead mutant.

Additional concerns are listed below:
• The super-resolution images show fragmented mitochondria and ER, which do not appear in normal morphology. What image processing techniques were used? The super-resolution images suggest a deviation from normal morphology, as indicated by comparison with FIB-SEM-reconstructed ER and mitochondria. Meanwhile, in Fig. 2c, the mitochondria in wild-type N2a cells appear swollen, suggesting potential fixation artifacts.
• In Fig. 2, please define the metrics for "length," "size," and "surface of MAM." Additionally, what does "the ratio of MAM length to mitochondrial perimeter" indicate?
• What evidence supports that the EM images in Fig. 2j are from dopaminergic neurons?
• In the legend of Fig. 2i, the sentence "The ER surface was quantified as the region surrounding mitochondria." is unclear.

• For Fig. 3a, where interacting proteins were identified by mass spectrometry using anti-PLA2G6 immunoprecipitates, could the authors provide the full dataset? Were IP3R1, GRP75, VDAC1, ERLIN1/2, and RNF170 detected? Was GRP75, a known sticky protein, absent from control IgG immunoprecipitates?
• In Fig. 3b, PLA2G6 and GRP75 should be detected separately to validate antibody specificity.
• In Fig. 3e and f, IP was performed on mitochondrial crude fractions, but in comparison to the results of Fig. 3b and c, GRP75's co-immunoprecipitation appears significantly reduced relative to its presence in input. What does this suggest?
• The data in Fig. 3i do not provide sufficient evidence to convincingly conclude that these proteins form a complex.
• Why are signals concentrated near the nuclei in the PLA images of Fig. 4 and Fig. S6?
• Is the observed reduction in IP3R1 expression in PLA2G6 KO consistent? Fig. S7b does not show a reduction in IP3R1. Also, why does VDAC1 appear as a doublet in some instances and a single band in others?
• If PLA2G6 regulates IP3R1 stability under basal conditions, PLA data for ERLIN2-IP3R1 and RNF170-IP3R1 without CCh stimulation would be necessary. Are data available for ERLIN1? Were cells treated with CCh in Fig. 5k and l?
Minor Points:
• Fig. 2 and Fig. S7 present redundant KO data, and the figure layout could be optimized.
• Why does the introduced PLA2G6 in Fig. S7a differ in molecular weight from the endogenous protein?
• The citations in Lines 185 and 196 referring to Fig. S6 are incorrect and should reference Fig. S5.

Reviewer #3 (Remarks to the Author):
The study explores the role of PLA2G6 in regulating ER-mitochondrial contact sites, mitochondrial function, and its implications for Parkinson's disease (PD). Employing complementary approaches such as advanced imaging techniques (HIS-SIM, TEM, FIB-SEM), biochemical fractionation, and functional assays, the study investigates PLA2G6 localization and function across in vitro and in vivo models.
The presented data are interesting and relevant.
While the relationship between PLA2G6, mitochondria, and ER-mitochondrial contact sites has been previously documented—particularly the role of PLA2G6 at mitochondria-associated membranes (MAMs) in Drosophila (PMID: 31548400)—the current study should clearly articulate how its findings extend or differ from this prior work to underscore its novel contributions. This study strengthens translational relevance by using several complementary models, including human induced pluripotent stem cell-derived systems. To further solidify the findings, additional experiments are recommended, particularly validation of the role of the GRP75/IP3R/VDAC complex in human PD patient-derived cells. This would provide deeper insights and enhance the study’s contribution to understanding PLA2G6’s role in PD pathogenesis.
The authors present evidence supporting a role for PLA2G6 in regulating mitochondria-associated membrane (MAM) integrity by modulating the IP3R-GRP75-VDAC1 complex through interactions with GRP75. To strengthen the mechanistic understanding, the authors should elaborate on how IP3R1 degradation impacts calcium homeostasis and its downstream effects on neuronal function.
1. The authors propose structural and functional associations between PLA2G6 and GRP75;

however, additional experimental validation is necessary to substantiate this claim. Performing co-immunoprecipitation experiments to confirm the interaction between PLA2G6 and GRP75, while including known interactors of the GRP75/IP3R/VDAC complex as controls, would provide stronger evidence and significantly enhance the robustness of these findings.
2. A detailed cellular localization analysis of PLA2G6 and GRP75 within specific ER and mitochondrial subcompartments (e.g., ER lumen, ER membrane, MAM, MOM, MIM, and mitochondrial matrix) would provide critical insights into the spatial basis of these interactions. This analysis could clarify the precise sites of functional relevance. Additionally, Figure 1d is of poor quality, and the claimed finding is not evident. The figure should be improved, with higher resolution and clear annotations, to support the stated results effectively.
3. An additional figure where IP3R/VDAC1 PLA is performed in an IP3R or VDAC1 knock-out or knock-down cell line should be included as a negative control.
4. A blot against a non-PLAG2A6 interacting protein should be included to highlight that the IP only detects PLAG2A6 interacting proteins.
5. To determine whether reduced IP3R1 levels stem from increased degradation, the authors could consider blocking degradation pathways to clarify if degradation of IP3R1 overexpression impacts PLA2G6 or if there is a reciprocal regulatory mechanism.
6. While the study emphasizes IP3R1-mediated Ca^{2+} transfer, the lack of significant changes in PKD2- or ryanodine receptor-mediated Ca^{2+} flux could be better discussed to refine PLA2G6's specificity in regulating IP3R1-dependent processes.

Minor

- The Methods section should clarify the methods used to quantify mitochondrial morphology and MAM.
- The authors should provide a more comprehensive characterization of iPSC differentiation across the different lines by including additional relevant markers for validation. Furthermore, presenting low-magnification images would help assess the overall differentiation efficiency and uniformity, thereby enhancing the rigor and clarity of the study.

Reviewer #4 (Remarks to the Author):

The authors used transmission electron microscopy (TEM) and focused ion beam-scanning electron microscopy (FIB-SEM) to observe and analyze inter-organelle contacts, which is a fascinating area of research. However, there are several aspects where I would appreciate further clarification.

Major comments:

1. FIB-SEM Imaging and Segmentation Methodology: The manuscript mentions the image processing steps of "alignment, filtering, and manual segmentation" but lacks a detailed explanation of the specific methods or algorithms employed. It is unclear whether automated segmentation algorithms were used or if the segmentation process relied on manual intervention. If manual segmentation was utilized, how was consistency ensured across different datasets? Additionally, the resolution of the images in terms of lateral (horizontal) resolution should be clarified to provide a better understanding of the imaging quality.
2. Super-resolution Imaging and Co-localization Analysis: While the manuscript describes the use of super-resolution imaging for co-localization analysis, the description of the software and the specific analysis methods employed could be strengthened. It would be helpful to provide more

detail on the computational tools or algorithms used to quantify co-localization, as well as how potential issues such as background noise and image artifacts were addressed during the analysis.
3. ER-mitochondria Contact Sites: What detailed methods were used to calculate the ER-mitochondria (also including ER-lysosome and mitochondria-lysosome) contact sites? Specifically, what hyperparameters of the contact site calculator in the Amira software did the authors implement? For example, how close must two objects be to be considered a contact site? If this threshold is changed, will the results change? Multiple softwares (Amira, Imaris, ImageJ) were used. Did they use the same quantification method or criterion? Understanding the methodology behind these calculations is crucial for interpreting the results accurately.
4. The Morphology of ER: As shown in Figures 2 c and f, it seems that there are not only differences between the ER-mitochondria contact sites, but also differences between ER morphology. For example, the diameter of the tubular structure formed by the ER (or MAM).
5. Results: The article appears to focus solely on the area of contact surfaces, noting that "in Pla2g6 KO N2a cells, the size of ER-mitochondria contacts decreased by 38.5% compared to control cells (Fig. 2g)." However, Figure 2f indicates that individual mitochondria have multiple contact sites. How might the analysis of changes in the number of contact sites contribute to the understanding of this phenomenon? Could it occur that, despite a potential reduction in the overall contact area (i.e., the sum of all contact surfaces), the area of each individual contact site may increase? This phenomenon could arise as a result of a decrease in the number of contact points. An analysis of both surface area and contact site quantity could provide deeper insights.

Minor comments:
Finally, a minor discussion point: the morphology of mitochondria is closely related to both health and disease states [1]. Do the authors anticipate that variations in mitochondrial shape could influence the contact area and the number of contact sites? Exploring this relationship may add an interesting dimension to the current study.

I look forward to the authors' responses to these queries, which I believe will enhance the clarity and depth of the manuscript.

[1] Yapa N M B, Lisnyak V, Reljic B, et al. Mitochondrial dynamics in health and disease[J]. FEBS letters, 2021, 595(8): 1184-1204.

Dear Reviewers,

We are sincerely grateful for your invaluable comments, which have greatly enhanced the quality of this work. Enclosed with this letter are your review comments and our point-by-point responses, demonstrating our thorough consideration of all feedback received during the peer review process. The original comments appear in *italics*, with our corresponding responses in standard font and blue highlighting. The edits in the manuscript are presented in regular text with red highlights.

Thank you for your time and thoughtful review. We trust you will find our responses adequately address every concern raised, and respectfully request your continued engagement through re-evaluation of this substantially strengthened version.

Best regards,

Bao-Rong Zhang, Professor/MD
The Second Affiliated Hospital, School of Medicine, Zhejiang University.
Phone: +86-571-87784752; FAX: +86-571-87784752
Email: brzhang@zju.edu.cn

Reviewer #1 (Remarks to the Author):
In this manuscript, the authors present evidence suggesting that PLA2G6 is a component of the IP3R1-GRP75-VDAC1 complex and is localized to the ER-mitochondria contact sites (MAMs). They propose that PLA2G6 may play a role in regulating the transfer of Ca2+ from the ER to mitochondria, highlighting its potential involvement in MAM function. They further demonstrate that loss of PLA2G6 leads to degradation of IP3R1 and destabilizes the IP3R1-GRP75-VDAC1 complex, which in turn disrupts ER-to-mitochondria Ca2+ transfer. This model is further supported by experiments in iPSC-derived dopaminergic neurons from Parkinson's disease patients harboring PLA2G6 variants.
The manuscript's primary strength lies in its report of a potential interaction between PLA2G6 and the IP3R1-GRP75-VDAC1 complex. However, several major concerns affect the novelty and robustness of their conclusions. The manuscript does not clearly establish a causal link between the observed reduction in MAM length and the disruption of mitochondrial/ER morphology and functon(See 1). Moreover, the evidence suggesting that PLA2G6 loss leads to a reduction in MAM length is not compelling (see 2). Furthermore, while PLA2G6 is in the IP3R1-GRP75-VDAC1 complex, the authors do not address PLA2G6's role in maintaining MAM integrity. Since the authors' model hinges on these three points, the absence of critical supporting data, along with several other issues (outlined below), raises many issues. Therefore, the manuscript is not ready for publication. Here is a detailed list of the specific concerns.
Response: Thank you for your comment. We have responded to questions 1, 2 in detail below. Regarding question 3, we have employed multi-model systems (cell lines, animal models, iPSCs-derived dopaminergic neurons) and multiple experimental approaches (HIS-SIM, TEM, FIB-SEM) to collectively demonstrate that PLA2G6 deficiency or mutations lead to MAM disruption (Fig. 2,

6, and S2 of the revised manuscript). Furthermore, we have utilized MAM Linker to establish the causal relationship between mitochondrial morphological/functional alterations and MAM impairment in *PLA2G6* KO models (Fig. S5 of the revised manuscript). Based on this comprehensive evidence, we conclude that PLA2G6's role in maintaining MAM integrity is unequivocal.

1. Defects in PLA2G6 have been widely associated with ER and mitochondrial abnormalities across various models, including flies, cultured cells and iPSC-derived dopaminergic neurons. In this manuscript, the authors propose that loss of PLA2G6 disrupts MAMs. However, a key issue is that the structural abnormalities observed in MAMs could be a secondary consequence of changes to the ER and/or mitochondria. To assess this, it is essential to investigate the role of PLA2G6 in both the ER and mitochondria, examine potential alterations in structure (e.g., area or length) and function. For instance, the observed reduction in mitochondrial Ca2+ transfer could result from impaired Ca2+ release from the ER or reduced mitochondrial Ca2+ uptake.

Response: We appreciate your insightful comment. Prior research has demonstrated that alterations in the structure and function of MAMs significantly influence mitochondrial morphology and functions. Meanwhile, the mitochondrial morphology, but not ER morphological alterations, also affects MAM structure[1,2]. Therefore, to demonstrate that the reduction in mitochondrial Ca^{2+} transfer we observed is mainly due to the decrease in MAMs structure rather than the abnormalities in the function of the ER or mitochondria, we introduced the MAM Linker, which can promote the MAM formation. Our results showed that MAM Linker completely restores the reduction of mitochondrial Ca^{2+} transfer in *Pla2g6* KO cells (Fig. S5i, j of the revised manuscript), suggesting that the reduction in mitochondrial Ca^{2+} transfer caused by the deficiency of PLA2G6 is mainly due to the decrease of MAM. Furthermore, the enhancement of ER-mitochondria contacts mediated by MAM Linker significantly rescues mitochondrial dysfunction in PLA2G6-deficient cells, restoring mitochondrial membrane potential by 59% (Fig. S5g, h of the revised manuscript) and ATP production by 67.5% (Fig. S5k, l of the revised manuscript). This demonstrates that MAM alterations are the primary contributor to the functional abnormalities observed in mitochondria under PLA2G6 deficiency.

2. In Fig. 2d, the change in "Length of each MAM" between control and KO cells is subtle, and only two sample points are different in the control group. Similarly, the "Size of each MAM" (Fig. 2g) also has a subtle reduction. These small differences raise concerns about the robustness of the MAM phenotype and its role in the Ca2+ transfer in PLA2G6 KO cells.

Response: Thank you for your comment. To address this concern, we have demonstrated that *Pla2g6* KO led to a reduction in ER-mitochondria Ca^{2+} transfer, which can be completely rescued by MAM Linker (Fig. S5i, j of the revised manuscript). This indicates that the reduction in ER-mitochondria contact caused by *Pla2g6* KO is sufficient to cause changes in the function of the MAM, such as Ca^{2+} flux. Moreover, although no article has indicated the specific extent of MAM structural changes that would cause functional alterations in MAM, a large number of previous studies have reported that even a small amount of MAM structural change can affect Ca^{2+} transport (PMID: 27647893, 28337542, 31767755). Importantly, the average MAM length of the control group is 189.9 ± 8.9 nm, which decreased to 145.2 ± 6.6 nm in *Pla2g6* KO cells (Fig. 2d). This indicates that in the control group, the majority of the sample points are greater than those in the KO group,

rather than only two points showing a difference.

3. Building on point 2, the "MAM length/mitochondrial perimeter (%)" (Fig. 2e) and "MAM surface/mitochondrial surface (%)" (Fig. 2h) show a more pronounced reduction than the "Length of each MAM" (Fig. 2d) and "Size of each MAM" (Fig. 2g). This suggests that the MAM defect is primarily driven by the expansion of the mitochondrial perimeter, rather than changes in the length of individual MAMs. These findings further support the concern that the observed MAM defects are secondary to broader ER and mitochondrial abnormalities.

Response: Thank you for your comment. In *Pla2g6* KO N2a cells, the MAM length decreased by 23.5% (Fig. 2d of the revised manuscript), and the size decreased by 38.5% (Fig. 2g of the revised manuscript) compared to the control group. However, in *Pla2g6* KO N2a cells, the mitochondrial length only increased by 14.6% (Fig. R1A related to Fig. 2c, shown below), and mitochondrial surface only increased by 26.0% (Fig. R1B related to Fig. 2f, shown below). This suggests that *Pla2g6* KO primarily affects the MAM structure. Furthermore, we utilized MAM Linker to increase ER-mitochondrial contacts in *Pla2g6* KO cells, and we found that mitochondrial morphology was partially restored in *Pla2g6* KO cells (Fig. S5f of the revised manuscript). This suggests that mitochondrial abnormalities are secondary to MAM defects. Because the data about mitochondrial length is not the main line of our manuscript, and the length of an article in "*Nature Communications*" is also limited. Therefore, we do not provide Figure R1 in the manuscript. In addition, the absence of differences in mitochondrial surface statistics may be related to a relatively small sample size.

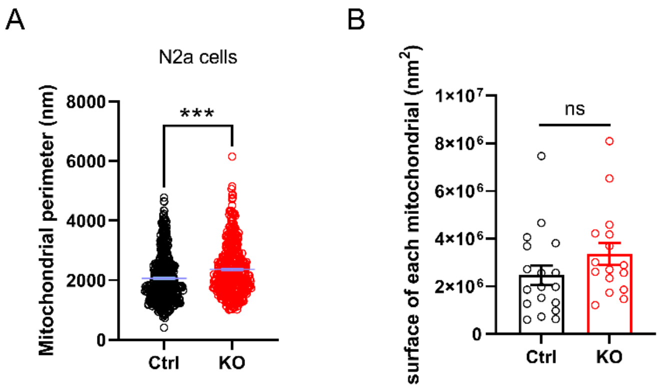

Fig. R1 Mitochondrial perimeter and surface in control and *Pla2g6* KO cells. (A) Measurement of mitochondrial perimeter in control and Pla2g6 KO cells (control, n = 492 mitochondria; KO, n = 418 mitochondria from three independent experiments). (B) Measurement of mitochondrial surface in control and Pla2g6 KO cells (control, n = 18 mitochondria; KO, n = 16 mitochondria from three independent experiments). Data are presented as means ± SEM. *** indicates statistical significance at $p < 0.001$; ns indicates not significant. Two-tailed unpaired t-tests were used for statistical analysis.

4. Figures 1a-c show that PLA2G6 is not only present in MAM, but predominantly found in crude mitochondrial and ER fractions. Additionally, the staining data in Fig. 1d reveals that PLA2G6 is homogeneously distributed across both the ER and mitochondria. These findings indicate that PLA2G6 is broadly localized to the ER and mitochondria, rather than being specifically enriched at the MAM. BTW, if PLA2G6 were truly localized to the MAM, one would expect puncta-like staining in Fig. 1d, which is not observed. These observations strengthen concerns that the MAM

phenotypes are secondary to defects in the ER and mitochondria, thereby diminishing the novelty of the manuscript's central claim.

Response: We appreciate your insightful comment. We acknowledge that PLA2G6 is not only distributed in the MAM region, but is also localized in the mitochondria and ER. In N2a cells, the PLA2G6 protein is predominantly localized in the MAM fraction (Fig. 1a of the revised manuscript). And in mouse and human brains, PLA2G6 proteins are mainly localized in the MAM and ER fractions, with less localization in pure mitochondria (crude mitochondria are composed of pure mitochondria and MAMs; Fig. 1c, d of the revised manuscript). In addition, we transiently transfected mitochondria, ER, and PLA2G6 fluorescent plasmids into U2OS cells and observed PLA2G6 localization using HIS-SIM, which showed that some puncta-like staining of PLA2G6 was localized between the ER and mitochondria (Fig. 1d, e of the revised manuscript).

5. In Fig. S2, the PLA2G6 KO N2a and HEK293T cells do not exhibit the lysosome expansion phenotype. This contradicts previous reports showing expanded lysosomes in PLA2G6 KO cells, mice, and patient-derived neurons. This discrepancy raises concerns about the specificity of their KO methodology and, consequently, the robustness of their data.

Response: We appreciate your thoughtful criticism. Since we previously focused mainly on organelle interactions and neglected the effect of *PLA2G6* KO on lysosomal morphology. After counting the lysosomal morphology in the control and KO groups, we found that, similar to previous reports[3], PLA2G6 deficiency resulted in lysosomal swelling. Therefore, we replaced the appropriate representative image (Fig. S1e, g of the revised manuscript). In addition, since the size of the N2a cell was too small, resulting in poor quality during organelle fluorescence imaging, we constructed *PLA2G6* KO U2OS cells for fluorescence experiments (Fig. S1a of the revised manuscript).

6. Previous studies have shown that PLA2G6[ko/ko] mice typically exhibit detectable defects at around 1 year of age. At 3 months, no obvious mitochondrial morphological defects have been reported (PMID: 27030050). However, in Fig. 2j, the authors show obvious mitochondrial morphological defects in the dopaminergic neurons of the 3-month-old PLA2G6[ko/ko] mice. Can the authors provide an explanation for this discrepancy? Are mitochondria in dopaminergic neurons more sensitive to PLA2G6 loss?

Response: Thank you for raising this important point. We acknowledge that we did not bother to count the mitochondrial morphology changes in 3-month-old WT and KO mice, and the representative figure we put up only takes into account the MAM changes, which may cause misunderstanding among readers. In addition, as commented by reviewer 2: What evidence supports that the EM images in Fig. 2j are from dopaminergic neurons? Although the sampling site of mice was the substantia nigra pars compacta, there is indeed no guarantee that neurons observed under electron microscopy must be dopaminergic neurons, and it is really difficult to distinguish between different neurons under electron microscopy. Therefore, to solve these two neglects, we first immunolabeled dopaminergic neurons with TH antibody, and then counted the MAM and mitochondrial length in the neurons with colloidal gold distribution. The results showed that the MAM structure has significantly decreased in dopaminergic neurons of *Pla2g6* KO mice compared to WT mice (Fig. S2d–f of the revised manuscript). However, the mitochondrial perimeter was not significantly different between the two groups (Fig. R2 related to Fig. S2d).

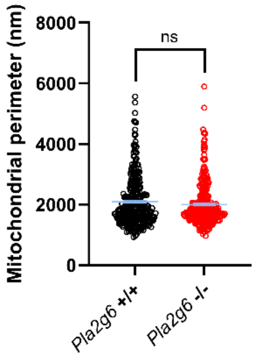

Fig. R2 Mitochondrial perimeter in dopaminergic neurons of *Pla2g6* KO and WT mice. Measurement of mitochondrial perimeter in dopaminergic neurons (WT, n = 329 mitochondria from 3 mice; KO, n = 311 from 3 mice).

Reviewer #2 (Remarks to the Author):

This manuscript demonstrates that PLA2G6 is localized to the mitochondria-associated membranes (MAMs) and plays a critical role in MAM maintenance, as shown in cultured cells, mouse models, and iPSC-derived neurons. Additionally, the study suggests that MAM destabilization reduces Ca2+ influx into mitochondria, resulting in mitochondrial dysfunction, as observed in cultured cells and patient-derived neurons.

As a mechanism by which PLA2G6 maintains MAM, the study reveals that the stability of IP3R1 in the IP3R1-GRP75-VDAC1 complex within the MAM is enhanced in the presence of PLA2G6. Conversely, in the pathogenic mutant D331Y, MAM stability is compromised. Upon activation, IP3R1 is ubiquitinated by ERLINs/RNF170 and subsequently degraded. The authors speculate that PLA2G6 might inhibit the binding of ERLINs/RNF170 to IP3R1, thereby stabilizing IP3R1.

The role of PLA2G6 proposed in this study is straightforward and clear. However, cells from patients with PLA2G6 mutations exhibit a variety of severe pathologies, including increased lipid membrane oxidation and enhanced aggregation of alpha-synuclein. It is highly questionable whether these severe phenotypes can be explained solely by the reduction in Ca2+ influx into mitochondria. Notably, phospholipase activity is reportedly diminished in PLA2G6 D331Y; is phospholipase activity essential for IP3R1 stabilization? If so, through what mechanism? This question could be addressed using a phospholipase-dead mutant.

Response: We sincerely appreciate your constructive comments. In response, we have expanded the experiments involving inhibitors of mitochondrial Ca^{2+} influx based on your comments. We found that the mitochondrial calcium uniporter (MCU) inhibitor ruthenium red and MCU-i4 did not affect lipid oxidation (Fig. S4g, h of the revised manuscript). However, the results showed that the MCU inhibitor can enhance the aggregation of α-synuclein (Fig. S4a–f of the revised manuscript).

To demonstrate if the phospholipase activity of the PLA2G6 protein is essential for IP3R1 stabilization, we re-expressed the WT PLA2G6 and catalytically impaired PLA2G6 (S519A) in *Pla2g6* KO N2a cells and showed no significant difference in IP3R1 protein expression levels between the two groups (Fig. S9a of the revised manuscript). This data suggests that the

phospholipase activity of PLA2G6 is not essential for IP3R1 stabilization. Thus, similar to previous conclusions, the phenotype caused by the PD-associated mutation in PLA2G6 was not necessarily associated with the absence of phospholipase activity[3-5].

Additional concerns are listed below:

• The super-resolution images show fragmented mitochondria and ER, which do not appear in normal morphology. What image processing techniques were used? The super-resolution images suggest a deviation from normal morphology, as indicated by comparison with FIB-SEM-reconstructed ER and mitochondria. Meanwhile, in Fig. 2c, the mitochondria in wild-type N2a cells appear swollen, suggesting potential fixation artifacts.

Response: Thank you for your comments. We processed the image using Wiener Reconstruction. The specific parameter information is as follows: defocus elimination 5, notch filter 10, 3D Wiener parameter 0.5. We also tried different parameters, but also showed fragmented mitochondria and ER. We suspected that cell membrane permeabilization might be contributing to the issue, so we reduced the permeabilization time accordingly. However, this adjustment still failed to produce the expected fluorescent images. Therefore, we directly employ live cell imaging to avoid the image influences brought about by cell fixation and membrane permeabilization. The outcomes still indicate that *PLA2G6* KO results in a decrease in the interaction between the ER and mitochondria (Fig. 2a, S1e–h of the revised manuscript). In addition, since the size of the N2a cell was too small, resulting in poor quality during organelle fluorescence imaging, we constructed *PLA2G6* KO U2OS cells for fluorescence experiments (Fig. S1a of the revised manuscript).

Regarding the second question, most mitochondria of wild-type N2a cells exhibit normal morphology. Therefore, our fixation protocol employing 2.5% glutaraldehyde at 4°C overnight does not introduce significant methodological artifacts. And we have replaced the image of abnormal mitochondria (Fig. 2c of the revised manuscript).

• In Fig. 2, please define the metrics for "length," "size," and "surface of MAM." Additionally, what does "the ratio of MAM length to mitochondrial perimeter" indicate?

Response: Thank you for your suggestions. The "size" and "surface of MAM" are the same metrics. To ease understanding, we only use the "surface of MAM" in the 3D image (Fig. 2f–h). Moreover, we have defined the metrics for "length", "surface of MAM", and "the ratio of MAM length to mitochondrial perimeter" in the Method section (lines 1057–1067 and 1090–1104).

• What evidence supports that the EM images in Fig. 2j are from dopaminergic neurons?

Response: Thank you for your valuable comment. We can distinguish neurons, microglia, and astrocytes in brain tissue based on cell morphology under electron microscopy. However, it is difficult to distinguish the different types of neurons under electron microscopy. Thus, we immunolabeled dopaminergic neurons with TH antibody, and then counted the MAM and mitochondrial length in the neurons with colloidal gold distribution. The results showed that the MAM has significantly decreased in dopaminergic neurons of *Pla2g6* KO mice compared to WT mice (Fig. S2d–f of the revised manuscript).

• The sentence "The ER surface was quantified as the region surrounding mitochondria " in the legend of Fig. 2i is unclear.

Response: Thank you for your reminder. We recognize that the definition of the ER surface is not clear and scientific enough. We have removed this metric from our article, which does not affect the article's conclusions.

• *For Fig. 3a, where interacting proteins were identified by mass spectrometry using anti-PLA2G6 immunoprecipitates, could the authors provide the full dataset? Were IP3R1, GRP75, VDAC1, ERLIN1/2, and RNF170 detected? Was GRP75, a known sticky protein, absent from control IgG immunoprecipitates?*

Response: Thank you for your comment. Candidate proteins need to fulfill the following conditions: unique peptides ≥ 2, IP abundances/ IgG abundances ≥ 10. Candidate proteins have all been listed in the Source Data. Furthermore, the raw data for mass spectrometry have been deposited to the ProteomeXchange Consortium via the PRIDE partner repository with the dataset identifier PXD057620. You can log in the PRIDE website (https://www.ebi.ac.uk/pride/), and acquire it using the following details: Project accession: PXD057620; Token: TAVUe4dRZeHz. Alternatively, you can access the dataset by logging in to the PRIDE website using the following account details: Username: reviewer_pxd057620@ebi.ac.uk; Password: Ofqi3uXwpBd4.

Regarding the questions 2 and 3, the GRP75 and VDAC1 were detected, but the IP3R1, ERLIN1/2, and RNF170 were not detected. In addition. The GRP75 is absent from control IgG immunoprecipitates.

• *In Fig. 3b, PLA2G6 and GRP75 should be detected separately to validate antibody specificity.*

Response: Thank you for your comment. We indeed detect PLA2G6 and GRP75 separately. Since their molecular weights are relatively close, we presented them together. To avoid any misunderstanding from the readers, we re-perform this experiment using 8% gel (Fig. 3b of the revised manuscript).

• *In Fig. 3e and f, IP was performed on mitochondrial crude fractions, but in comparison to the results of Fig. 3b and c, GRP75's co-immunoprecipitation appears significantly reduced relative to its presence in input. What does this suggest?*

Response: Thank you for your insightful comment. Crude mitochondria are composed of MAM and pure mitochondria fraction. As shown in Figures 1a–c, GRP75 is primarily localized to mitochondria with a minor fraction present in the MAM, while PLA2G6 exhibits the opposite distribution pattern, being predominantly enriched in MAM compartments with a minor fraction present in the pure mitochondria. Moreover, GRP75 and PLA2G6 proteins are not located in the same submitochondrial compartment. GRP75 is mainly in the mitochondrial matrix, while PLA2G6 is in the inner membrane (Fig. S6a of the revised manuscript, lines 212–227). The distinct subcellular localization patterns of GRP75 and PLA2G6 preclude significant physical interaction at the mitochondrial region. Thus, when IP was performed on crude mitochondrial fractions, the GRP75 protein was highly enriched, while the interaction level between GRP75 and PLA2G6 did not increase significantly. In addition, we perform IP on MAM fractions, the GRP75's co-immunoprecipitation appears significantly increased relative to its presence in the input, compared to IP on crude mitochondrial fractions (Fig. S6b of the revised manuscript). These data suggest that the interaction between PLA2G6 and GRP75 proteins mainly occurs in the MAM region, rather than in mitochondria.

• The data in Fig. 3i do not provide sufficient evidence to convincingly conclude that these proteins form a complex.

Response: Thank you for your valuable criticism. The BN-PAGE experiment has long been used to prove the components of protein complexes (PMID: 26206086, 27995898, 31767755), but a single BN-PAGE experiment is indeed not convincing. Therefore, we also demonstrated that the PLA2G6 protein interacts with the IP3R1-GRP75-VDAC1 complex through PLA and co-IP experiments (Fig. S3a–f of the revised manuscript). In addition, the loss of PLA2G6 disrupted the integrity and function of the IP3R1-GRP75-VDAC1 complex (Fig. 2i, 4a–p of the revised manuscript), which can indirectly demonstrate the association between the PLA2G6 protein and IP3R1-GRP75-VDAC1 complex. We acknowledge that cryo-electron microscopy is currently the best technique for demonstrating protein complexes, but we are very sorry that we do not have the experimental conditions for this technique in our lab now. We will further explore the interrelationship between PLA2G6 and IP3R1-GRP75-VDAC1 complex in our future study if the experimental conditions are suitable.

• Why are signals concentrated near the nuclei in the PLA images of Fig. 4 and Fig. S6?

Response: Thank you for your comment. We think this phenomenon may be related to cell size and nuclear size. Specifically, N2a cells have a relatively small cell volume, and the nucleus occupies most of the intracellular space, leaving a limited cytoplasmic area. This spatial constraint likely results in the majority of PLA signals appearing around the nuclear periphery, but this does not impact the validity of our experimental findings. In addition, many PLA signals are not near the nuclei in the PLA images of Fig. S7 of the revised manuscript. To avoid misunderstanding, we magnified other regions in the representative image that exhibit PLA signal points not concentrated around the nuclear periphery (Fig. S7a of the revised manuscript).

• Is the observed reduction in IP3R1 expression in PLA2G6 KO consistent? Fig. S7b does not show a reduction in IP3R1. Also, why does VDAC1 appear as a doublet in some instances and a single band in others?

Response: Thank you for your comment. To answer your question, we added a GAPDH to quantify the changes in IP3R1 (Fig. S8b of the revised manuscript). The statistical result indicates that the IP3R1 protein level was decreased in the KO and KO + D331Y PLA2G6 group compared to the KO + WT PLA2G6 group. (Fig. R3 related to Fig. S8b, shown below).

The appearance of double bands of VDAC1 is rather random. We consider that the lower band might be a non-specific VDAC1 band. In mammals VDAC is present in 3 highly homologous isoforms: VDAC1, VDAC2, and VDAC3. Given that the molecular weight of VDAC3 is only slightly smaller than that of VDAC1, we suspect that this VDAC1 antibody (Proteintech, 55259-1-AP) may occasionally exhibit weak non-specific recognition of VDAC3. Furthermore, we reviewed literature using this antibody and found some similar observations in previous studies (PMID: 35192817, 35883852). In addition, through the use of VDAC1 siRNA, we found that the band around 35 kDa decreases, and we propose that the band around 35 kDa is specific and the antibody mainly recognize VDAC1 (Fig. R4, shown below).

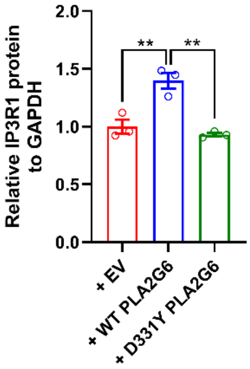

Fig. R3 Relative IP3R1 protein to GAPDH. IP3R1 semi-quantitative statistics. (n = 3 biologically independent experiments). Data are presented as means ± SEM. ** indicate statistical significance at $p < 0.01$. One-way ANOVA with Tukey' s multiple comparison tests was used for statistical analysis.

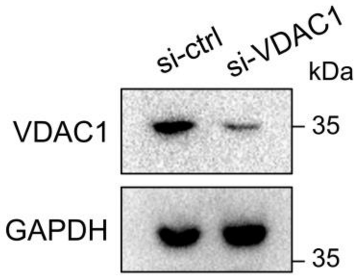

Fig. R4 Representative immunoblot of VDAC1 knockdown. The expression of VDAC1 protein is knockdown by siRNA.

• *If PLA2G6 regulates IP3R1 stability under basal conditions, PLA data for ERLIN2-IP3R1 and RNF170-IP3R1 without CCh stimulation would be necessary. Are data available for ERLIN1? Were cells treated with CCh in Fig. 5k and l?*

Response: Thank you for your valuable comments and suggestions. We have added PLA data for ERLIN1-IP3R1, ERLIN2-IP3R1, and RNF170-IP3R1 with or without CCh stimulation (Fig. 5k, l and S9k, l of the revised manuscript). The cells were treated with CCh in Fig. 5k, l and we have noted CCh on the picture (Fig. 5k of the revised manuscript).

Minor Points:

• *Fig. 2 and Fig. S7 present redundant KO data, and the figure layout could be optimized.*

Response: Thank you for your suggestions. According to your suggestion, we have re-laid out Fig. 2 and Fig. S7 (Fig. 2 and Fig. S8 of the revised manuscript).

• *Why does the introduced PLA2G6 in Fig. S7a differ in molecular weight from the endogenous protein?*

Response: Thank you for your comment. Because pcDNA-PLA2G6 (human)-3×Flag and pcDNA-D331Y PLA2G6 (human)-3×Flag were used. The 3×Flag tags make the introduced PLA2G6 in Fig. S7a (Fig. S8a of the revised manuscript) differ in molecular weight from the endogenous protein.

• *The citations in Lines 185 and 196 referring to Fig. S6 are incorrect and should reference Fig. S5.*
Response: We apologize for any errors, and we have corrected them.

Reviewer #3 (Remarks to the Author):
The study explores the role of PLA2G6 in regulating ER-mitochondrial contact sites, mitochondrial function, and its implications for Parkinson's disease (PD). Employing complementary approaches such as advanced imaging techniques (HIS-SIM, TEM, FIB-SEM), biochemical fractionation, and functional assays, the study investigates PLA2G6 localization and function across in vitro and in vivo models.
The presented data are interesting and relevant.
While the relationship between PLA2G6, mitochondria, and ER-mitochondrial contact sites has been previously documented—particularly the role of PLA2G6 at mitochondria-associated membranes (MAMs) in Drosophila (PMID: 31548400)—the current study should clearly articulate how its findings extend or differ from this prior work to underscore its novel contributions.
Response: Thank you for your helpful suggestion. The article of PMID: 31548400 has demonstrated that neuronal overexpression of MAM protein C19orf12 rescued the heat sensitivity, motor disability, and bang sensitivity of *PLA2G6*$^{-/-}$ flies. In addition, the work showed that the enhancement of MAM integrity rescued the bang-sensitive seizure phenotype of *PLA2G6*$^{-/-}$ flies. These data suggest that there may be a connection between PLA2G6 and MAM. However, this prior work did not demonstrate that the PLA2G6 protein also localizes in the MAM and interacts with the IP3R1/GRP75/VDAC1 complex to regulate the Ca^{2+} transfer from the ER to mitochondria. We have cited this article in our revised manuscript (lines 407–413 of the revised manuscript).

This study strengthens translational relevance by using several complementary models, including human induced pluripotent stem cell-derived systems. To further solidify the findings, additional experiments are recommended, particularly validation of the role of the GRP75/IP3R/VDAC complex in human PD patient-derived cells. This would provide deeper insights and enhance the study's contribution to understanding PLA2G6's role in PD pathogenesis.
Response: Thank you for your critical suggestion. We have compared the alterations of the IP3R1/GRP75/VDAC1 complex between Healthy Control 1 and Patient 1-derived neurons by co-IP and BN-PAGE assays. The result suggests that dopaminergic neurons derived from the patient with PD carrying the *PLA2G6* mutation exhibit decreased IP3R1/GRP75/VDAC1 complex compared to the healthy control-derived neurons (Fig. S12 of the revised manuscript).

The authors present evidence supporting a role for PLA2G6 in regulating mitochondria-associated membrane (MAM) integrity by modulating the IP3R-GRP75-VDAC1 complex through interactions with GRP75. To strengthen the mechanistic understanding, the authors should elaborate on how IP3R1 degradation impacts calcium homeostasis and its downstream effects on neuronal function.
Response: Thank you for your valuable suggestion. We have elaborated on how IP3R1 degradation impacts calcium homeostasis and its downstream effects on neuronal function with red highlights in the Discussion (lines 485–494 of the revised manuscript).

1. The authors propose structural and functional associations between PLA2G6 and GRP75; however, additional experimental validation is necessary to substantiate this claim. Performing co-

immunoprecipitation experiments to confirm the interaction between PLA2G6 and GRP75, while including known interactors of the GRP75/IP3R/VDAC complex as controls, would provide stronger evidence and significantly enhance the robustness of these findings.

Response: Thank you for your valuable suggestion. DJ-1 and GSK3β are known interactors of the GRP75/IP3R/VDAC complex[6,7]. By co-IP experiment, we find that the PLA2G6 protein physically interacts with DJ-1 and GSK3β (Fig. S6c of the revised manuscript).

2. A detailed cellular localization analysis of PLA2G6 and GRP75 within specific ER and mitochondrial subcompartments (e.g., ER lumen, ER membrane, MAM, MOM, MIM, and mitochondrial matrix) would provide critical insights into the spatial basis of these interactions. This analysis could clarify the precise sites of functional relevance. Additionally, Figure 1d is of poor quality, and the claimed finding is not evident. The figure should be improved, with higher resolution and clear annotations, to support the stated results effectively.

Response: Thank you for your constructive suggestions. According to Fig. 1a–c, the GRP75 protein does not localize to the ER. It coexists with PLA2G6 protein in MAM and mitochondria. Therefore, we analyzed the localization of PLA2G6 and GRP75 within the submitochondrial compartments, including the mitochondrial outer membrane (MOM), the mitochondrial inner membrane (MIM), and the mitochondrial matrix. When mitochondria remain intact, only proteins localized to the mitochondrial outer membrane (MOM), such as TOM20, are accessible to Protease K digestion. However, disrupting the MOM with a hypotonic swelling buffer exposes proteins of the mitochondrial inner membrane (MIM), like TIM23, allowing their subsequent degradation by the Protease K. Only after complete membrane lysis with Triton X-100, which led to the digestion of all the mitochondrial proteins by Protease K, including matrix proteins (NDUFA9). The result showed that the GRP75 localizes to the mitochondrial matrix, rather than in MOM or MIM. However, the cellular localization of PLA2G6 proteins is predominantly in the MIM (Fig. S6a of the revised manuscript), suggesting that most of the PLA2G6 proteins coexist and interact with GRP75 in the MAM, but not in submitochondrial compartments.

Thank you for your helpful suggestion. We have improved Fig. 1d.

3. An additional figure where IP3R/VDAC1 PLA is performed in an IP3R or VDAC1 knock-out or knock-down cell line should be included as a negative control.

Response: Thank you for your suggestion. We have constructed stable IP3R1 KO N2a cells by CRISPR-Cas9 (Fig. S7i) and performed IP3R1-GRP75 and IP3R1-VDAC1 PLA in *IP3R1* KO N2a cells as negative controls (Fig. S7j, k).

4. A blot against a non-PLAG2A6 interacting protein should be included to highlight that the IP only detects PLAG2A6 interacting proteins.

Response: Thank you for your suggestion. There are no lysosomal proteins in our mass spectrometry results. Moreover, PLA2G6 has not been reported to interact with lysosomal proteins in previous studies. Therefore, we included LAMP1 as a non-PLA2G6 interacting protein to demonstrate that the IP only detects PLA2G6-interacting proteins (Fig. S6c).

5. To determine whether reduced IP3R1 levels stem from increased degradation, the authors could

consider blocking degradation pathways to clarify if degradation of IP3R1 overexpression impacts PLA2G6 or if there is a reciprocal regulatory mechanism.

Response: Thank you for your comments. Not sure if we understood you correctly. Are you asking us to verify whether the reduction of IP3R1 is due to protein degradation, and whether IP3R1 also affects the protein expression of PLA2G6? We treated cells with the protein synthesis inhibitor CHX. The result showed that *Pla2g6* KO led to accelerated degradation of IP3R1 under normal conditions (Fig. S9g, h of the revised manuscript). In addition, we used MG-132 and 3-MA to block proteasomal and lysosomal degradation pathways, respectively. The result showed that MG-132, but not 3-MA, restored the degradation of IP3R1 in *Pla2g6* KO cells, suggesting that the degradation of IP3R1 due to *Pla2g6* KO is through the proteasome pathway (Fig. S9i, j of the revised manuscript).

To address the second issue, we constructed stable *IP3R1* KO N2a cells and transiently overexpressed IP3R1-GFP in control cells. We found that the expression of PLA2G6 is not regulated by IP3R1 (Fig. S9a–d).

6. While the study emphasizes IP3R1-mediated Ca^{2+} transfer, the lack of significant changes in PKD2- or ryanodine receptor-mediated Ca^{2+} flux could be better discussed to refine PLA2G6's specificity in regulating IP3R1-dependent processes.

Response: Thank you for your helpful suggestion. We have discussed the lack of significant changes in PDK2- or ryanodine receptor-mediated Ca^{2+} flux in the Discussion section (lines 429–434 of the revised manuscript).

Minor

- The Methods section should clarify the methods used to quantify mitochondrial morphology and MAM.

Response: Thank you for raising this important methodological point. We have clarified the methods used to quantify mitochondrial morphology and MAM in the Method section (lines 1057–1067 and 1090–1104 of revised manuscript).

- The authors should provide a more comprehensive characterization of iPSC differentiation across the different lines by including additional relevant markers for validation. Furthermore, presenting low-magnification images would help assess the overall differentiation efficiency and uniformity, thereby enhancing the rigor and clarity of the study.

Response: Thank you for your suggestions. We have added TUJ-1, MAP2, FOXA2, GIRK2, and NURR1 for validation, and present low-magnification images (Fig. S10c).

Reviewer #4 (Remarks to the Author):

The authors used transmission electron microscopy (TEM) and focused ion beam-scanning electron microscopy (FIB-SEM) to observe and analyze inter-organelle contacts, which is a fascinating area of research. However, there are several aspects where I would appreciate further clarification.

Major comments:

1. FIB-SEM Imaging and Segmentation Methodoloogy: The manuscript mentions the image processing steps of "alignment, filtering, and manual segmentation" but lacks a detailed explanation

of the specific methods or algorithms employed. It is unclear whether automated segmentation algorithms were used or if the segmentation process relied on manual intervention. If manual segmentation was utilized, how was consistency ensured across different datasets? Additionally, the resolution of the images in terms of lateral (horizontal) resolution should be clarified to provide a better understanding of the imaging quality.

Response: Thank you for raising these important methodological points. We have elaborated the FIB-SEM images processing steps, and we segmented images by manual process (lines 1090–1104 of the revised manuscript). To ensure consistency across different datasets, we strictly traced the mitochondrial outer membrane and ER membrane to generate surface reconstructions, and the analyzer was blinded to the experimental conditions. The voxel size of images is 2.4847 × 2.4847 × 10 nm (resolution).

2. Super-resolution Imaging and Co-localization Analysis: While the manuscript describes the use of super-resolution imaging for co-localization analysis, the description of the software and the specific analysis methods employed could be strengthened. It would be helpful to provide more detail on the computational tools or algorithms used to quantify co-localization, as well as how potential issues such as background noise and image artifacts were addressed during the analysis.

Response: Thank you for your suggestions. Image processing is done using Wiener Reconstruction. The specific parameter information is as follows: defocus elimination 5, notch filter 10, 3D Wiener parameter 0.5. In addition, we used the Gaussian Filter to reduce the background noise before segmentation. Mitochondria and ER segmentation are done using Surface Creation and Filament Diameter, respectively. Finally, Pearson's Correlation Coefficient was used to quantify the co-localization between ER and mitochondria (lines 953–958 of the revised manuscript).

3. ER-mitochondria Contact Sites: What detailed methods were used to calculate the ER-mitochondria (also including ER-lysosome and mitochondria-lysosome) contact sites? Specifically, what hyperparameters of the contact site calculator in the Amira software did the authors implement? For example, how close must two objects be to be considered a contact site? If this threshold is changed, will the results change? Multiple softwares (Amira, Imaris, ImageJ) were used. Did they use the same quantification method or criterion? Understanding the methodology behind these calculations is crucial for interpreting the results accurately.

Response: Thank you for your important comments. The ER-mitochondria, ER-lysosome, and mitochondria-lysosome contacts (Fig. 2a, S1e, g) were calculated by Imaris. The specific methods refer to Question 2 (lines 953–958 of the revised manuscript). The criteria of Amira and ImageJ are the same. The criteria of ER-mitochondria contact sites are defined as: the parallel membrane alignment ≤ 30 nm in images of electron microscopy. Detailed methods for all three software analyses of ER-mitochondria contact sites have been added to Methods section (953–958, 1057–1067, and 1090–1104 of the revised manuscript). If the criteria are changed, the results may be affected. However, a parallel membrane alignment ≤ 30 nm is a commonly recognized criterion in electron microscopy images[6,8].

4. The Morphology of ER: As shown in Figures 2 c and f, it seems that there are not only differences between the ER-mitochondria contact sites, but also differences between ER morphology. For example, the diameter of the tubular structure formed by the ER (or MAM).

Response: We appreciate your detailed review. We similarly found in the course of our study that *Pla2g6* KO may cause expansion of the ER. The previous study has reported that ER morphology does not affect the ER-mitochondria contact, so we suspect that *Pla2g6* KO-induced reduction in ER-mitochondria contacts and ER swelling may be mutually independent phenotypes. We will explore the effect of PLA2G6 on ER morphology in future studies.

5. Results: The article appears to focus solely on the area of contact surfaces, noting that "in Pla2g6 KO N2a cells, the size of ER-mitochondria contacts decreased by 38.5% compared to control cells (Fig. 2g)." However, Figure 2f indicates that individual mitochondria have multiple contact sites. How might the analysis of changes in the number of contact sites contribute to the understanding of this phenomenon? Could it occur that, despite a potential reduction in the overall contact area (i.e., the sum of all contact surfaces), the area of each individual contact site may increase? This phenomenon could arise as a result of a decrease in the number of contact points. An analysis of both surface area and contact site quantity could provide deeper insights.

Response: Thank you for your insightful comments. According to your suggestion, we have analyzed the number of contact sites. The *Pla2g6* KO led to a reduction in the number of contact sites (Fig. R5 related to Fig. 2f, shown below). But unlike your view, we think that the surface of each contact site, but not the overall contact area, decreases by 38.5% in *Pla2g6* KO cells compared to control cells, according to Figure 2g. Therefore, in our study, each contact site does not increase. In addition, we think that when the lengths of ER-mitochondria contacts are reduced to a certain level (contact distance > 30 nm), there are naturally fewer contact points. Thus, we propose that the decrease in contact number reflects a marked reduction in each ER-mitochondrial contact. In other words, the reduction in the number of contacts is the consequence of a significant reduction in contact lengths or contact surfaces.

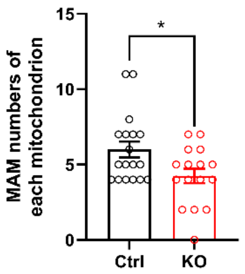

Fig. R5 MAM numbers of each mitochondrion of control and *Pla2g6* KO cells. Measurement of MAM numbers in control and Pla2g6 KO cells (control, n = 18 mitochondria; KO, n = 16 mitochondria from three independent experiments). * Indicates statistical significance at $p < 0.05$. Two-tailed unpaired t-tests were used for statistical analysis.

Minor comments:

Finally, a minor discussion point: the morphology of mitochondria is closely related to both health and disease states [1]. Do the authors anticipate that variations in mitochondrial shape could influence the contact area and the number of contact sites? Exploring this relationship may add an interesting dimension to the current study.

Response: Thank you for your suggestion. We have discussed the effects of mitochondrial morphological alterations on ER-mitochondrial contacts and cited the article (lines 437–440 of the

revised manuscript).

I look forward to the authors' responses to these queries, which I believe will enhance the clarity and depth of the manuscript.

[1] Yapa N M B, Lisnyak V, Reljic B, et al. Mitochondrial dynamics in health and disease[J]. FEBS letters, 2021, 595(8): 1184-1204.

1 Yang, Z., Zhao, X., Xu, J., Shang, W. & Tong, C. A novel fluorescent reporter detects plastic remodeling of mitochondria - ER contact sites. *Journal of Cell Science* **131**, doi:10.1242/jcs.208686 (2018).

2 Friedman, J. R. *et al.* ER tubules mark sites of mitochondrial division. *Science* **334**, 358-362, doi:10.1126/science.1207385 (2011).

3 Lin, G. *et al.* Phospholipase PLA2G6, a Parkinsonism-Associated Gene, Affects Vps26 and Vps35, Retromer Function, and Ceramide Levels, Similar to alpha-Synuclein Gain. *Cell Metab* **28**, 605-618 e606, doi:10.1016/j.cmet.2018.05.019 (2018).

4 Zhou, Q. *et al.* Impairment of PARK14-dependent Ca(2+) signalling is a novel determinant of Parkinson's disease. *Nat Commun* **7**, 10332, doi:10.1038/ncomms10332 (2016).

5 Engel, L. A., Jing, Z., O'Brien, D. E., Sun, M. & Kotzbauer, P. T. Catalytic function of PLA2G6 is impaired by mutations associated with infantile neuroaxonal dystrophy but not dystonia-parkinsonism. *PLoS One* **5**, e12897, doi:10.1371/journal.pone.0012897 (2010).

6 Liu, Y. *et al.* DJ-1 regulates the integrity and function of ER-mitochondria association through interaction with IP3R3-Grp75-VDAC1. *Proc Natl Acad Sci U S A* **116**, 25322-25328, doi:10.1073/pnas.1906565116 (2019).

7 Gomez, L. *et al.* The SR/ER-mitochondria calcium crosstalk is regulated by GSK3beta during reperfusion injury. *Cell Death Differ* **23**, 313-322, doi:10.1038/cdd.2015.101 (2016).

8 Kim, S., Coukos, R., Gao, F. & Krainc, D. Dysregulation of organelle membrane contact sites in neurological diseases. *Neuron* **110**, 2386-2408, doi:10.1016/j.neuron.2022.04.020 (2022).

Reviewer #1 (Remarks to the Author):

There are three major concerns that remain unaddressed. The original concerns are listed under "Unaddressed Concern" sections and my new comments are listed in the "Comment on the authors' response to this concern" section.

Unaddressed Concern 1:
ER and mitochondrial abnormalities have previously been associated with PLA2G6 loss-of-function models. Hence, the structural changes observed at the mitochondria-associated membranes (MAMs) could be secondary to broader disruptions in ER and/or mitochondrial architecture. Two unresolved issues underscore this concern. First, in the original Fig. 1d, PLA2G6 was shown to colocalize predominantly with mitochondrial markers and only partially with ER markers, rather than being specifically enriched at the MAM. This finding directly contradicts the authors' claim of a specific role for PLA2G6 at the MAM.

Comment on the authors' response to this concern:
To address this concern, the authors replaced the images in Fig. 1d. The original figure displayed a relatively broad distribution of PLA2G6 across mitochondria and some ER, rather than a specific enrichment at MAMs. However, in the revised images, PLA2G6 staining is confined to the MAM, with no apparent signal in the surrounding ER or mitochondria. This not only contradicts their original data but also conflicts with the published literature, raising serious concerns.

Unaddressed Concern 2:
To follow up on the previous concern, the changes presented in Fig. 2 regarding MAM structure are subtle. For example: the difference in 'Length of each MAM' between control and knockout cells is minimal, with only two data points showing variation in the control group. Similarly, the reported reduction in 'Size of each MAM' is modest. These marginal differences raise concerns about the robustness and biological relevance of the proposed MAM phenotype resulting from PLA2G6 loss.

Comment on the authors' response to this concern:
In the revised manuscript, the authors addressed the role of MAMs in Ca^{2+} transfer. However, they did not provide a clear explanation or additional data to support a biological role for PLA2G6 in regulating MAM morphology. The reported increase in 'Length of each MAM' appears to be driven primarily by two outliers, while the majority of data points show no significant difference. Similarly, the reported reduction in 'Size of each MAM' is modest.

Unaddressed Concern 3:
Previous studies have shown that PLA2G6[ko/ko] mice typically exhibit detectable defects around 1 year of age. At 3 months, no obvious mitochondrial morphological defects have been reported (PMID: 27030050). However, in Fig. 2j, the authors show obvious mitochondrial morphological defects in the dopaminergic neurons of the 3-month-old PLA2G6[ko/ko] mice. Can the authors provide an explanation for this discrepancy? Are mitochondria in dopaminergic neurons more sensitive to PLA2G6 loss?

Comment on the authors’ response to this concern:
In this revised version, the authors also have not provided any explanation or additional data to address this.

Reviewer #2 (Remarks to the Author):

The revised manuscript presents significantly improved organelle imaging, which in turn enhances the persuasiveness of the authors’ claims.

An important new finding in this revision is that the lipase activity of PLA2G6 does not appear to be related to the phenotypes observed by the authors. Due to the lack of definitive information on the physiological substrates of PLA2G6, the relationship between its lipase activity and disease-associated mutations remains unclear. Although previous reports have assessed lipase activity of disease-associated mutants using artificial substrates, the correlation between missense mutations and enzymatic activity has been inconsistent (PMID: 20886109, PMID: 21700586). To clarify the relationship between IP3R1 stability and the disease relevance, this reviewer recommends that the authors investigate the correlation between the observed Ca^{2+} dynamics (and/or IP3R1 levels) and several known pathogenic PLA2G6 variants. In particular, it would be valuable to determine whether there are differences between INAD/NBIA-related mutations and PD-associated mutations. Furthermore, clarifying which specific domain(s) of PLA2G6 are essential for MAM formation would substantially improve the quality of the manuscript.

Below are some minor points:
•Regarding the raw data for the mass spectrometry analysis, I was able to confirm the Excel dataset included in this manuscript; however, I could not locate PXD057620 in the PRIDE website.

•In the legend of Fig. S6, it states “n = 3 independent experiments.” Could the authors clarify whether this refers to representative results from three independent experiments?

•In the legend of Fig. S6b, it reads “Immunoblotting analysis of GRP7.” Please confirm whether this is a typographical error.

Reviewer #3 (Remarks to the Author):

The Authors have address all our previous concerns.

Reviewer #4 (Remarks to the Author):

I appreciate the authors' efforts in revising the manuscript. The additional technical details and discussions adequately address my concerns raised in the previous round. I find the current version improved and more complete. I have no further major comments.

First of all, we sincerely express our gratitude to the editor and reviewers for their time, positive comments and helpful advices. Specifically, we have addressed these comments and concerns in a point-by-point manner as follows. The original comments appear in *italics*, with our corresponding responses in standard font and blue highlighting. The edits in the manuscript are presented in regular text with red highlights.

Reviewer #1 (Remarks to the Author):

There are three major concerns that remain unaddressed. The original concerns are listed under "Unaddressed Concern" sections and my new comments are listed in the "Comment on the authors' response to this concern" section.

Unaddressed Concern 1:
ER and mitochondrial abnormalities have previously been associated with PLA2G6 loss-of-function models. Hence, the structural changes observed at the mitochondria-associated membranes (MAMs) could be secondary to broader disruptions in ER and/or mitochondrial architecture. Two unresolved issues underscore this concern. First, in the original Fig. 1d, PLA2G6 was shown to colocalize predominantly with mitochondrial markers and only partially with ER markers, rather than being specifically enriched at the MAM. This finding directly contradicts the authors' claim of a specific role for PLA2G6 at the MAM.
Comment on the authors' response to this concern:
To address this concern, the authors replaced the images in Fig. 1d. The original figure displayed a relatively broad distribution of PLA2G6 across mitochondria and some ER, rather than a specific enrichment at MAMs. However, in the revised images, PLA2G6 staining is confined to the MAM, with no apparent signal in the surrounding ER or mitochondria. This not only contradicts their original data but also conflicts with the published literature, raising serious concerns.
Response: Thank you for your insightful comments on Fig. 1d. We understand your concerns about the change in the localization of PLA2G6, including the potential contradictions with the original data and the published literature. However, since the ER did not show a reticular structure and the mitochondria did not show a rod-shaped structure in the previous Fig. 1d, we believe that the specificity of the antibody staining results is not very high. Therefore, the results cannot accurately reflect the localization relationship among PLA2G6, the ER, and the mitochondria. Therefore, we used the method of transfecting fluorescent plasmids. From the revised Fig. 1d, we can see that the morphologies of the ER and mitochondria are more accurate. Therefore, we believe that the revised Fig. 1d can more accurately reflect the localization relationship among PLA2G6, ER, and mitochondria.
In revised Fig. 1d, to better show the localization of the PLA2G6 protein in the MAM, we specifically magnified a representative local area. This may lead you to misunderstand that PLA2G6 staining is confined to the MAM. However, from the panoramic view of fig. 1d, we can see that PLA2G6 is also distributed in the ER and mitochondria simultaneously, which is consistent with the conclusions of published literature and the conclusions of Fig. 1a-c.
In addition, we highly agree with your view that PLA2G6 is distributed in the ER and mitochondria, not being specifically enriched at the MAM. This paper discovers novel localization

of PLA2G6 protein in the MAM and demonstrates its function in regulating Ca^{2+} transport in the MAM, without denying its other functional roles in the ER or mitochondria. We elaborated these aspects in the Discussion section (lines 491–497 of the revised manuscript).

Unaddressed Concern 2:

To follow up on the previous concern, the changes presented in Fig. 2 regarding MAM structure are subtle. For example: the difference in 'Length of each MAM' between control and knockout cells is minimal, with only two data points showing variation in the control group. Similarly, the reported reduction in 'Size of each MAM' is modest. These marginal differences raise concerns about the robustness and biological relevance of the proposed MAM phenotype resulting from PLA2G6 loss.

Comment on the authors' response to this concern:

In the revised manuscript, the authors addressed the role of MAMs in Ca^{2+} transfer. However, they did not provide a clear explanation or additional data to support a biological role for PLA2G6 in regulating MAM morphology. The reported increase in 'Length of each MAM' appears to be driven primarily by two outliers, while the majority of data points show no significant difference. Similarly, the reported reduction in 'Size of each MAM' is modest.

Response: We feel sorry that we do not provide a clear explanation to support a biological role for PLA2G6 in regulating MAM morphology. To demonstrate the important role of PLA2G6 in regulating MAM morphology, we also introduced the MAM Linker in *Pla2g6* KO cells, which can promote MAM formation. Our results showed that MAM Linker can partially restore the mitochondrial perimeter *Pla2g6* KO cells (Fig. S5f of the revised manuscript), suggesting that the increase in mitochondrial perimeter caused by the deficiency of PLA2G6 is partially due to the decrease of MAM.

In addition, even if we removed two outliers as you suggested, we found that the comparison between the control group (183.4 ± 7.7 nm) and the KO group (145.2 ± 6.6 nm) was still less than 0.001 ($p = 0.0002$, Fig. R1 related to Fig. 2c, shown below).

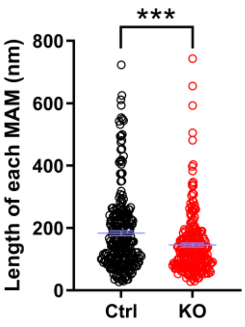

Fig. R1 The length of each MAM in control and Pla2g6 KO cells after removing two outliers.

Moreover, in Fig.2g, the size of each MAM in the control group is 32275±4026 nm^2, but the size of each MAM in the control group is only 19862±3235 nm^2. The statistical difference between the two groups is less than 0.001 ($p < 0.001$).

Unaddressed Concern 3:

Previous studies have shown that PLA2G6[ko/ko] mice typically exhibit detectable defects around

1 year of age. At 3 months, no obvious mitochondrial morphological defects have been reported (PMID: 27030050). However, in Fig. 2j, the authors show obvious mitochondrial morphological defects in the dopaminergic neurons of the 3-month-old PLA2G6[ko/ko] mice. Can the authors provide an explanation for this discrepancy? Are mitochondria in dopaminergic neurons more sensitive to PLA2G6 loss?

Comment on the authors' response to this concern:

In this revised version, the authors also have not provided any explanation or additional data to address this.

Response: Thank you for your insightful comment. I apologize that my previous reply may not have clearly explained this issue, leading to a misunderstanding. In our initial experiments using transmission electron microscopy (TEM), we could only distinguish neurons from glial cells under TEM, but could not distinguish different neuronal subtypes. Therefore, we may compare mitochondria from different neuronal subtypes, which leads to significant mitochondrial morphological differences observed between the two groups.

To solve this problem, we employed immuno-EM. By labeling the dopaminergic neuron-specific protein tyrosine hydroxylase (TH) and then specifically comparing the MAM and mitochondria in TH-positive neurons, we found that there was no significant difference in mitochondrial morphology between 3-month-old control and KO mice. Thus, mitochondria of dopaminergic neurons are not more sensitive to PLA2G6 loss than other neuronal types. However, the phenotype of reduced MAM had already appeared in dopaminergic neurons of 3-month-old KO mice compared to control mice. This also suggests that PLA2G6 KO may first lead to a reduction in MAM, which in turn causes abnormal mitochondrial morphology.

Reviewer #2 (Remarks to the Author):

The revised manuscript presents significantly improved organelle imaging, which in turn enhances the persuasiveness of the authors' claims.

An important new finding in this revision is that the lipase activity of PLA2G6 does not appear to be related to the phenotypes observed by the authors. Due to the lack of definitive information on the physiological substrates of PLA2G6, the relationship between its lipase activity and disease-associated mutations remains unclear. Although previous reports have assessed lipase activity of disease-associated mutants using artificial substrates, the correlation between missense mutations and enzymatic activity has been inconsistent (PMID: 20886109, PMID: 21700586). To clarify the relationship between IP3R1 stability and the disease relevance, this reviewer recommends that the authors investigate the correlation between the observed Ca^{2+} dynamics (and/or IP3R1 levels) and several known pathogenic PLA2G6 variants. In particular, it would be valuable to determine whether there are differences between INAD/NBIA-related mutations and PD-associated mutations. Furthermore, clarifying which specific domain(s) of PLA2G6 are essential for MAM formation would substantially improve the quality of the manuscript.

Response: Thank you for your valuable suggestion. The PLA2G6 protein is composed of ankyrin repeat (ANK) domains and patatin-like phospholipase (PNPLA) domain (refer to the UniProt). To determine which specific domain(s) of PLA2G6 are essential for MAM formation, we generated

ANK and PNPLA truncated mutants. Overexpression of WT or PNPLA truncated mutant (ΔPNPLA) in PLA2G6 KO cells restores the ER-mitochondria contacts, but this interaction was not rescued by ANK truncated mutant (Fig. S3a–e and lines of 150–157). This result suggests that the ANK domain of the PLA2G6 protein is essential for MAM formation. The previous research on crystal structure revealed that the ANK domain surrounds the catalytic domain (PNPLA domain) and adopts an outwardly flared orientation, poised to interact with membrane proteins[1]. This special conformation of the ANK domain may be the key to maintaining the stability of MAM.

In addition, we have assessed whether PD-associated mutants (D331Y, R741Q) and INAD-associated mutants (G517C, G638R) impact Ca^{2+} transfer and IP3R1 levels. The result showed that re-expressing D331Y in KO cells did not affect Ca^{2+} transfer or IP3R1 levels. However, re-expression of INAD-associated mutants (G517C, G638R) and the PD-associated mutant (R741Q) upregulated both Ca^{2+} transfer and IP3R1 levels (Fig. S10c–f of revised manuscript). This suggests that mutations at different sites within the PLA2G6 gene may lead to distinct pathogenic mechanisms. The R741Q mutant does not impact the IP3R1 level and mitochondrial Ca^{2+} transfer, which may be attributed to the fact that the R741Q mutant does not affect the ANK domain. Moreover, different pathogenic mechanisms may explain the differences in the severity of PD phenotypes caused by D331Y and R741Q. We have discussed this result in the Discussion (lines 527–538 of the revised manuscript).

Below are some minor points:

•Regarding the raw data for the mass spectrometry analysis, I was able to confirm the Excel dataset included in this manuscript; however, I could not locate PXD057620 in the PRIDE website.

Response: We are very sorry that we did not make it clear how to search for PXD057620 on the PRIDE website. Since the article has not been published yet, the raw data is not publicly available at present, so you cannot directly search for PXD057620 on the website. You can access the dataset by logging in to the PRIDE website (http://www.ebi.ac.uk/pride) using the following account:

Username: reviewer_pxd057620@ebi.ac.uk

Password: Ofqi3uXwpBd4

•In the legend of Fig. S6, it states "n = 3 independent experiments." Could the authors clarify whether this refers to representative results from three independent experiments?

Response: Yes. The experiments of Fig.S7 of the revised manuscript (original Fig.S6) were all independently performed three times. The representative results in Fig.S7 are from three independent experiments.

•In the legend of Fig. S6b, it reads "Immunoblotting analysis of GRP7." Please confirm whether this is a typographical error.

Response: Thank you for the reminder. This is a typographical error, and we have corrected it.

Reviewer #3 (Remarks to the Author):

The Authors have address all our previous concerns.

Response: I appreciate your acknowledgment of the revised version.

Reviewer #4 (Remarks to the Author):

I appreciate the authors' efforts in revising the manuscript. The additional technical details and discussions adequately address my concerns raised in the previous round. I find the current version improved and more complete. I have no further major comments.

Response: Thank you for your time and valuable suggestions, which have helped us enhance the quality of the work.

Reference:

1 Malley, K. R. *et al.* The structure of iPLA(2)beta reveals dimeric active sites and suggests mechanisms of regulation and localization. *Nat Commun* **9**, 765, doi:10.1038/s41467-018-03193-0 (2018).

Reviewer #1 (Remarks to the Author):

The authors have addressed my concerns.

Reviewer #2 (Remarks to the Author):

The authors have made efforts to elucidate the correlation between pathogenic variants of PD and NBIA and Ca^{2+} dynamics, and this reviewer believes the overall quality of the manuscript has improved as a result. However, in Figure S3c–f, it remains impossible to compare the calcium dynamics of each mutant with those of wild-type PLA2G6. In other words, a "KO + WT" experimental group should have been included within the same experimental system. Considering the potential effects of disease-associated variants, it is entirely plausible that the results could show “partially impaired" rather than completely abolished function.

First of all, we sincerely express our gratitude to the editor and reviewers for their time, positive comments and helpful advices. Specifically, we have addressed these comments and concerns in a point-by-point manner as follows. The original comments appear in *italics*, with our corresponding responses in standard font and blue highlighting. The edits in the manuscript are presented in regular text with red highlights.

Reviewer #1 (Remarks to the Author):

The authors have addressed my concerns.
Response: I appreciate your acknowledgment of the revised version.

Reviewer #2 (Remarks to the Author):

The authors have made efforts to elucidate the correlation between pathogenic variants of PD and NBIA and Ca^{2+} dynamics, and this reviewer believes the overall quality of the manuscript has improved as a result. However, in Figure S3c–f, it remains impossible to compare the calcium dynamics of each mutant with those of wild-type PLA2G6. In other words, should have been included within the same experimental system. Considering the potential effects of disease-associated variants, it is entirely plausible that the results could show “partially impaired" rather than completely abolished function.
Response: Thank you for your valuable suggestion. If I understand correctly, you should be referring to fig. S10c–f. To compare the Ca^{2+} dynamics and IP3R1 level of each mutant with WT PLA2G6, we have included a "KO + WT" experimental group by the same experimental system. We found that re-expressing the D331Y mutant in KO cells showed no significant difference in Ca^{2+} transfer or IP3R1 levels compared to empty vector-transfected KO controls, with both being lower than KO cells expressing WT PLA2G6. In contrast, re-expression of INAD-associated mutants (G517C, G638R) or the PD-associated mutant (R741Q) upregulated Ca^{2+} transfer and

IP3R1 levels to levels comparable to those observed in KO cells expressing WT PLA2G6 (Fig. S10c–f and lines of 344–351).
